# Care across the gender spectrum: A transgender health curriculum in the Obstetrics and Gynecology clerkship

**DOI:** 10.1186/s12909-022-03766-0

**Published:** 2022-10-05

**Authors:** Christina N. Schmidt, Monica Stretten, Jay G. Bindman, Gaetan Pettigrew, Jeannette Lager

**Affiliations:** 1 School of Medicine, University of California San Francisco, San Francisco, USA; 2 Department of Obstetrics, Gynecology and Reproductive Services, University of California San Francisco, San Francisco, USA; 3 513 Parnassus Ave, S-221, San Francisco, CA USA

**Keywords:** Gender-affirming care, Transgender, Gender diverse, Undergraduate medical education

## Abstract

**Background:**

A lack of undergraduate medical curricula on providing healthcare to transgender and gender diverse (TGD) patients has contributed to significant health disparities for TGD communities. To address this gap, we designed and evaluated a novel curriculum to train Obstetrics and Gynecology (OB/GYN) clerkship students in caring for TGD patients.

**Methods:**

Following Kern’s 6-step method for curriculum development, we created a two-part curriculum on TGD healthcare topics – an online module on gender-affirming care, followed by a series of interactive cases on TGD-specific health topics. Undergraduate medical students completing their core OB/GYN clerkships at a university academic medical center (January-December 2021) were invited to complete this curriculum. Participants completed pre/post assessment surveys to assess their experience caring for TGD patients, as well as a scored knowledge assessment before and after completing the curriculum.

**Results:**

Sixty-five students participated in this curricular assessment. Prior to completing the module, 45% agreed that they had received adequate TGD health training. Following module completion, students reported increased comfort in caring for transgender patients (49.2% vs. 81.5%; p < .001) and endorsed an improved fund of knowledge of both healthcare maintenance for TGD patients (61.5% vs. 100%; p < .001) and gender affirming medical therapies (60.0% vs. 96.9%; p < .001). Knowledge scores increased from a mean of 9.65 (1.81) to 12.5 (2.20) out of 15 (p < .001). In post-assessment surveys, 95% of participants agreed that the module was helpful for their learning. Qualitatively, students suggested longitudinal integration of TGD-topics into the pre-clinical curriculum, and expanded opportunities to practice patient counseling.

**Conclusion:**

The findings of this study support the need for student education on TGD health. Integration of interactive, case-based TGD-care curricula into clinical training may increase medical students’ knowledge and comfort in caring for TGD patients. Ongoing efforts to integrate TGD health training into undergraduate medical student curricula are necessary.

**Supplementary Information:**

The online version contains supplementary material available at 10.1186/s12909-022-03766-0.

## Background

Transgender and gender diverse (TGD) individuals experience significant health disparities due to multifactorial causes. The discrimination, social marginalization and minority stress experienced by TGD individuals is widespread and persists within healthcare spaces [1, 2]. In a large survey of transgender individuals in the United States, almost half reported encountering discrimination while seeking healthcare services [3]. TGD patients who exist at the intersection of multiple marginalizing social forces – including racism or ableism – experience increased rates of health disparities, and 68% of TGD people of color report encountering discrimination while seeking health care [3]. In Obstetrics and Gynecology (OB/GYN), the prolific use of gender-exclusive terms such as “women’s health” contributes to the harm experienced by TGD patients in clinical settings [4, 5]. Negative experiences in healthcare setting have a meaningful impact on access to care, and one in five TGD individuals have avoided seeking care due to fear of mistreatment [6]. Inconsistent access to gender-affirming care for TGD individuals contributes to poorer health outcomes, including increased risk of undesired pregnancies, [7]. sexually transmitted infections, [8, 9]. late-stage cancers, [10]. and unmonitored hormone therapy [11, 12].

There are numerous teaching gaps in undergraduate medical education on the healthcare needs of TGD patients. Traditional medical education curricula employ a binary framework for sex and gender throughout medical teaching and conflate the concepts of sex and gender despite their distinct definitions. Sex assigned at birth is based on anatomy, hormones and chromosomes, while gender broadly encompasses an individual’s identity (their experience of their own gender) and expression (how they present themselves to the world). In addition, medical training programs have stigmatized and pathologized TGD patients who exist outside of these binary frameworks [13, 14]. While non-inclusive teaching structures may adequately prepare physicians to meet the healthcare needs of cisgender patients (whose genders matches their sex assignments at birth), they offer inadequate teaching on the healthcare needs of TGD patients. TGD patients have distinct needs related to their gender identities, hormonal exposures, and organ inventory, warranting the creation of new medical education curricula to provide gender affirming teaching on the needs of these patients.

In clinical practice, TGD patients report lack of provider competency regarding their health care needs as a major driver of negative care experiences [12, 15]. In a study conducted with 141 OB/GYN physicians across the country, only a third reported feeling comfortable caring for transgender patients [16]. Similarly, in a survey of all residents at OB/GYN training programs in the United States, only 22% felt competent to provide trans-relevant services and 90% desired additional clinical training [17]. Recent publications have identified numerous strategies to integrate teaching on the health care needs of TGD patients into medical training, and preliminary evidence suggests that medical students feel more prepared to care for TGD patients when exposed to specific and dedicated curricula on transgender health [18–20]. Despite clear benefits for both patients and providers, very few accredited medical schools and residency programs have robust curricula covering TGD topics [21, 22].

To increase trainee competency in working with TGD patients, medical students and OB/GYN faculty partnered with transgender patients and faculty to develop a novel curriculum on gender affirming care for medical students on their OB/GYN rotation. In this study, we describe our approach to creating a new curriculum on TGD patient care focused on a gender affirmation model, as well as strategies used to assess this curriculum’s efficacy in increasing students’ confidence and competency in working with TGD patients.

## Methods

Using Kern’s 6-step method for curriculum development, we created a new curriculum on providing gender-inclusive care to TGD patients for third-year medical students rotating on their OB/GYN clinical clerkships [23]. Applicable to this current curriculum, we used Kern’s framework to identify educational needs, refine objectives and develop curricular content.

### Steps 1 and 2: Problem identification and needs assessment

A targeted needs assessment, including a review of course evaluations and focus groups with medical students and faculty, identified gaps in the OB/GYN curriculum related to TGD-specific healthcare at our institution. We initially conducted two focus groups (n = 9) with transgender medical students and cisgender student allies with experience working with transgender patients. These focus groups highlighted content that should be reformed, and concepts missing in the existing curriculum. To compliment student feedback, transgender-identified researchers, as well as faculty caring for TGD patients clinically, were consulted on key teaching points to convey to clerkship students within the OB/GYN clerkship didactic curriculum.

Through these early conversations with student and faculty stakeholders with expertise in transgender health, we identified key areas in need of dedicated teaching and used these findings as the framework upon which to build goals and objectives for our transgender health curriculum. Identified gaps included broader coverage of gender-inclusive language, primary care guidelines, gender-affirming medical therapies and interventions, and providing holistic care for families. Our focus groups also highlighted the need to reframe existing content to reinforce best practices for validating the identities of TGD patients and promote gender-affirming healthcare delivery.

Following our initial identification of the gaps in curricular content, we reviewed all materials in the pre-clinical and clinical OB/GYN training courses at our institution, to assess the current state of TGD care inclusion in our existing curricula. At our institution, clinical training on TGD care was previously in the form of a “transgender health module,” which included a series of three cases that students submitted to course administrators upon completing their OB/GYN rotation. These cases were reviewed by senior medical students, as well as transgender faculty, students, and community members. The first broad theme that emerged from this in-depth review was that the existing curriculum pathologized patients’ gender identities through stigmatizing language and assumptions of mental health diagnoses including gender dysphoria, rather than emphasizing gender-affirming care practices. Current materials also lacked content addressing missteps, assumptions, and discrimination experienced by TGD individuals in clinical settings.

### Step 3: Developing goals and objectives

The primary goal of our redesigned curriculum was to fill existing gaps in the students’ foundational knowledge of providing gender-inclusive care in OB/GYN settings. Based on our needs assessment, we generated three guiding principles for the content of our new curriculum: (1) validating the identities of patients, (2) providing gender-affirming care, and (3) reframing the focus to diversity, not pathology. These guiding principles directly lead to the creating of a series of learning objectives for our curriculum: (1) define key vocabulary pertinent to the care of transgender, non-binary, non-conforming and gender expansive people, (2) describe eligibility criteria for hormonal and surgical interventions in adolescents and adults, (3) understand the available gender affirming medical interventions, and (4) understand the unique points of clinical care for transgender individuals.

### Step 4: Educational strategies

#### Instructional strategies

We designed an asynchronous online curriculum for students to complete over the course of their OB/GYN clinical rotation. The curriculum incorporated multiple learning modalities which included traditional lectures, interactive cases, and knowledge assessments. A 30-minute recorded lecture provided important background information for students in a traditional didactic format. Students then completed an interactive, case-based module building upon the foundational concepts introduced in the lecture [24]. The interactive cases were designed to be administered in a remote online format, [25]. which was vital for a curriculum serving students rotating across multiple clinical sites. Knowledge assessments were included as a part of the curricular design, to allow students to check their understanding of the core concepts introduced across the curriculum.

#### Curricular content

The recorded lecture for students covered various TGD-care topics. The lecture began with an overview of important definitions and vocabulary, followed by discussion of general principles for providing gender-inclusive care. Subsequent sections included information on gender-affirming treatments and procedures, eligibility and insurance, and family planning and fertility. The lecture ended with an overview on important family resources (Appendix A).

The series of five interactive cases reinforced concepts introduced in the didactic portion of the curriculum. Cases comprised of clinical vignettes, with multiple choice and free response boxes to reinforce module topics. Topics covered included Tanner staging, eligibility criteria for puberty blockers, documentation of gender identity and pronouns in the electronic health record, healthcare maintenance and providing gender-affirming resources to patients and families. As students progressed through the cases, key learning points and understanding checkpoints highlighted important take-aways at each step of a case. This case-based portion of this curriculum was administered over Qualtrics, allowing students to answer multiple choice questions and free response prompts dynamically. A copy of these cases can be found in Appendix B.

### Step 5: Implementation

Prior to implementing the series of curricular modules, content was reviewed by a transgender medical student (n = 1), faculty with expertise in TGD care (n = 2), a transgender faculty member (n = 1), and a transgender patient/community member (n = 1). The interactive online platform was pilot tested by 2 medical students and one administrative support staff member prior to the module’s launch in January 2021. All students completing their OB/GYN clerkship at our university academic medical center between January and December 2021 were required to complete the final curriculum at a part of their OB/GYN clerkship didactics. Students were invited to participate in our curricular assessment voluntarily.

### Step 6: Assessment

Students who completed the curriculum were invited to participate in pre- and post-assessment surveys. Questions included an assessment of students’ past educational experiences and confidence in caring for TGD patients, assessed on a 4-point Likert scale (strongly disagree, disagree, agree, strongly agree) (Appendix C). Students were also asked to complete a 10-question, multiple choice clinical knowledge exam before and after completing the module (total score = 15 points) (Appendix D). Topics covered included cancer screening guidelines, puberty blocking therapy, feminizing hormone therapy, masculinizing hormone therapy, health insurance, gender-affirming surgery and fertility. Students were also given the opportunity to provide qualitative feedback in a free-response box at the end of the post-assessment survey. For academic credit, students were graded on a pass/fail scale, with a passing score assigned to students completing all portions of the module. Scored knowledge assessments were only used for the purpose of curricular evaluation.

To compare pre- and post-assessment surveys and knowledge exam scores, we used McNemar’s chi-squared and paired t-tests. For this present analysis, Likert scale responses were collapsed into agree (strongly agree and agree) and disagree (strongly disagree and disagree) categories. All analyses were conducted in R (version 3.1.6). Written informed consent was obtained from all participants. The University of California San Francisco Institutional Review Board reviewed and exempted this study (#20-33008, December 27th, 2020). All methods were carried out in accordance with relevant guidelines and regulations.

## Results

The curriculum was implemented in the 2021 calendar year, and 89 students completed the lecture and interactive case-based modules. Of these 89 participants, 65 (73%) completed both the pre- and post-assessment surveys. Prior to completing the module, 45% (n = 29) of respondents agreed they had received adequate teaching on caring for transgender patients. The majority (92.3%) of students agreed that they would provide care for transgender patients in their specialty of interest. On average, students spent 77 min completing the module.

Following completion of the module, students reported increased comfort in caring for transgender patients (49.2% vs. 81.5%; p < .001). Students also endorsed an improved fund of knowledge of (1) healthcare maintenance for transgender patients (61.5% vs. 100%; p < .001), and (2) gender affirming medical therapies (60.0% vs. 96.9%; p < .001) (Table 1). Following module completion, participants’ knowledge assessment scores increased from a mean (SD) of 9.65 (1.81) to 12.5 (2.20) out of 15 (p < .001) (Table 2). In post-assessment surveys, 95% of participants agreed that the module was helpful for their learning.

**Table 1:** Students’ agreement with statements regarding their preparation to care for TGD patients (n = 65)

Statements	Pre-module n (%)	Post-module n (%)	p-value
I feel comfortable in my ability to care for transgender patients.	32 (49.2%)	53 (81.5%)	< 0.001
I have a basic fund of knowledge of health care maintenance for transgender patients.	40 (61.5%)	65 (100%)	< 0.001
I have a basic fund of knowledge of gender affirmation therapy.	39 (60.0%)	63 (96.9%)	< 0.001
I will provide care for transgender patients in my specialty of interest.	60 (92.3%)	63 (96.9%)	0.248

**Table 2:** Proportion of correct responses to knowledge exam questions pre- and post-module completion (n = 65)

Topic Assessed^a^		Pre-module n (%)	Post-module n (%)	p-value
1. Cancer screening (general principles)		52 (80.0%)	60 (92.3%)	0.027
2. Puberty blocking therapy (clinical considerations)		63 (96.9%)	62 (95.4%)	1.00
3. Puberty blocking therapy (eligibility criteria)		40 (61.5%)	46 (70.8%)	0.264
4. Feminizing hormone therapy		24 (36.9%)	42 (64.6%)	0.004
5. Masculinizing hormone therapy		4 (6.2%)	39 (60.0%)	< 0.001
6. Breast cancer screening		37 (56.9%)	48 (73.8%)	0.010
7. Health insurance		38 (58.5%)	46 (70.8%)	0.136
8. Informed consent		30 (46.2%)	50 (76.9%)	0.001
9. Gender affirming surgery		46 (70.8%)	59 (90.8%)	0.004
10. Fertility		20 (30.8%)	46 (70.8%)	< 0.001
**Scaled score (out of 15)** Mean (SD)	9.65 (1.81)		12.5 (2.20)	< 0.001

^*a*^ *Appendix D contains the full knowledge exam*

In free response comments from the post-assessment survey, students discussed the benefits of completing the module. A representative comment from one student read: “This has taught me so much valuable information regarding providing inclusive care for our patients who identify as transgender.” Students also suggested next steps to further improve TGD-care curricula at our institution more broadly, including integrating TGD-topics into pre-clinical curriculum. Participants suggested providing examples of specific language that providers could use to discuss TGD-topics and creating an additional peer-based learning session to pair with the existing content to allow time for students to review concepts and practice patient counselling

## Discussion

In partnership with patients, students, and faculty, we created an interactive curriculum on providing gender-inclusive and gender-affirming care to TGD patients for undergraduate medical students completing their OB/GYN clerkship. Preliminary evidence from our pilot study suggests that students’ knowledge of TGD health topics increased significantly following completion of our curriculum, as did their confidence in their ability to care for TGD patients. Prior to completing the module, more than half of our participants felt that they had not received adequate training in TGD care topics, despite 9 out of 10 believing that they would care for TGD patients in the future. These preliminary results suggest that our clinically focused curriculum filled an important gap in the training of undergraduate medical students in caring for TGD individuals.

Several strengths of our design contributed to the initial success of this curriculum. First, the content was delivered in multiple learning formats, including traditional didactic lectures, interactive cases, and quiz-based elements, increasing accessibility for students with various learning preferences. The interactive cases and knowledge assessments also allowed for students to actively engage with the material, reinforcing their knowledge acquisition [24, 26]. Additionally, the asynchronous design of the module allowing learners across multiple clinical sites to engage with the curriculum, despite the time-constraints imposed by each student’s unique clinical schedule. Using a digital-platform to deliver the content also allowed us to provide students with real-time feedback as they progressed through the case-based module, highlighting key-learning points throughout each case.

Our approach to developing the content of our curriculum was intentional in involving key contributors, including early partnership with transgender students, faculty, and community members. Focus groups including transgender and cisgender allied medical students, as well as early discussions with faculty with clinical expertise in caring for TGD individuals, identified key areas to be covered in our module. Most importantly, our early needs assessment work highlighted the importance of reframing content to center gender-affirming models of care [13]. Various experts were also involved in multiple rounds of review of the curricular materials prior to implementation – including faculty members, senior medical students, and patients. Early and ongoing partnerships with a broad group of contributors facilitated the intentional and systematic development of the content contained within the final curriculum.

Although there were many strengths of this study, a few limitations impacted our results. First, this is a single institution study, and thus this work would need to be disseminated to other institutions to assess reproducibility. Second, our study design only allowed for assessment of knowledge acquisition immediately after students had completed the module. Longitudinal assessment of clinical knowledge should be conducted to determine the efficacy of the module on knowledge retention. As only 73% of students completed both the pre- and post-module surveys, response bias could have contributed to the results obtained; students who were already motivated to engage in curricula on the healthcare needs TGD individuals may have been more likely to complete all curricular components, including the scored knowledge exams.

In the second iteration of this curriculum, we plan to add additional elements to expand students’ clinical training and knowledge base. In qualitative comments, various students suggested that a synchronous, in-person skills session or patient encounter would be valuable, allowing students to practice the implementation of their newly developed knowledge and clinical skills. Future implementation of this curriculum will include additional opportunities for students to gain exposure to and practice of caring for TGD patients in the clinical settings following completion of this module. This may be in the form of a peer-based small group with opportunities for role play, simulated patient encounters, 27]. or rotating through clinics that serve transgender patients [28.

Additionally, we are revising the longitudinal OB/GYN curriculum at our institution to integrate TGD health topics across medical students’ educational training. At our institution, efforts to integrate TGD care into our broader curriculum include: (1) reviewing and updating our course textbooks, workbooks, and lecture slides to use gender-inclusive language, (2) including transgender and non-binary patients in our teaching cases, and (3) highlighting special considerations for TGD patients in our course materials. Integrating TGD topics into our curriculum will reinforce concepts longitudinally, and center TGD care as a core clinical competency for students. Efforts to create standardized measures to evaluate the inclusion of LGBTQI + topics in clinical training curricula reflect an increasing recognition of the importance of integrating content across students’ professional education [29]. Ongoing assessment of our institution’s curriculum will be necessary to ensure that students graduate with competency in caring for TGD patients.

Developing a competent workforce of healthcare providers equipped to care for TGD patients requires specific and dedicated curricula on transgender health. Our institution’s experience creating and implementing a curriculum for OB/GYN clerkship students provides a template for other institutions looking to expand content related to TGD care in their clinical training programs. Structured as an asynchronous module, our curriculum was explicitly designed to facilitate easy adaptation and implementation across other institutions. However, implementing new content in the form of an adjunctive module is only a first step towards creating gender-inclusive curriculum. Integrating TGD care topics longitudinally, and centering gender-affirmation across students’ entire medical education is necessary. As other institutions seek to restructure and expand their curricula, early partnership between patients, students and faculty is central to ensuring that curricula reflect the needs of TGD patients.

## Conclusion

Our curriculum was successful in increasing undergraduate medical students’ knowledge of TGD health topics and confidence in their ability to provide gender-affirming care to their future patients. Integration of this work across other institutions is an important step towards building a competent physician workforce and increasing the accessibility of gender-inclusive care for all patients.

## Electronic supplementary material

Below is the link to the electronic supplementary material.

Supplementary material 1: Appendix A

Supplementary material 2: Appendix B

Supplementary material 3: Appendix C

Supplementary material 4: Appendix D

## Data Availability

The datasets used and/or analyzed during the current study are available from the corresponding author on reasonable request.

## List of abbreviations

TGD: Transgender and gender diverse
OB/GYN: Obstetrics and Gynecology
