# Supplementary material for: Care across the gender spectrum: A transgender health curriculum in the Obstetrics and Gynecology clerkship
Source: BMC Med Educ. 2022 Oct 5;22:706. doi: 10.1186/s12909-022-03766-0 (PMC9535842; doi:10.1186/s12909-022-03766-0)
Supplement: Supplementary file 1 — Supplementary material 1: Appendix A [file 12909_2022_3766_MOESM1_ESM.docx]

**Appendix B: Small group cases**

*Note: these cases were administered on Qualtrics, allowing students to answer questions and for dynamic teaching/learning points populate as students progressed through the module*

**Self-Reflection:**

Reflect on the following scenarios. How would you respond to the situation presented? Please respond to each prompt with 3-4 sentences.

**1) You are a practitioner in your gynecology clinic. Your next patient is a transgender man. The MA tells you that he is very upset because the front staff asked him when his wife was arriving for her appointment.**

*Answer:*

*Possible responses:*

- *Immediately address the situation with the patient: create space for the patient to talk about what happened, validate his feelings and apologize on behalf of the entire clinic, assure the patient that you will follow-up with the front staff member directly as well as hold a meeting or other form of training to prevent this from happening again*
- *Recognize that this situation could prevent the patient from returning to your clinic, creating a barrier for him to get adequate care*
- *Update patient intake forms to include pronouns and gender identity*
- *Provide adequate training for* ***all*** *clinic staff around gender diversity of gynecologic patients, stressing the importance of avoiding assumptions*
- *Consider more gender inclusive signs in the clinic (e.g., might avoid “Women’s Health” signage)*

**2) You are a medical student rotating in a teen clinic. A 14yo patient, assigned female at birth, confides in the attending that their body does not “feel like a girl’s body.” The attending responds that “you just feel that way because you do not have an hourglass figure.”**

*Answer:*

*Possible responses:*

- *Ask the patient if they are comfortable saying more about feeling like they are not in a girl’s body*
- *Provide the patient with reassurance that many people do not identify with the genders assigned to them at birth*
- *Ask the patient if they have talked about this with anyone else (parents, friends, teachers) and ask if they have ideas about where they might get support*
- *Offer patient education resources about gender identity that are age appropriate*
- *Follow-up with the attending about more appropriate responses to that situation*
- *Reach out to the clerkship director or medical school deans to support the student in addressing these concerns*
- *Consider reporting the attending’s inappropriate comment*

**Case 1: Trevor**

Trevor is a 6-year-old who was assigned male at birth and currently uses he/him/his pronouns. Since he was a toddler, he has frequently worn dresses at home with his sisters and insists on playing the little sister or the mother during make-believe games. His favorite game is playing tea party with his Barbie dolls.

Trevor’s parents recently attended a parent-teacher conference after Trevor was forced to use the boys’ bathroom at school and told all the boys that he was scared of their penises. When asked about his own penis, he says it “will shrink by the time I’m 10.”

He is often singled out and picked on to the point that he comes home in tears.

**What sex was Trevor assigned at birth?**

A) Male

B) Female

C) Intersex

*Answer: A*

**What is Trevor’s gender identity?**
A) Cis-male

B) Trans-female

C) Non-binary

D) Gender non-conforming

E) Ask the patient

*Answer: E*

*A 6-year-old may not have the language to describe their gender identity, but providers might label Trevor as non-binary or gender non-conforming.*

**As a health care provider, what resources can you offer to Trevor’s parents to address the situation at school?**

A) Consider a referral to [Lambda Legal](https://www.lambdalegal.org/), a defense and education fund for LGBT people and those living with HIV.

B) Connect them with online resources: such as: [Human Rights Campaign: Parents for Transgender Equality Council](https://www.hrc.org/resources/parents-for-transgender-equality-national-council) And [Gender Spectrum](https://www.genderspectrum.org/)

C) All of the above

*Answer: C*

*As a health care provider, you can offer multiple resources to Trevor’s parents. A few things that you can do to support Trevor’s parents include:*

- *Offer to write a letter of support the Trevor’s school as his healthcare provider*
- *Consider a referral to* [*Lambda Legal*](https://www.lambdalegal.org/)*, a defense and education fund for LGBT people and those living with HIV.*
- *Connect them with online resources:*
  - [*Human Rights Campaign: Parents for Transgender Equality Council*](https://www.hrc.org/resources/parents-for-transgender-equality-national-council)
  - [*Gender Spectrum*](https://www.genderspectrum.org/)
  - *Additional resources are listed at the end of this module*

*It’s important to know the current legal protections in place in your state. In August 2020, federal courts in Florida and Virginia ruled that students should have access to the bathrooms that align with their gender identity.*

**What are the possible negative outcomes of continued gender misidentification? *Please respond with 3-4 sentences.***

*Discrimination (including harassment, gender misidentification, etc.) contributes to negative health outcomes among transgender people, including disproportionate rates of depression, anxiety, substance use, suicidal ideation and attempts. Among school-aged transgender people, absenteeism may be a coping mechanism if they are being harassed at school. Other coping mechanisms may include bathroom avoidance, thereby increasing the risk of developing bladder/kidney infections.*

*Note: This is not an exhaustive list of negative consequences of gender misidentification; this is simply an introduction to some of the problems your future patients may face. We also do not want to trivialize the experiences of transgender and gender diverse people.*

*Source: Cicero, et al: Supporting the Health and Well-Being of Transgender Students*

**Case 2: ShawnA**

Shawna is a 12-year-old who was assigned male at birth and given the name “Shawn” by her parents. Two years ago, she requested the use of “she” pronouns and wishes to be called “Shawna.”

Shawna is increasingly frustrated by the changes in her physical body, which are manifesting in stereotypically male attributes. On physical exam, she exhibits a testicular volume of 8mL, penile length of 3cm, and coarse pubic hair that begins to curl.

Shawna’s parents support her affirmed gender.

**What Tanner stage would you classify Shawna?**

A) Tanner 1

B) Tanner 2

C) Tanner 3

D) Tanner 4

*Answer: B*

**Does Shawna meet eligibility criteria for medical suppression of puberty?**
A) Yes

B) No

C) Not clear

*Answer: A*

**What medical management would you provide to suppress puberty?**

A) GnRH analogues

B) Spironolactone

C) Amiodarone

*Answer: A*

*GnRH analogues decrease the growth of facial and body hair, prevent voice deepening, and the further development of genitalia in those assigned male at birth. Medications should be started prior to completion of puberty, generally before age of 16. GnRH analogues do not cause permanent change; they pause puberty, allowing patients more time to explore their gender identity and decide if they would like to pursue other gender affirming treatments. It is also important to consider the costs and feasibility of taking medications with the patient and family to help minimize barriers to Shawna’s gender affirmation.*

*In order for Shawna to have biological children in the future, these are some of the considerations: Puberty blockers pause pubertal development, preventing a younger person’s endogenous gonadal hormones from continuing physical changes in their body. Currently, most fertility preservation interventions cannot occur prior to Tanner Stage 4, when most eggs and sperm are mature enough for fertility.*

**What name and pronouns should you use in Shawna’s medical record?**

*Answer: Shawna. She/her. Be sure to ask patient about her gender identity; medical record should include this.*

**What name and gender should you use with Shawna’s insurance?**

*Answer: Shawn. Male.*

*Health insurance companies in the U.S. use legal name and assigned sex at birth, and do not have room for lived names or non-binary identities.*

*Note: While the national LGBTQ task force has acknowledged that the term “gender dysphoria” may be used as a temporary stopgap measure for health insurance & medical reimbursement purposes, gender variance is not a mental health disorder*

**What would be the next steps in management of care if Shawna’s parents did not support her affirmed gender? *Please respond with 3-4 sentences.***

*Answer: Regardless of parental support, you should continue to follow Shawna's lead in addressing her by her preferred name and pronouns****.*** *Her name and/or pronouns may change down-the-line as she continues to have space to explore her gender. Check in with her regularly to be sure you use the correct name and pronouns, as well as what name/pronouns she would like you to use in front of her parents.*

*Other possible steps to take include:*

- *Explain the negative long-term consequences of non-affirming behavior and harmful interventions to Shawna’s parents. Research suggests that family support and a sense of a positive future are resilience factors that protect against negative health and psychosocial outcomes for transgender and gender diverse people (*[*Facts About “Conversion Therapy,”*](https://www.apadivisions.org/division-44/resources/conversion-fact-sheet.pdf) *Society for the Psychology of Sexual Orientation and Gender Diversity, American Psychological Association)*
- *Know the minor/parent consent laws where you practice*
- *Provide Shawna’s parents with resources about how to support transgender, non-binary, and gender fluid children. A few examples include:*
  - [*Human Rights Campaign: Parents for Transgender Equality Council*](https://www.hrc.org/resources/parents-for-transgender-equality-national-council)
  - [*PFLAG: Our Trans Loved Ones: Questions and Answers for Parents, Family, Friends of People Who are Transgender and Gender Expansive*](https://pflag.org/sites/default/files/Our%20Trans%20Loved%20Ones.pdf)
- *Provide Shawna with resources. A few examples include:*
  - [*Trans Youth Equality Foundation*](http://www.transyouthequality.org/for-youth-1)*. Transgender Youth Retreat, legal resources, information about gender-affirming care. Also includes resources for parents & clinicians.*
  - [*UCSF Center for Excellence in Transgender Health*](https://transcare.ucsf.edu/welcome-0)*. Site also includes resources for clinicians*
  - *Unfortunately, transgender youth experience disproportionate rates of homelessness in the United States.* [*Larkin Street Youth Services*](https://transcare.ucsf.edu/welcome-0) *in San Francisco strives to help those experiencing housing insecurity in housing, medical care, education, and employment. Consult the clinic’s social worker, if available and if the patient interested.*
  - [*TrevorLifeline*](https://www.thetrevorproject.org/get-help-now/)*. A 24/7 crisis counselors for LGBTQ youth, 866-488-7386. Texting and online chat services are also provided.*

**Case 3: Jae**

Jae is a 21-year-old person assigned female at birth who identifies as genderqueer and uses they/them pronouns. They do not identify as a man or a woman.

They have been chest binding for over 3 years and are interested in top surgery. They are not interested in hormonal treatments and may want to be pregnant in the future. They are sexually active and have not seen a doctor in 5 years.

**What gender and pronouns should you use in Jae’s medical records?**

*Answer: Genderqueer, they/them*

**What gender should you reference when billing insurance for the visit?**

*Answer: Female (due to limitations with U.S. health insurance companies, see the answer key for case 2)*

**What are the next steps in management for Jae? *Please respond with 3-4 sentences.***

*Answer: Plastic surgery referral for top surgery. Consider referring patient to UCSF Transgender Care, with team members who specialize in plastic surgery, gynecology & gynecologic surgery, sexual health, ovarian fertility, social work, and more. Discuss a referral to mental health provider (therapist and/or psychiatrist), ideally referring to a provider with experience working with gender diverse patients; some insurers and surgeons require letters from mental health providers attesting that patients have been living in their affirmed gender for >1 year in order to approve surgery. Additionally, supportive psychotherapy and medical gender affirmation treatment have been associated with better mental health outcomes.*

*Note: Many gender affirming surgeons in San Francisco use the affirmative consent model where no such letters from mental health providers are necessary.*

**What other issues would you want to talk to Jae about at this visit? *Please respond with 3-4 sentences.***

*Answer: Discuss methods to prevent STIs and pregnancy if not currently desired. It is important to ask genders of partners and how they engage in sex (body parts used) for adequate counseling and screening. Jae may benefit from PrEP. Consider an Ob/Gyn referral, ideally to a gender inclusive clinic.*

*Note: Do not assume that a patient desiring top surgery will also desire hormone therapy and/or bottom surgery.*

*Resource: “Communicating With Patients Who Have Nonbinary Gender Identities” from The Annals of Family Medicine.* [*https://www.annfammed.org/content/16/6/559*](https://www.annfammed.org/content/16/6/559)

**CASE 4: Adam**

Adam is a 42-year-old transgender man who is new to your practice. Today he presents to your clinic with persistent bloating and lower abdominal pain. Adam says that he has been feeling increasingly uncomfortable over the past year. At his most recent primary care visit 6 months ago, his doctor told him that he was likely constipated, and recommended dietary changes and laxatives. Since then, his symptoms have worsened, and he is now experiencing difficulties with urination. He says that he delayed coming back to the clinic because he has had “many bad experiences with the medical system.”

Adam started testosterone therapy in his 20s. He has not undergone any gender-affirming surgeries.

**What steps should your clinic take to prevent Adam from having another negative experience while seeking care? *Please respond with 2-3 sentences.***

*Answer: Use Adam’s correct name and pronouns and ensure that all clinic staff do the same. Ask how Adam refers to his body parts and use that terminology. Practice trauma-informed care when taking the history and conducting a physical exam.*

**What types of screening are indicated?**

1. CA-125 serologic testing
2. Transvaginal ultrasound
3. Pap smear, mammogram
4. All of the above

*Answer: D*

*Be sure to discuss the indications of these screenings with Adam and be flexible with his preferences, as these screenings may contribute to his gender dysphoria.*

**CASE CONTINUTED**

Adam completes a pelvic ultrasound, which demonstrates an adnexal mass with features concerning for ovarian malignancy. He undergoes surgical evaluation for ovarian cancer, and pathology confirms high-grade serious carcinoma.

The next steps in management include a discussion with a gynecologic oncologist about surgical resection of affected organs, as well as possible chemotherapy.

This case illustrates the importance to gender affirmative care in order to create an inclusive clinical setting for transgender patients so that they will not avoid preventive health care.

The need for medical practitioners to recognize the health conditions unique to each of their patients:

- Screen for conditions based on the patient's entire history - learn to conduct an inclusive history
- Understand how the patient's history indicates their risks for certain conditions (i.e. hormone therapy potentially increases risk for ovarian cancer)
- Refer transgender men to gynecological specialists (find a gender-affirming gyn clinic to recommend) as part of the preventive care team

**ADDITIONAL RESOURCES**

*While we are fortunate to have the UCSF Transgender Care program, it is important that all physicians are able to provide quality care to their transgender and gender diverse patients.*

**Resources for Nursing Professionals, School Staff, Families, Health-Care Providers, and Transgender Individuals.**

|  |  |
| --- | --- |
| Organization | Description |
| Beyond Gender Project | Resources that help explain the complicated idea of gender, information on social and medical transitioning, and information about advocacy and current issues. www.beyondgenderproject.org |
| Gender Spectrum | Resources to empower your relationships, work, and interactions with youth and children, including how-to guides, sample training materials, and tools necessary to create gender inclusive environments. www.genderspectrum.org |
| GLSEN | The leading national education organization focused on ensuring safe and affirming schools for lesbian, gay, bisexual, transgender, queer or questioning students. www.glsen.org |
| National Center for Transgender Equality | Resources on laws and policies affecting transgender people, including legal name and gender marker changes and educational materials. www.transequality.org |
| The National LGBT Health Education Center | Webinars, video training, learning modules, continuing education, and resources. www.lgbthealtheducation.org |
| Trans Lifeline | A hotline staffed by transgender people primarily for transgender people experiencing a crisis. www.translifeline.org The United States: (877) 565–8860 and Canada: (877) 330–6366 |
| UCSF Center of Excellence for Transgender Health | Guidelines for the care of transgender and gender nonbinary People. www.transhealth.ucsf.edu/protocols Learning center topics include routine care, cultural competency, mental health, policy; primary care guidelines; online training; and education. https://goo.gl/a8Dcno |
| World Professional Association of Transgender Health (WPATH) | An interdisciplinary professional and educational organization devoted to transgender health. WPATH also provides the standards of care for the health of transsexual, transgender, and gender nonconforming people. www.wpath.org |

Source: Cicero, et al: Supporting the Health and Well-Being of Transgender Students

ACOG Transgender Health Curriculum: <https://www.acog.org/education-and-events/creog/curriculum-resources/additional-curricular-resources/transgender-health-care>

Beyond Men, Women, or Both: A Comprehensive, LGBTQ-Inclusive, Implicit-Bias-Aware, Standardized-Patient-Based Sexual History Taking Curriculum: <https://www.ncbi.nlm.nih.gov/pmc/articles/PMC6338175/>

Communicating With Patients Who Have Nonbinary Gender Identities. Goldhammer, Malina, Keuroghlian. The Annals of Family Medicine November 2018. <https://www.annfammed.org/content/16/6/559>

**References**

- Bindman et al. Health Care Experiences of Patients with Nonbinary Gender Identities. Transgender Health. Published online August 17, 2021. doi:10.1089/trgh.2021.0029
- Cicero et al. "Supporting the Health and Well-Being of Transgender Students." J Sch Nurs. 2017 Apr; 33(2): 95–108.
- Goldhammer et al. Communicating With Patients Who Have Nonbinary Gender Identities. The Annals of Family Medicine Nov 2018, 16 (6) 559-562.
- Johnson, Kelly, et al. “Invalidation Experiences among Non-Binary Adolescents.” The Journal of Sex Research. 2020:57.2. pp 222-233
- Morgan, Jules. “Trans* Health: Diversity, Not Pathology.” The Lancet. 2015: 2.2. pp 124-125
- Wesp, Linda. UCSF Transgender Care. “Prostate and testicular cancer considerations in transgender women.” <https://transcare.ucsf.edu/guidelines/prostate-testicular-cancer>
- QueerDoc.com: "Puberty and Tanner Stages." <https://queerdoc.com/puberty-and-tanner-stages/>
- Refinery 29. “Gender Nation Glossary.” https://www.refinery29.com/en-us/lgbtq-definitions-gender-sexuality-terms
- Society for the Psychology of Sexual Orientation and Gender Diversity. American Psychological Association. <https://www.apadivisions.org/division-44>
- The Trevor Project.
- University of California, San Francisco. “Guidelines for the Primary and Gender-Affirming Care of Transgender and Gender Nonbinary People.” https://transcare.ucsf.edu/guidelines.
