# Supplementary material for: Care across the gender spectrum: A transgender health curriculum in the Obstetrics and Gynecology clerkship
Source: BMC Med Educ. 2022 Oct 5;22:706. doi: 10.1186/s12909-022-03766-0 (PMC9535842; doi:10.1186/s12909-022-03766-0)
Supplement: Supplementary file 2 — Supplementary material 2: Appendix B [file 12909_2022_3766_MOESM2_ESM.docx]

**Appendix C: Experience & Confidence Assessment**

**Please indicate your level of agreement with the following statements *(strongly disagree, disagree, agree, strongly agree):***

1. I feel comfortable in my ability to care for transgender patients.
2. I have a basic fund of knowledge of health care maintenance for transgender patients.
3. I have a basic fund of knowledge of gender affirmation therapy.
4. This module was helpful for my learning.
5. I will care for transgender patients in my specialty of interest.

**Post-module feedback:** Do you have any feedback on this module? Is there anything that you would like us to change?
