# Supplementary material for: Care across the gender spectrum: A transgender health curriculum in the Obstetrics and Gynecology clerkship
Source: BMC Med Educ. 2022 Oct 5;22:706. doi: 10.1186/s12909-022-03766-0 (PMC9535842; doi:10.1186/s12909-022-03766-0)
Supplement: Supplementary file 3 — Supplementary material 3: Appendix C [file 12909_2022_3766_MOESM3_ESM.docx]

**Appendix D: Knowledge Exam**

1. Cancer screening for a transgender patient should be based on:
   1. Guidelines for sex assigned at birth
   2. Guidelines for gender identity
   3. Guidelines based on present anatomy

*Answer: C*

1. Puberty blocking therapy is permanent.
   1. True
   2. False

*Answer: B*

1. What is the upper limit of eligibility for puberty suppression?
   1. 14-years-old
   2. 16-years-old
   3. Tanner stage 3
   4. Tanner state 4

*Answer: C*

1. Which of the following medications are used in feminizing hormone therapy?
   1. Spironolactone
   2. GnRH agonist
   3. Amiodarone
   4. 5-alpha-reductase inhibitors

*Answer: A, B and D*

1. The following labs/tests are used to monitor for the adverse effects of testosterone hormone therapy (select all that apply):
   1. Serum prolactin
   2. TSH
   3. 25OH Vitamin D
   4. DEXA scan

*Answer: A, C and D*

1. Transwomen >50 years old who have been on estrogen therapy for >5 years require breast cancer screening every:
   1. 1 year
   2. 2 years
   3. 5 years
   4. 10 years

*Answer: B*

1. What criteria do many U.S. insurance companies require for gender affirming surgeries? Check all that apply.
   1. 18-years or older
   2. Using gender affirming hormones for > 1 year
   3. Legally changed gender marker for > 1 year

*Answer: A and B*

1. The “Informed Consent Model”
   1. Allows adolescents to begin gender affirmation therapy without parental consent
   2. Reduces barriers for adults seeking gender affirming care
   3. Requires an assessment by a mental health provider prior to initiating gender affirming surgery

*Answer: B*

1. All of the following are transmasculine gender affirming surgeries EXCEPT:
   1. Metoidioplasty
   2. Hysterectomy
   3. Phalloplasty
   4. Orchiectomy

*Answer: D*

1. It is not possible for pre-pubescent children to preserve their gametes.
   1. True
   2. False

*Answer: A*
