# Supplementary material for: Care across the gender spectrum: A transgender health curriculum in the Obstetrics and Gynecology clerkship
Source: BMC Med Educ. 2022 Oct 5;22:706. doi: 10.1186/s12909-022-03766-0 (PMC9535842; doi:10.1186/s12909-022-03766-0)
Supplement: Supplementary file 4 — Supplementary material 4: Appendix D [file 12909_2022_3766_MOESM4_ESM.pptx]

## Slide 1
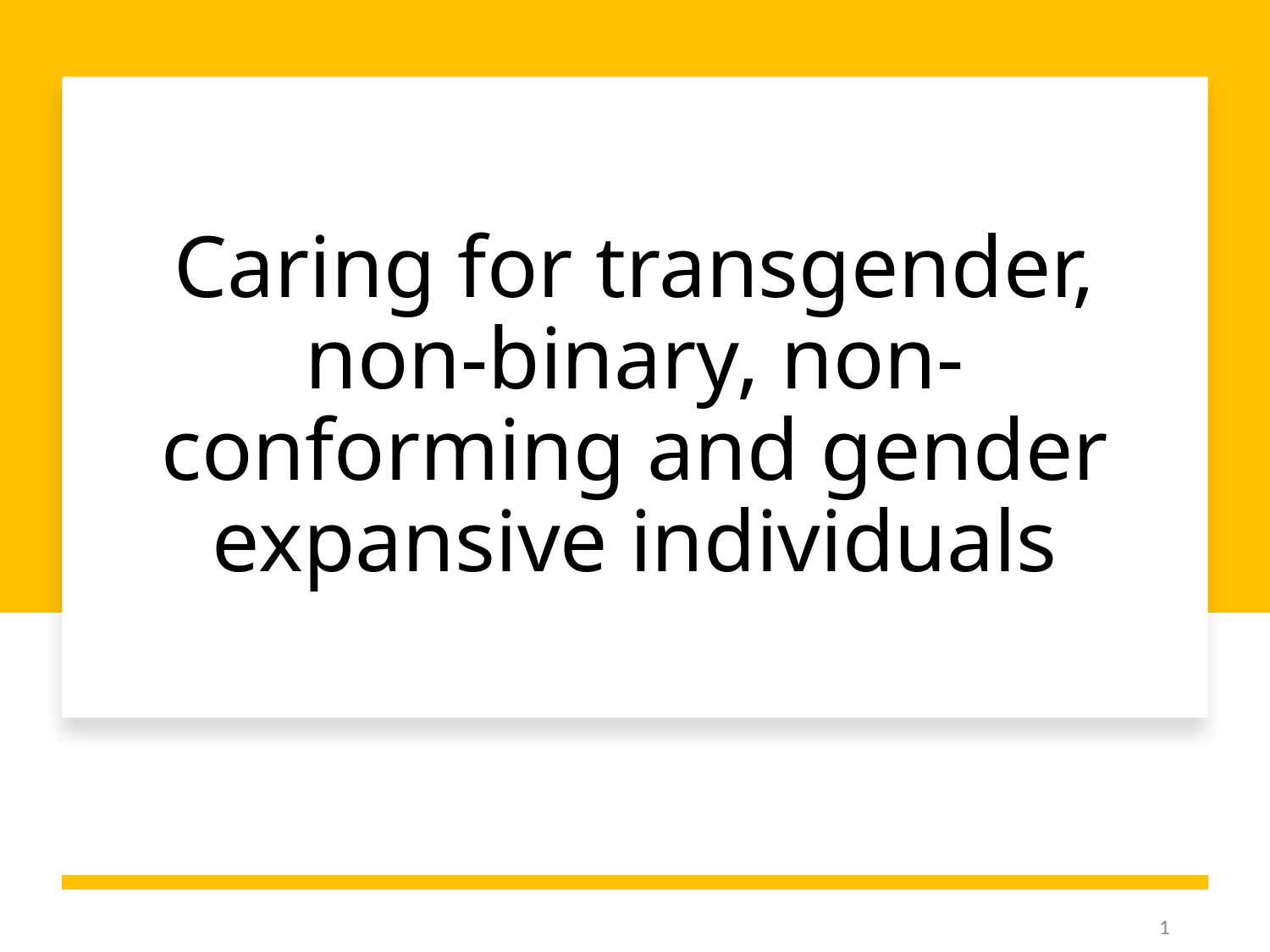

# Caring for transgender, non-binary, non-conforming and gender expansive individuals
1

## Slide 2
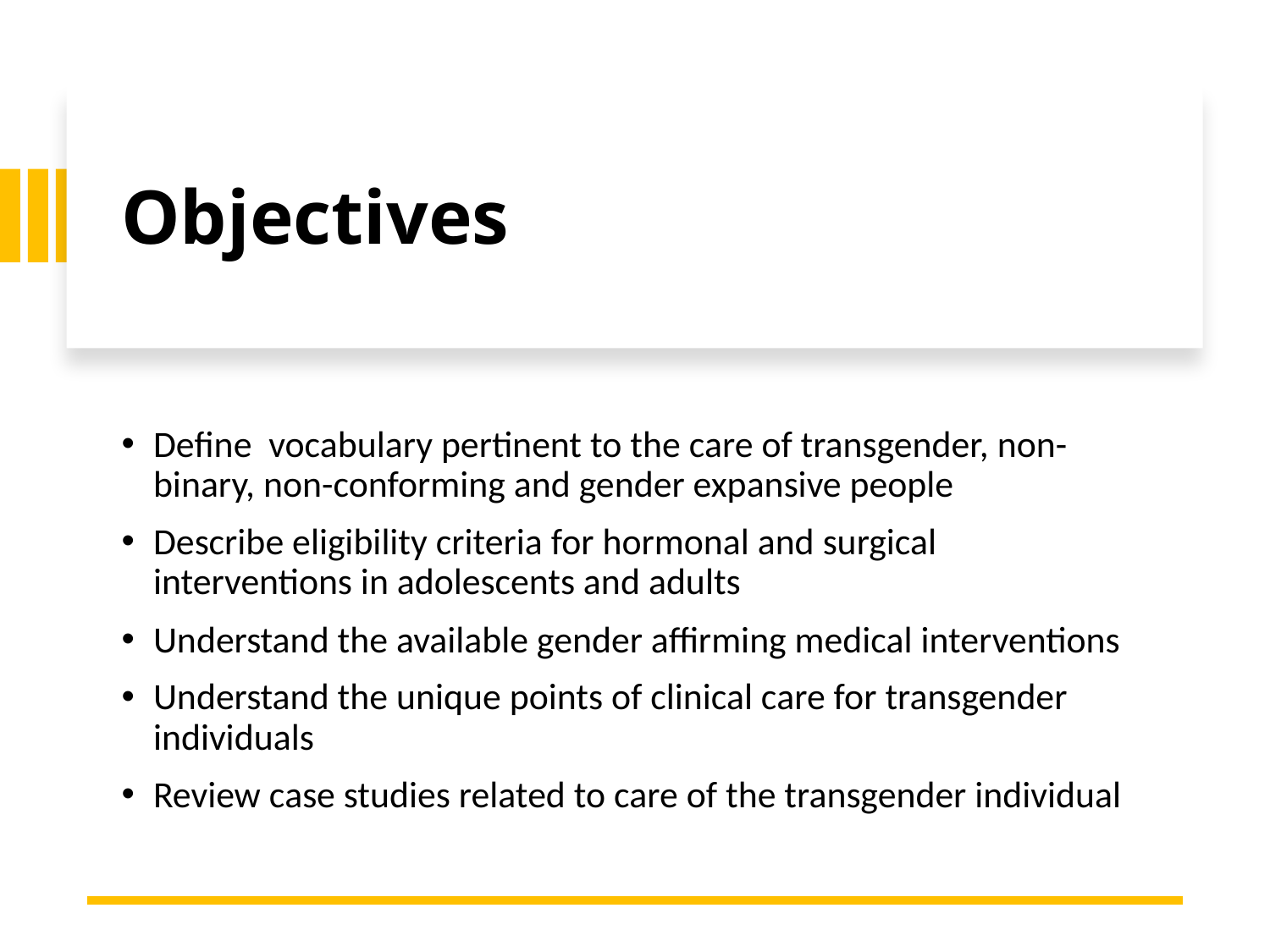

# Objectives
Define vocabulary pertinent to the care of transgender, non-binary, non-conforming and gender expansive people
Describe eligibility criteria for hormonal and surgical interventions in adolescents and adults
Understand the available gender affirming medical interventions
Understand the unique points of clinical care for transgender individuals
Review case studies related to care of the transgender individual
2

## Slide 3
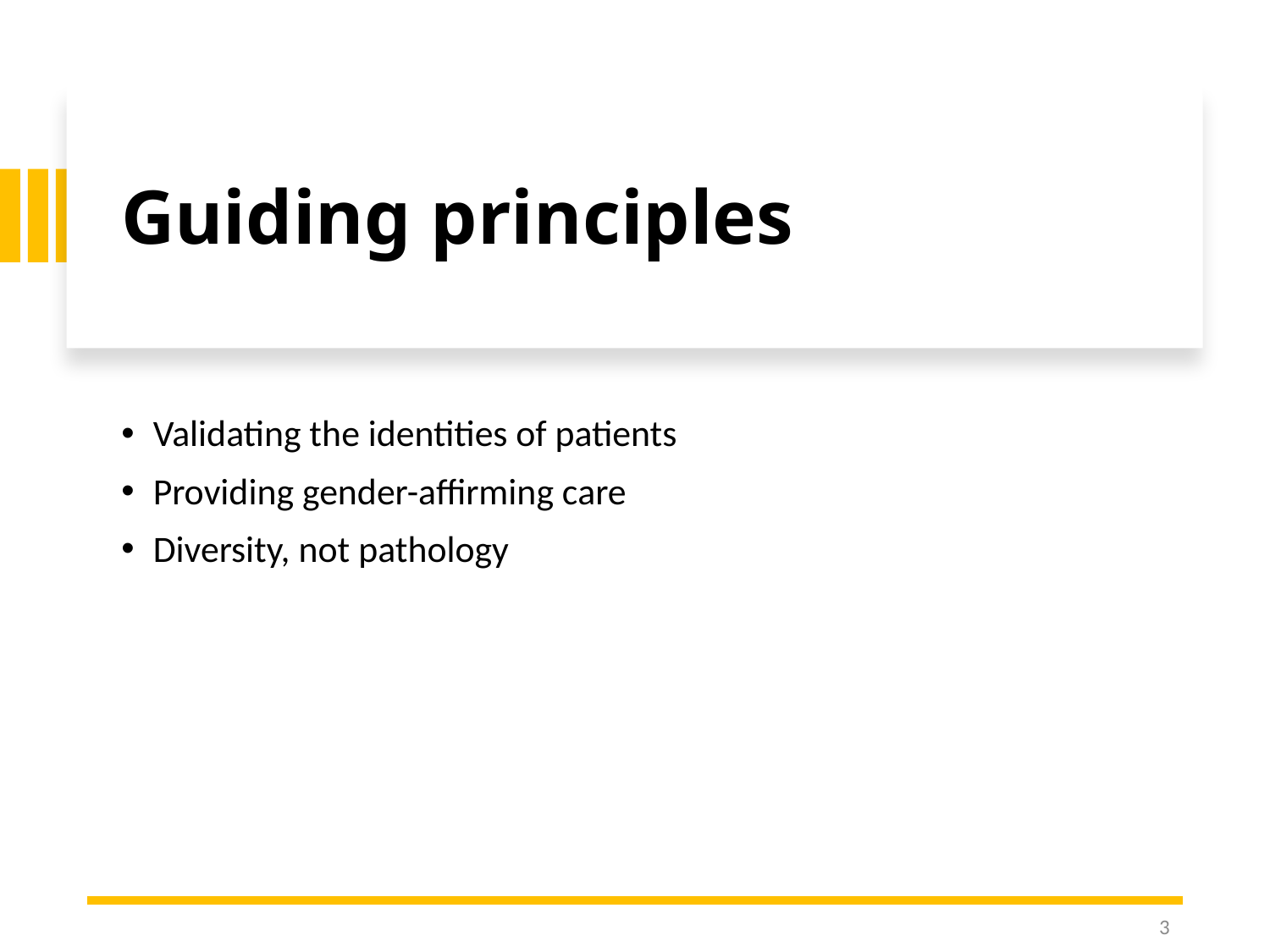

# Guiding principles
Validating the identities of patients
Providing gender-affirming care
Diversity, not pathology
3

## Slide 4
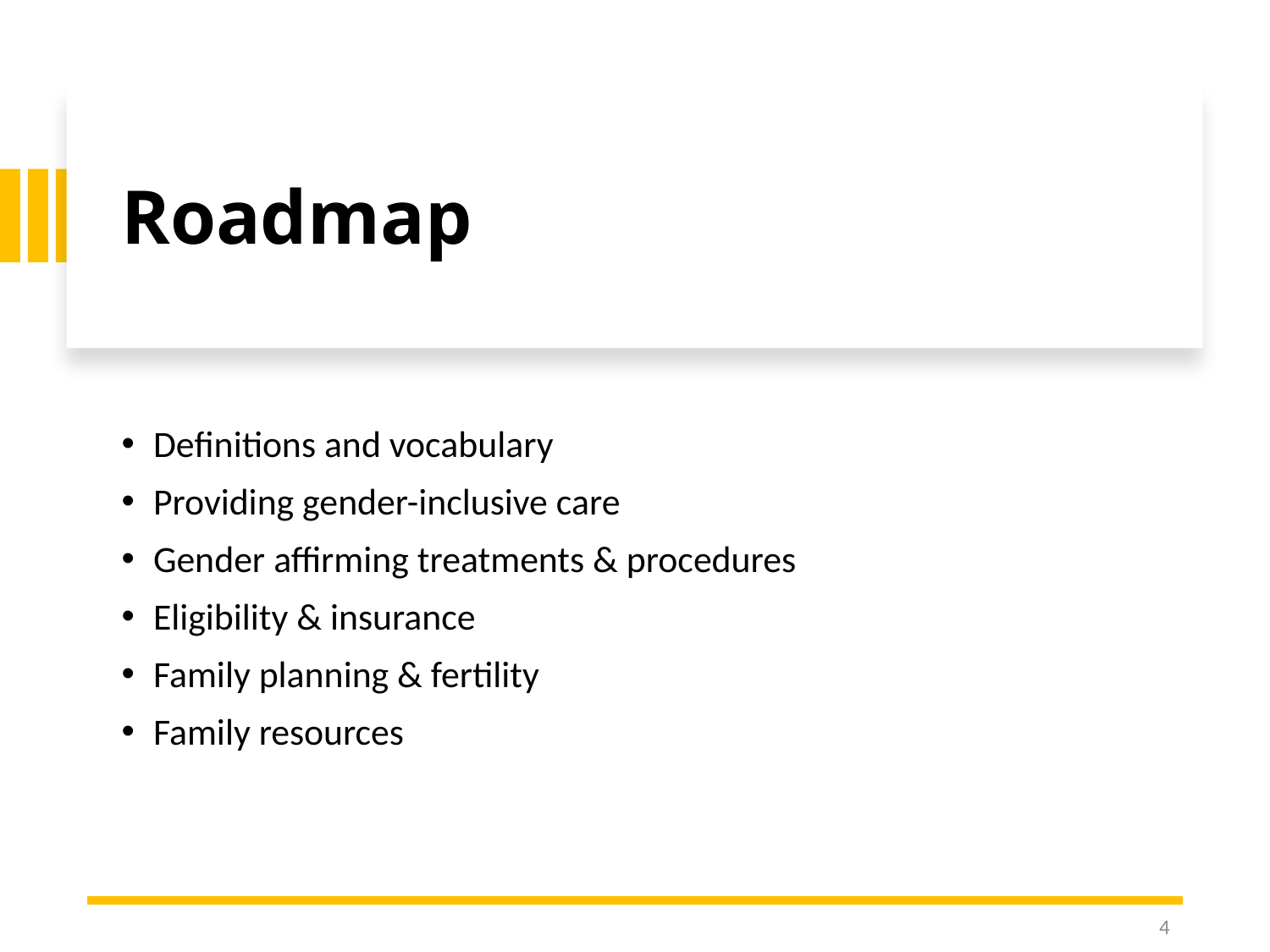

# Roadmap
Definitions and vocabulary
Providing gender-inclusive care
Gender affirming treatments & procedures
Eligibility & insurance
Family planning & fertility
Family resources
4

## Slide 5
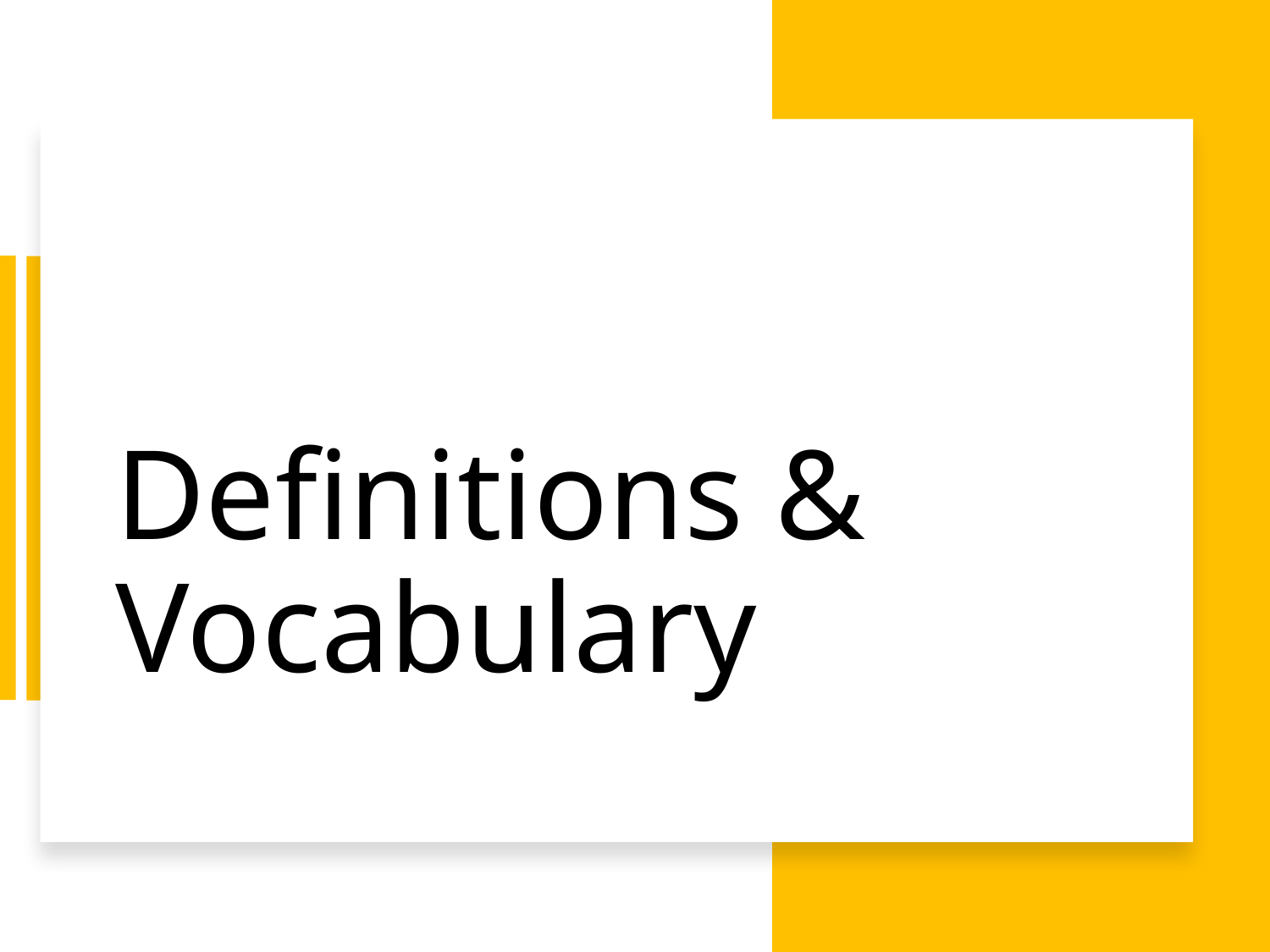

# Definitions & Vocabulary
5

## Slide 6
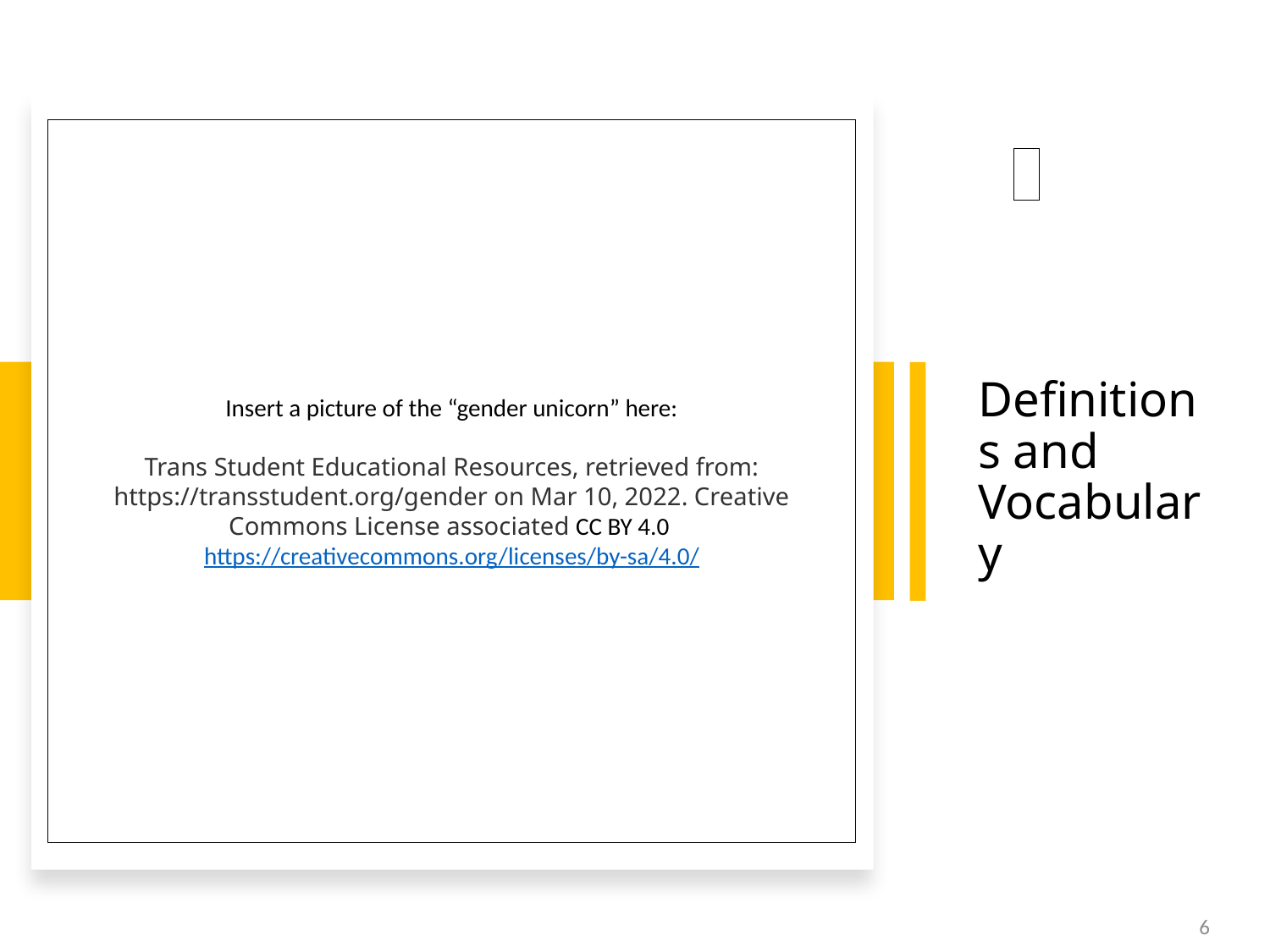

Insert a picture of the “gender unicorn” here:
Trans Student Educational Resources, retrieved from: https://transstudent.org/gender on Mar 10, 2022. Creative Commons License associated CC BY 4.0 https://creativecommons.org/licenses/by-sa/4.0/
# Definitions and Vocabulary
6

## Slide 7
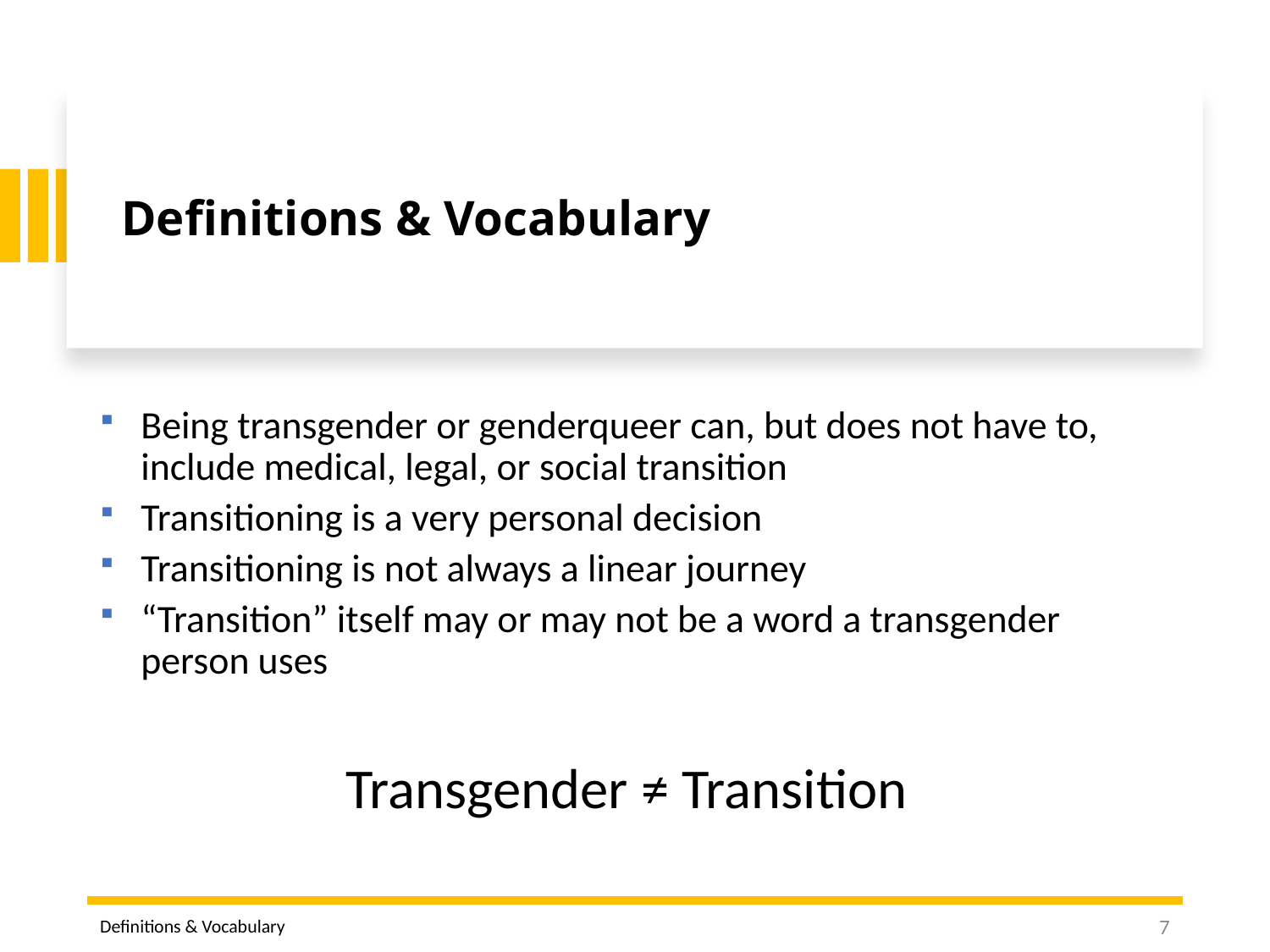

# Definitions & Vocabulary
Being transgender or genderqueer can, but does not have to, include medical, legal, or social transition
Transitioning is a very personal decision
Transitioning is not always a linear journey
“Transition” itself may or may not be a word a transgender person uses
Transgender ≠ Transition
7
Definitions & Vocabulary

## Slide 8
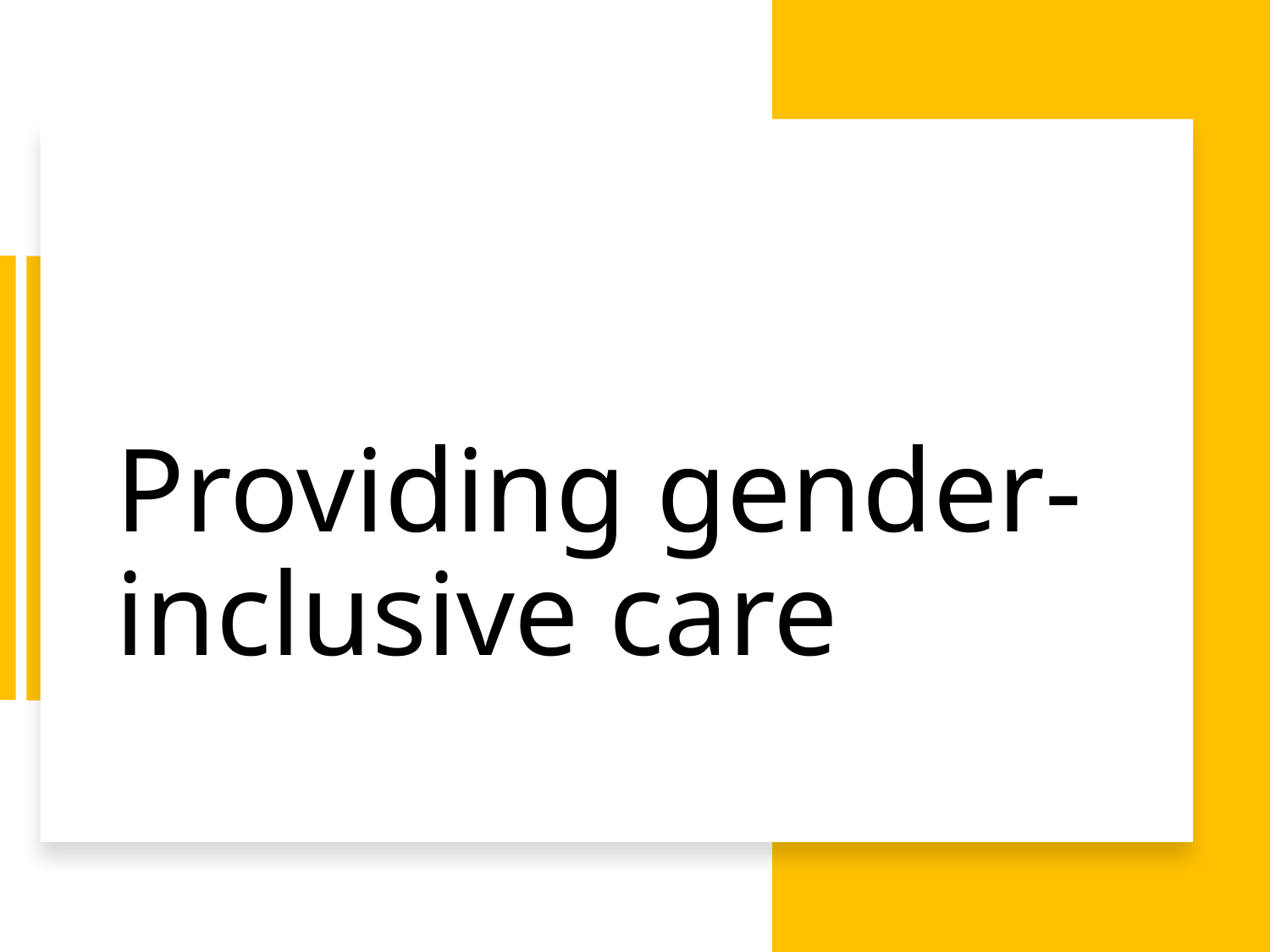

# Providing gender-inclusive care
8

## Slide 9
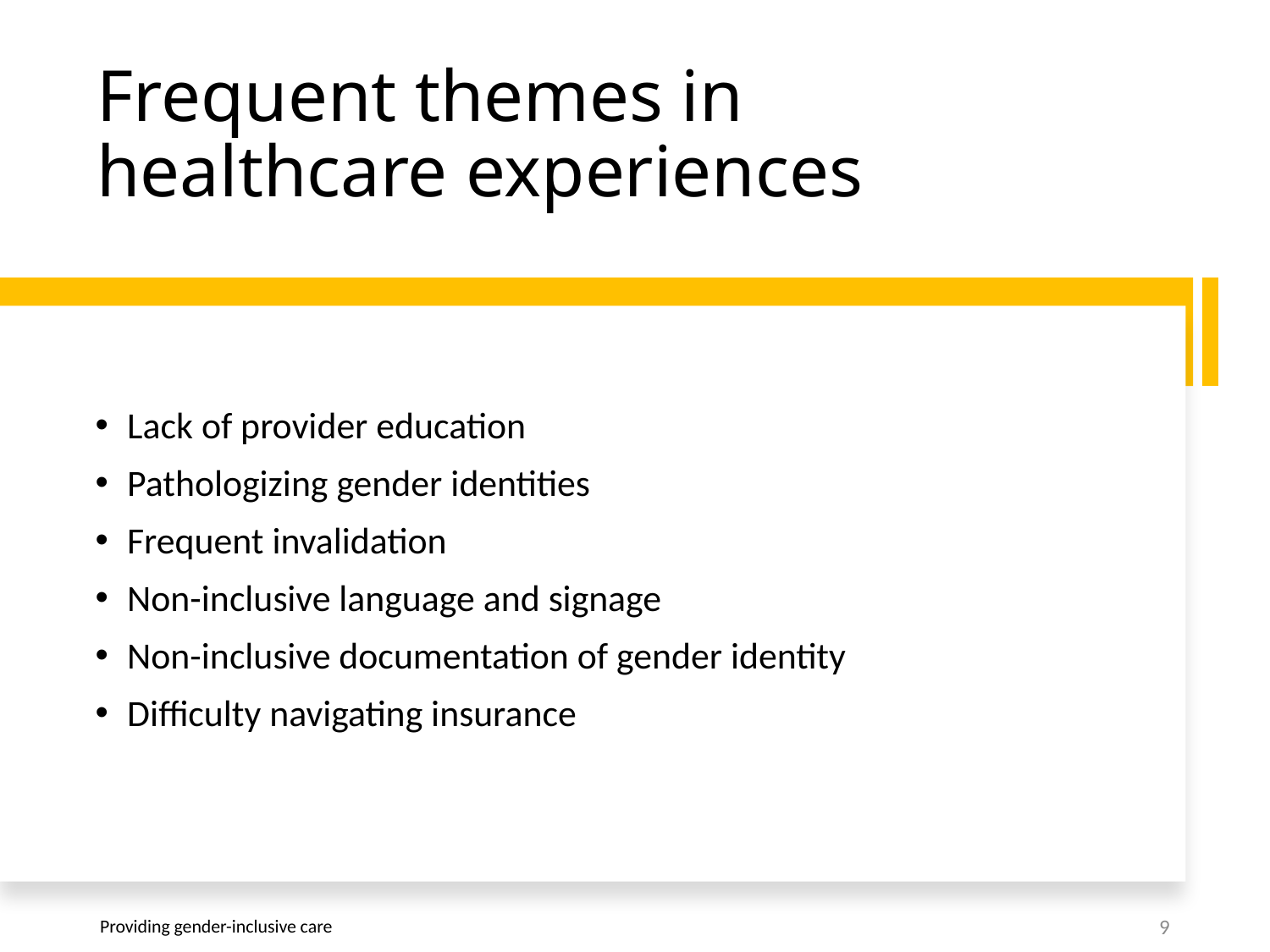

# Frequent themes in healthcare experiences
Lack of provider education
Pathologizing gender identities
Frequent invalidation
Non-inclusive language and signage
Non-inclusive documentation of gender identity
Difficulty navigating insurance
9
Providing gender-inclusive care

## Slide 10
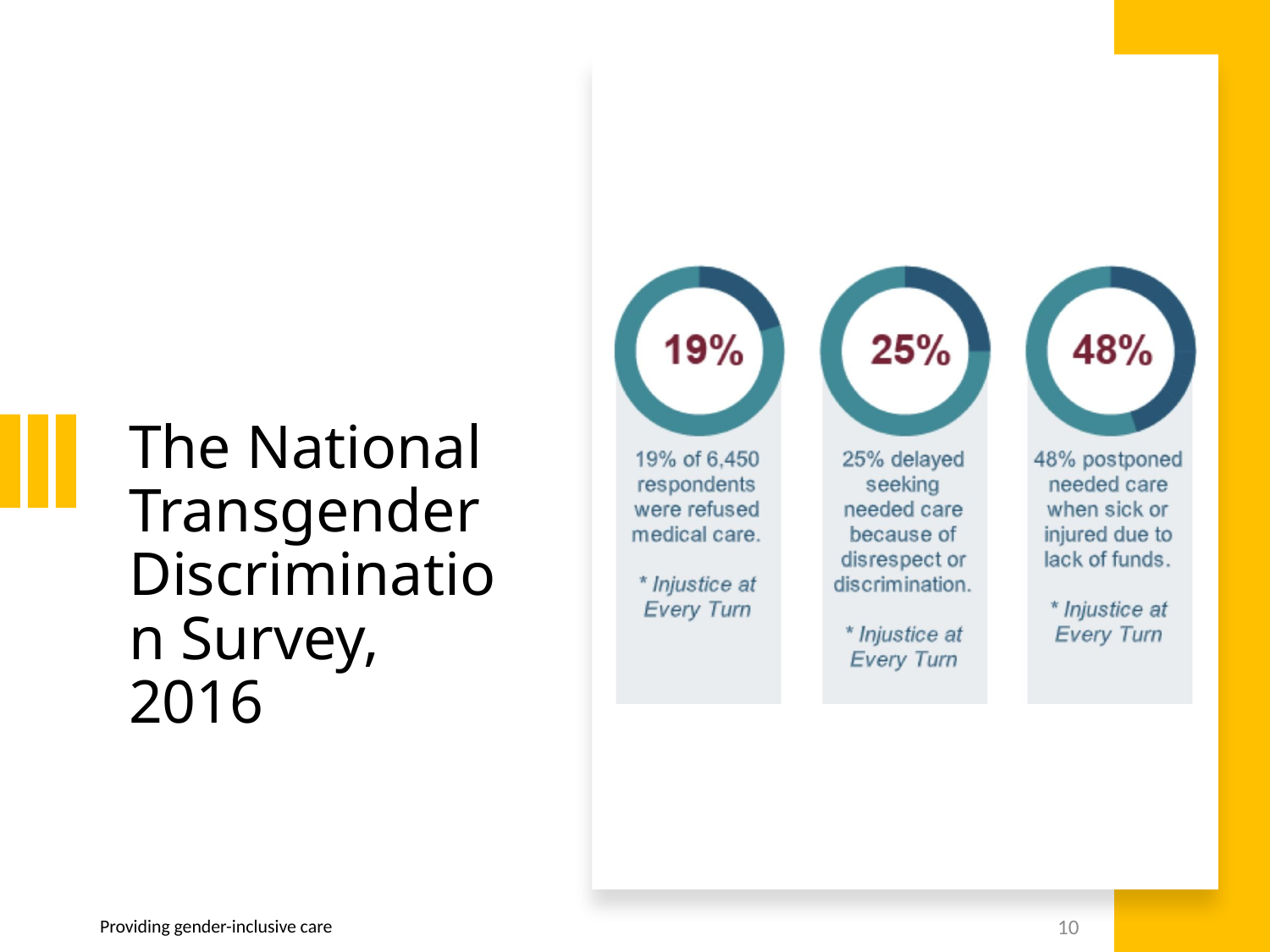

# The National Transgender Discrimination Survey, 2016
10
Providing gender-inclusive care

## Slide 11
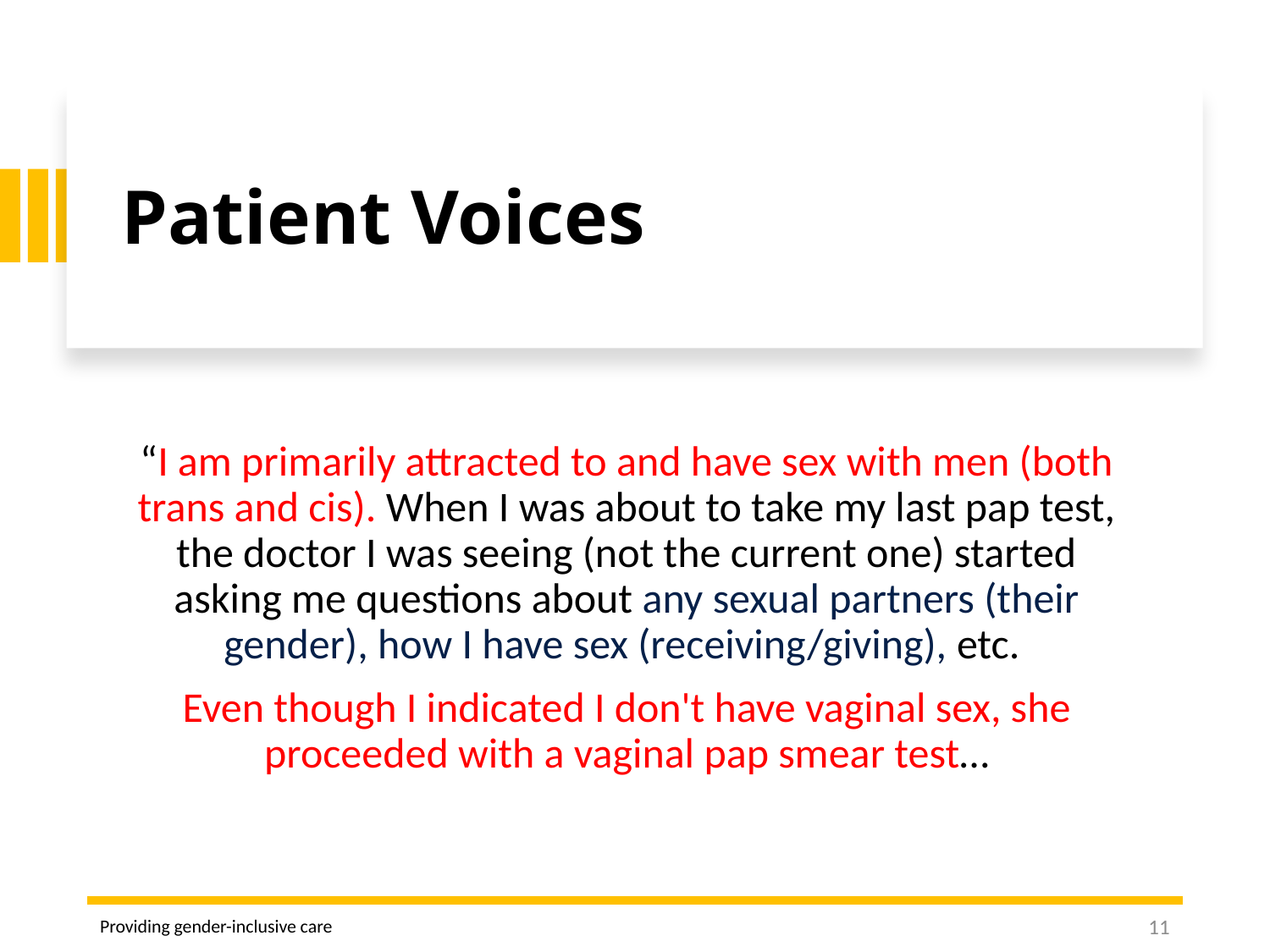

# Patient Voices
“I am primarily attracted to and have sex with men (both trans and cis). When I was about to take my last pap test, the doctor I was seeing (not the current one) started asking me questions about any sexual partners (their gender), how I have sex (receiving/giving), etc.
Even though I indicated I don't have vaginal sex, she proceeded with a vaginal pap smear test…
11
Providing gender-inclusive care

## Slide 12
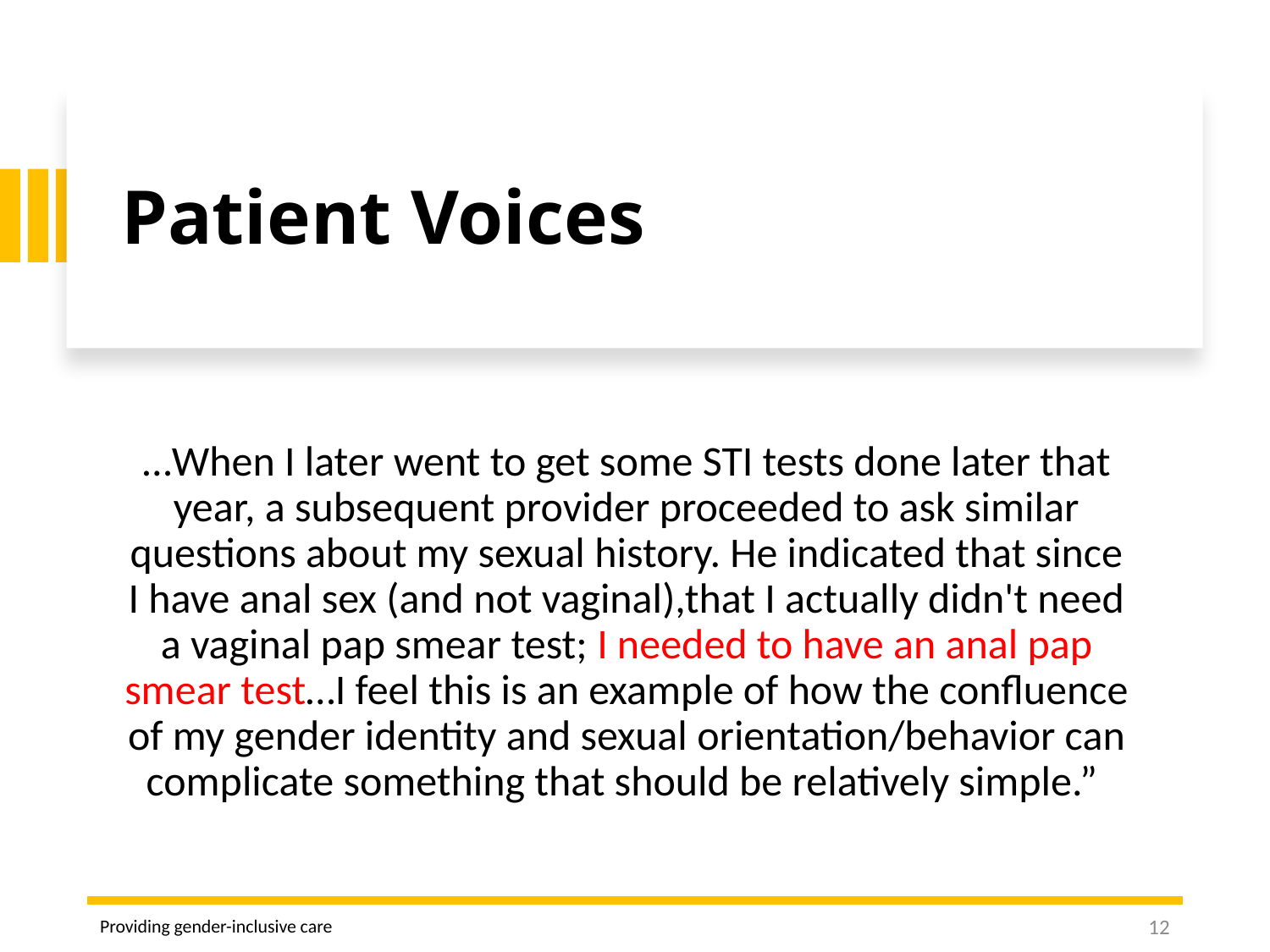

# Patient Voices
…When I later went to get some STI tests done later that year, a subsequent provider proceeded to ask similar questions about my sexual history. He indicated that since I have anal sex (and not vaginal),that I actually didn't need a vaginal pap smear test; I needed to have an anal pap smear test…I feel this is an example of how the confluence of my gender identity and sexual orientation/behavior can complicate something that should be relatively simple.”
12
Providing gender-inclusive care

## Slide 13
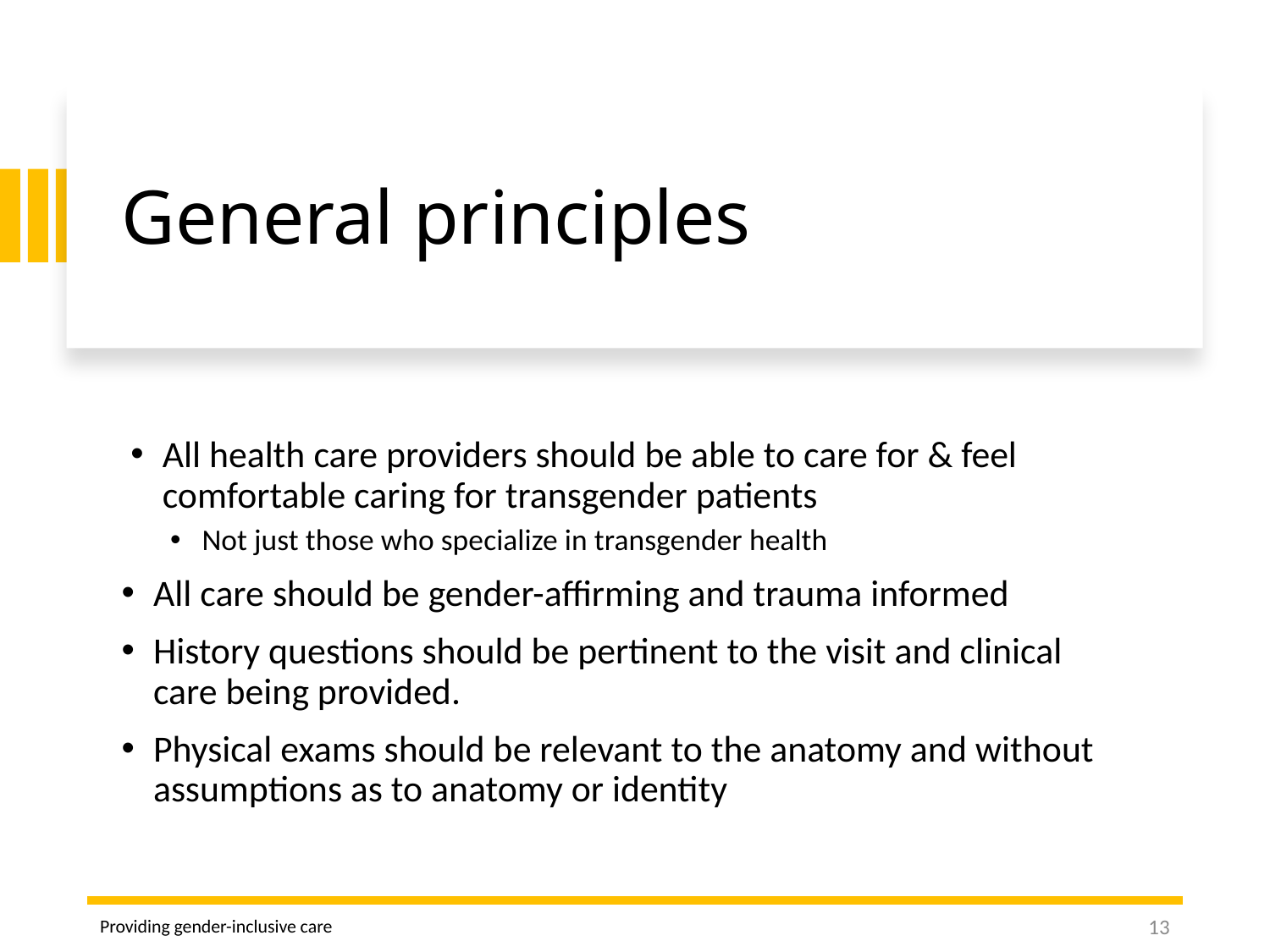

# General principles
All health care providers should be able to care for & feel comfortable caring for transgender patients
Not just those who specialize in transgender health
All care should be gender-affirming and trauma informed
History questions should be pertinent to the visit and clinical care being provided.
Physical exams should be relevant to the anatomy and without assumptions as to anatomy or identity
13
Providing gender-inclusive care

## Slide 14
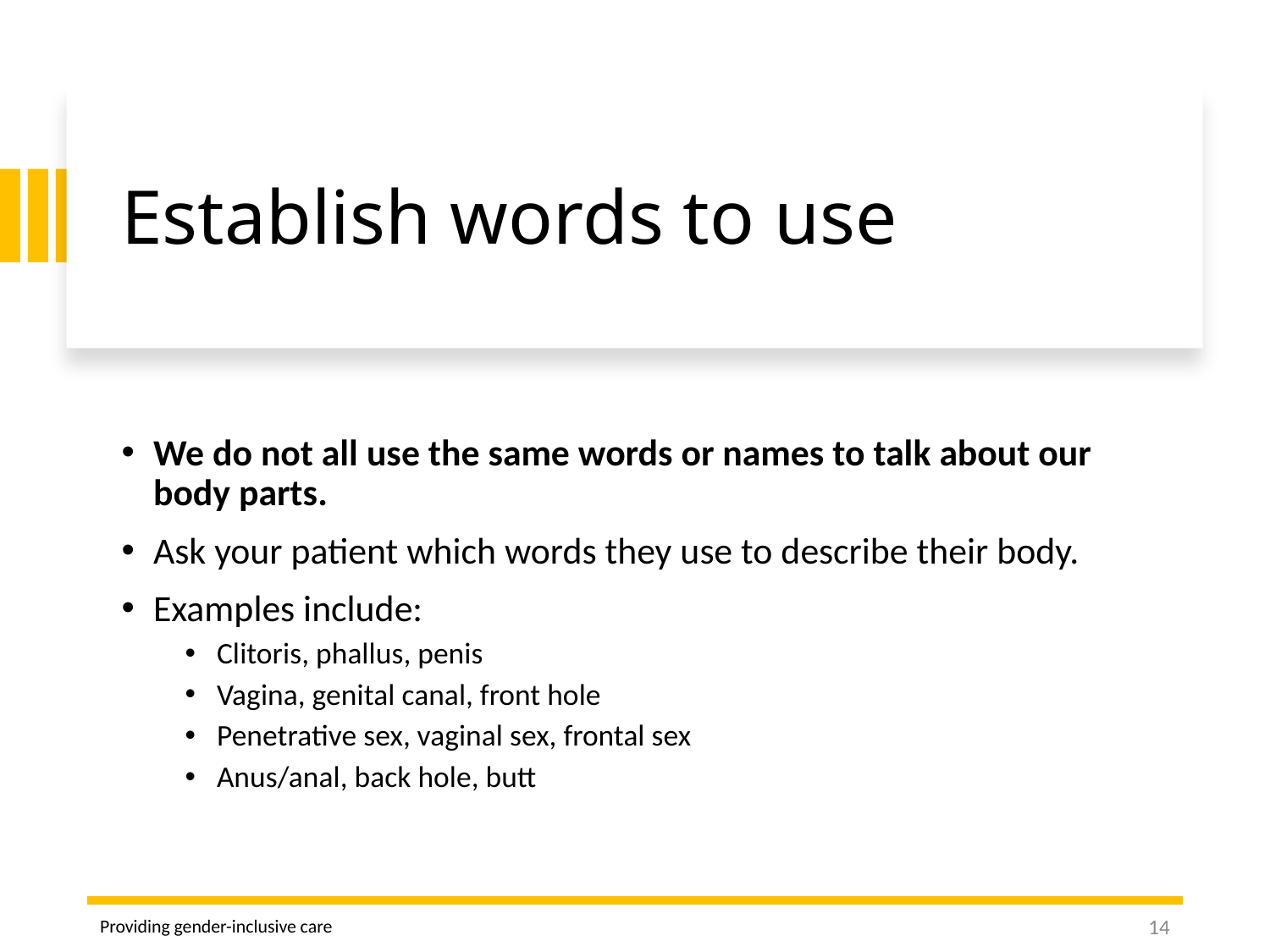

# Establish words to use
We do not all use the same words or names to talk about our body parts.
Ask your patient which words they use to describe their body.
Examples include:
Clitoris, phallus, penis
Vagina, genital canal, front hole
Penetrative sex, vaginal sex, frontal sex
Anus/anal, back hole, butt
14
Providing gender-inclusive care

## Slide 15
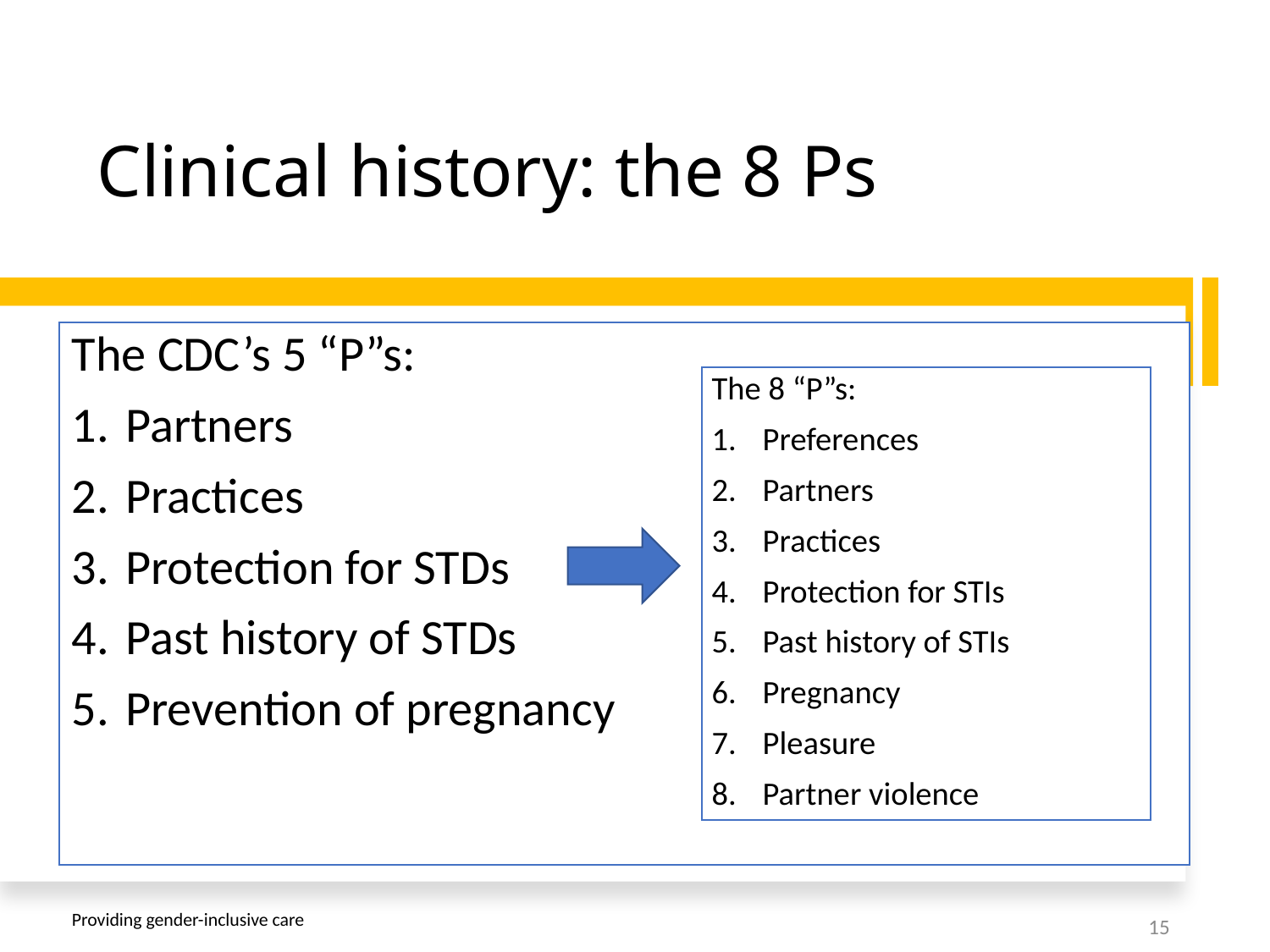

# Clinical history: the 8 Ps
The CDC’s 5 “P”s:
Partners
Practices
Protection for STDs
Past history of STDs
Prevention of pregnancy
The 8 “P”s:
Preferences
Partners
Practices
Protection for STIs
Past history of STIs
Pregnancy
Pleasure
Partner violence
Providing gender-inclusive care
15

## Slide 16
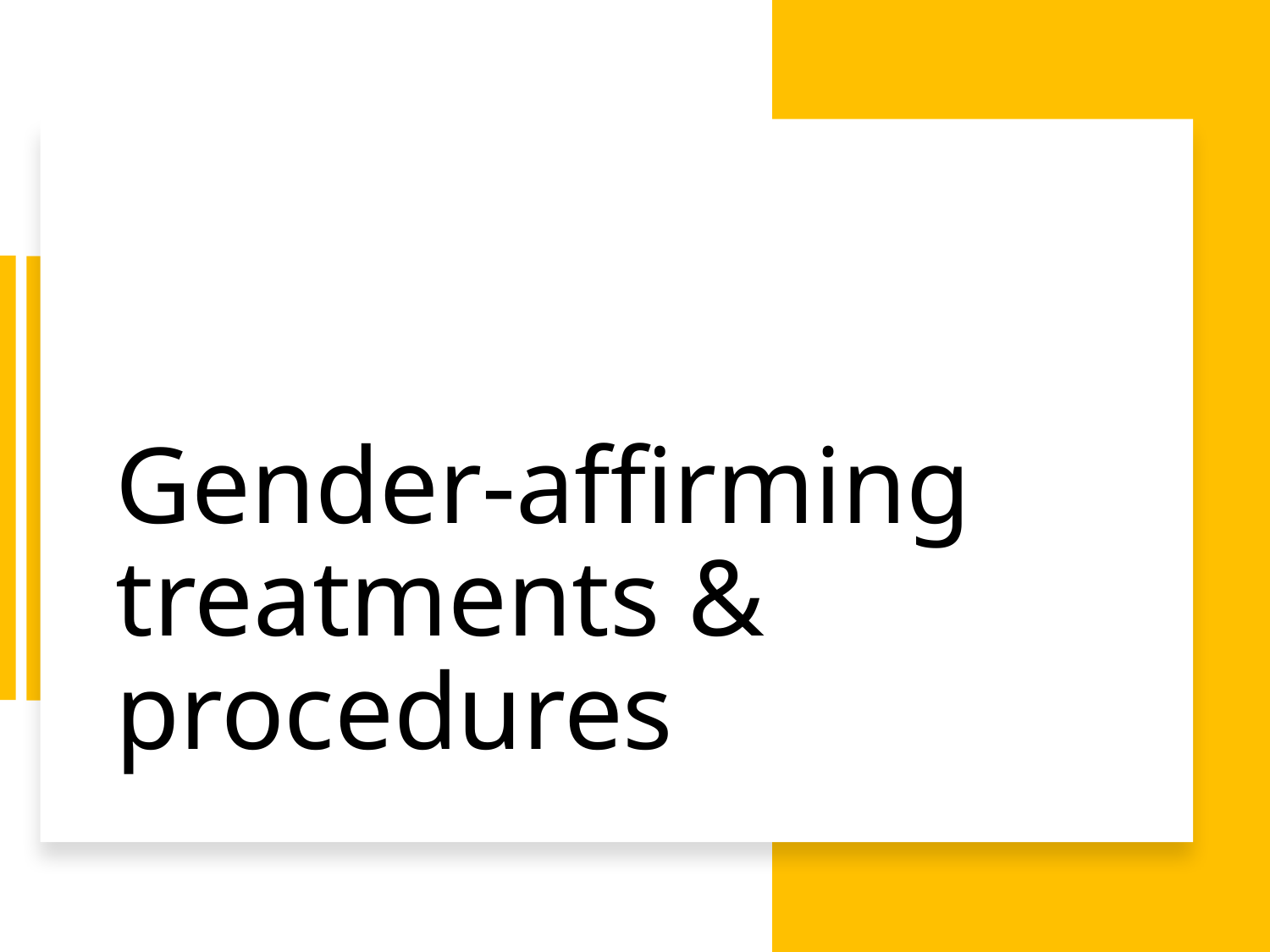

# Gender-affirming treatments & procedures
16

## Slide 17
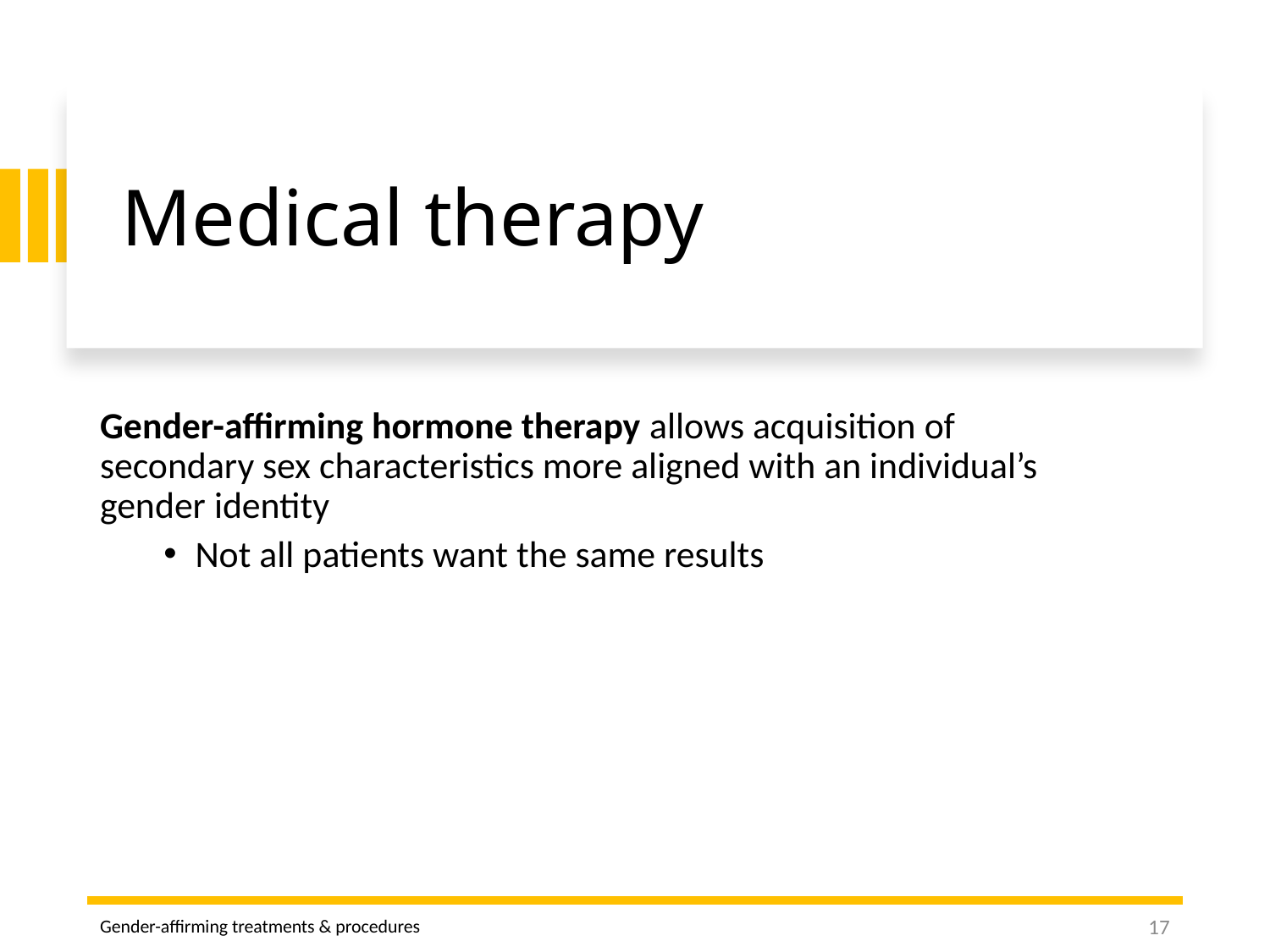

# Medical therapy
Gender-affirming hormone therapy allows acquisition of secondary sex characteristics more aligned with an individual’s gender identity
Not all patients want the same results
17
Gender-affirming treatments & procedures

## Slide 18
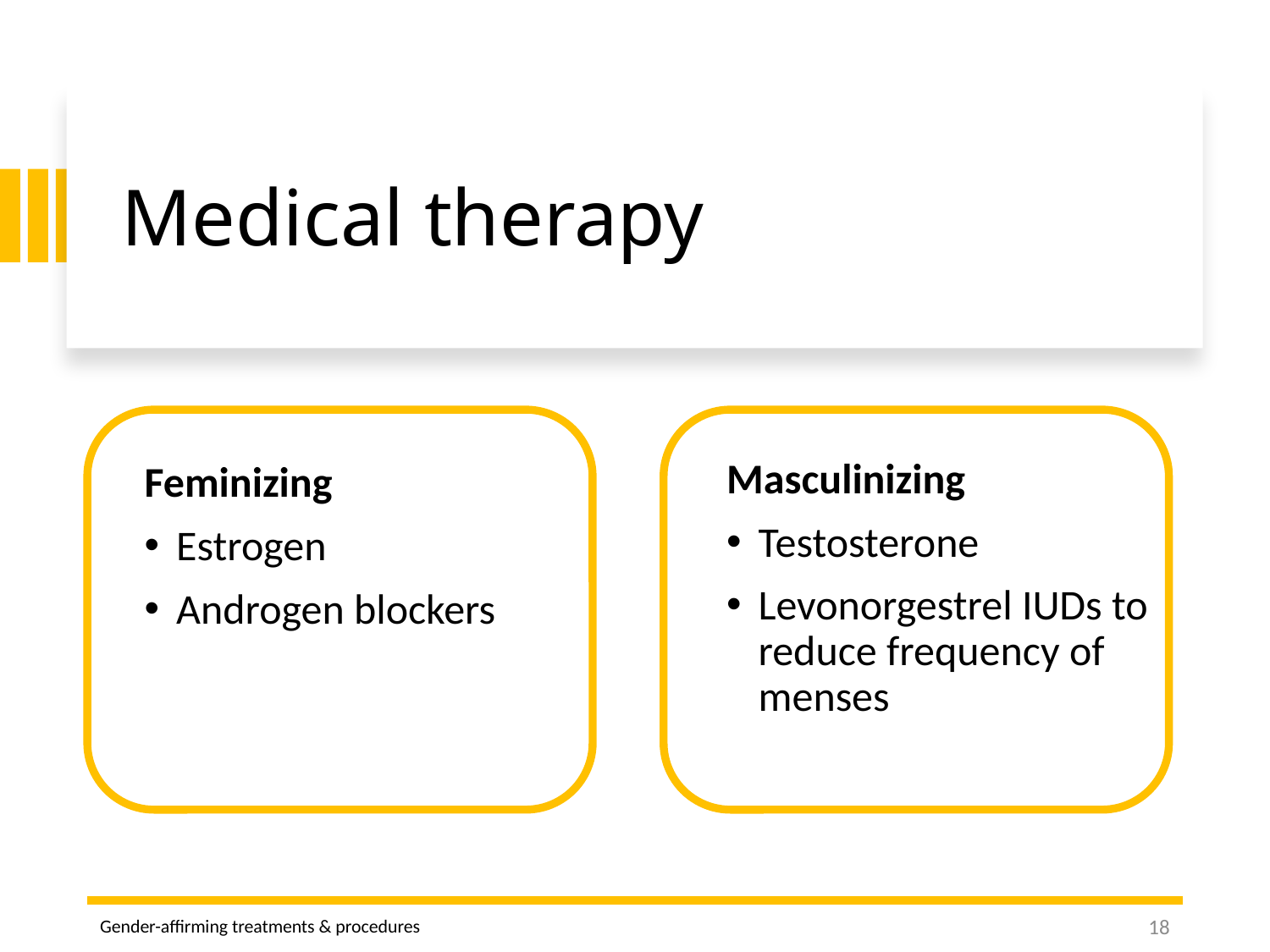

# Medical therapy
Masculinizing
Testosterone
Levonorgestrel IUDs to reduce frequency of menses
Feminizing
Estrogen
Androgen blockers
18
Gender-affirming treatments & procedures

## Slide 19
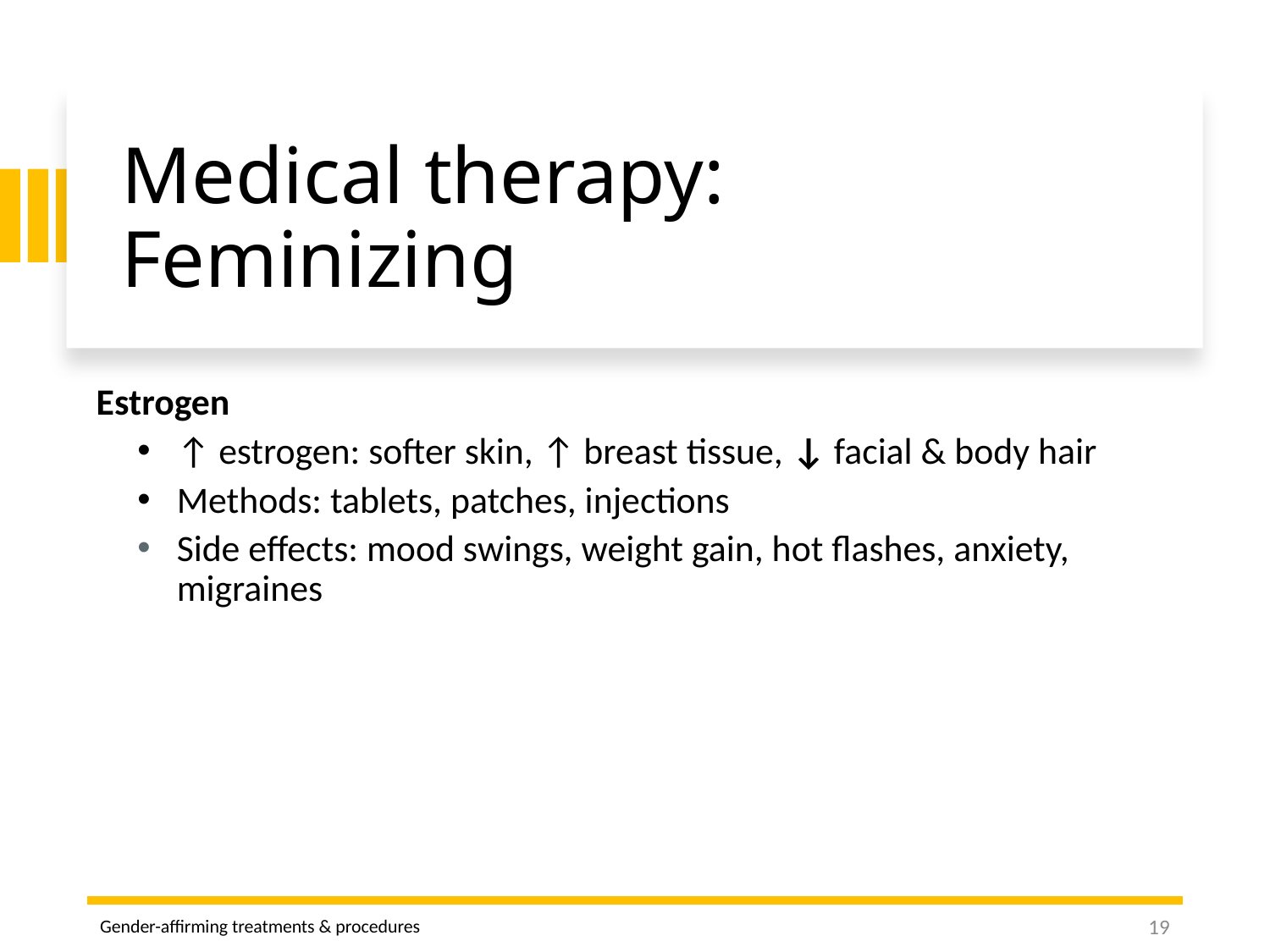

# Medical therapy: Feminizing
Estrogen
↑ estrogen: softer skin, ↑ breast tissue, ↓ facial & body hair
Methods: tablets, patches, injections
Side effects: mood swings, weight gain, hot flashes, anxiety, migraines
19
Gender-affirming treatments & procedures

## Slide 20
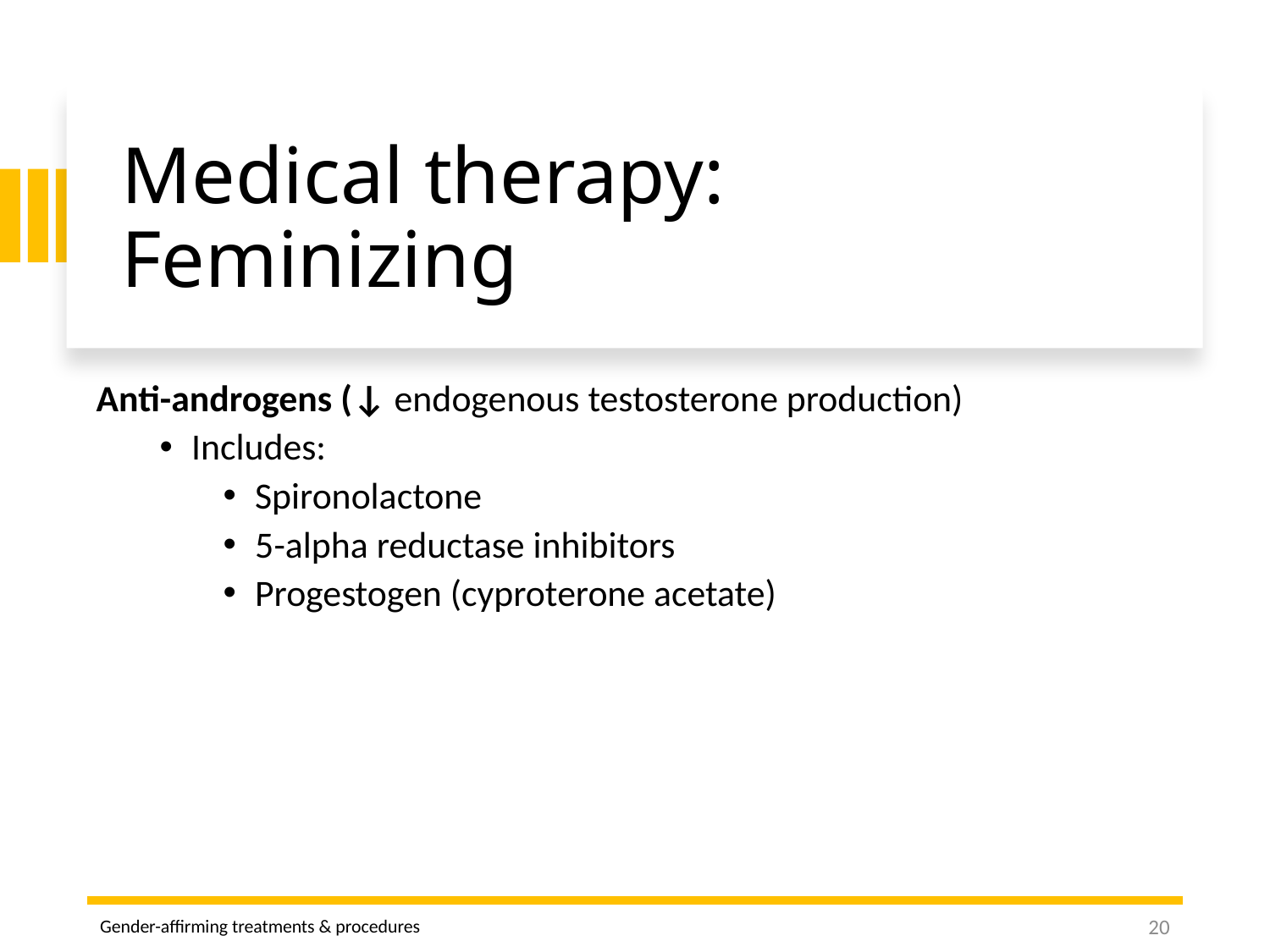

# Medical therapy: Feminizing
Anti-androgens (↓ endogenous testosterone production)
Includes:
Spironolactone
5-alpha reductase inhibitors
Progestogen (cyproterone acetate)
20
Gender-affirming treatments & procedures

## Slide 21
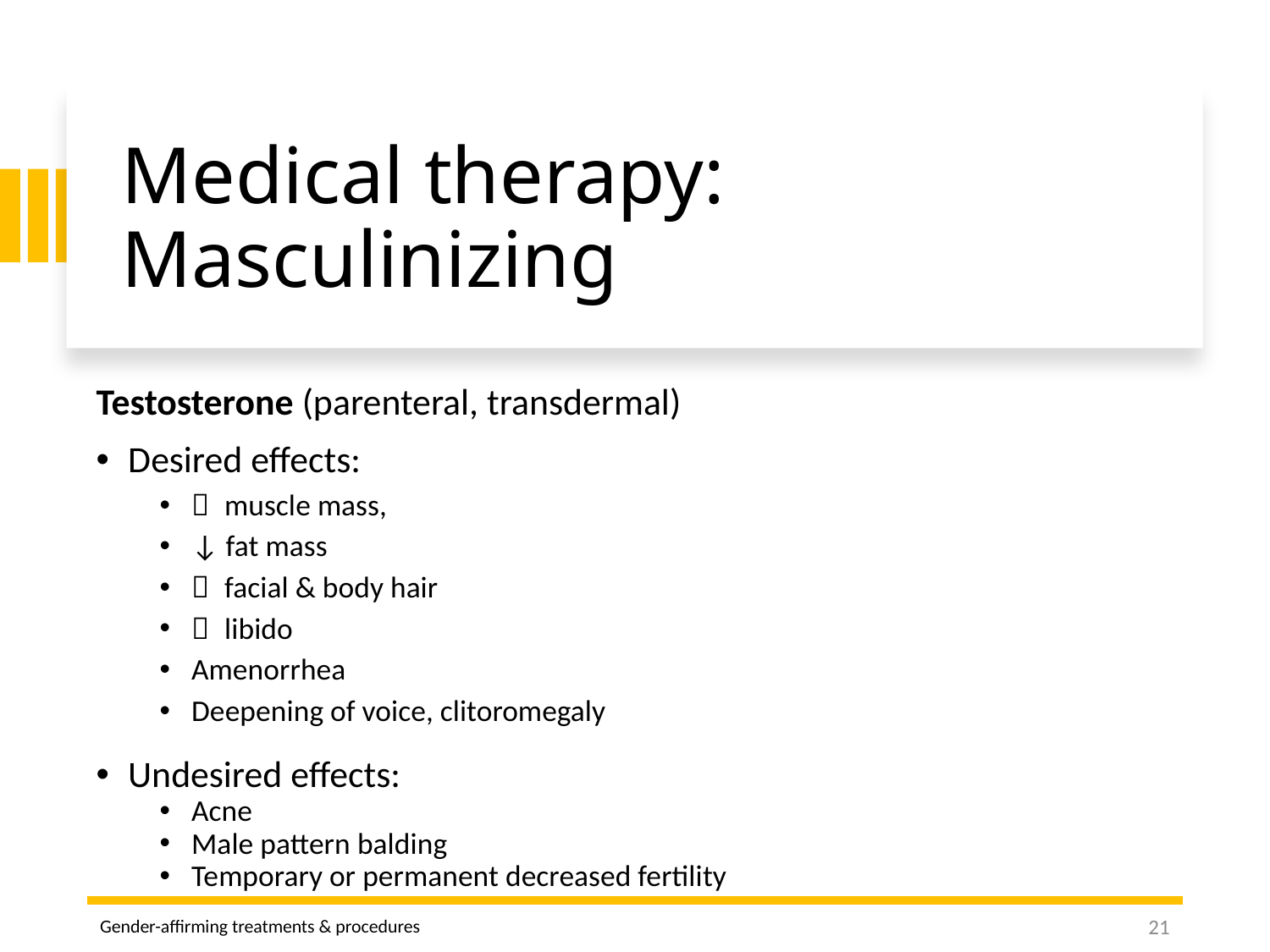

# Medical therapy: Masculinizing
Testosterone (parenteral, transdermal)
Desired effects:
￪ muscle mass,
↓ fat mass
￪ facial & body hair
￪ libido
Amenorrhea
Deepening of voice, clitoromegaly
Undesired effects:
Acne
Male pattern balding
Temporary or permanent decreased fertility
21
Gender-affirming treatments & procedures

## Slide 22
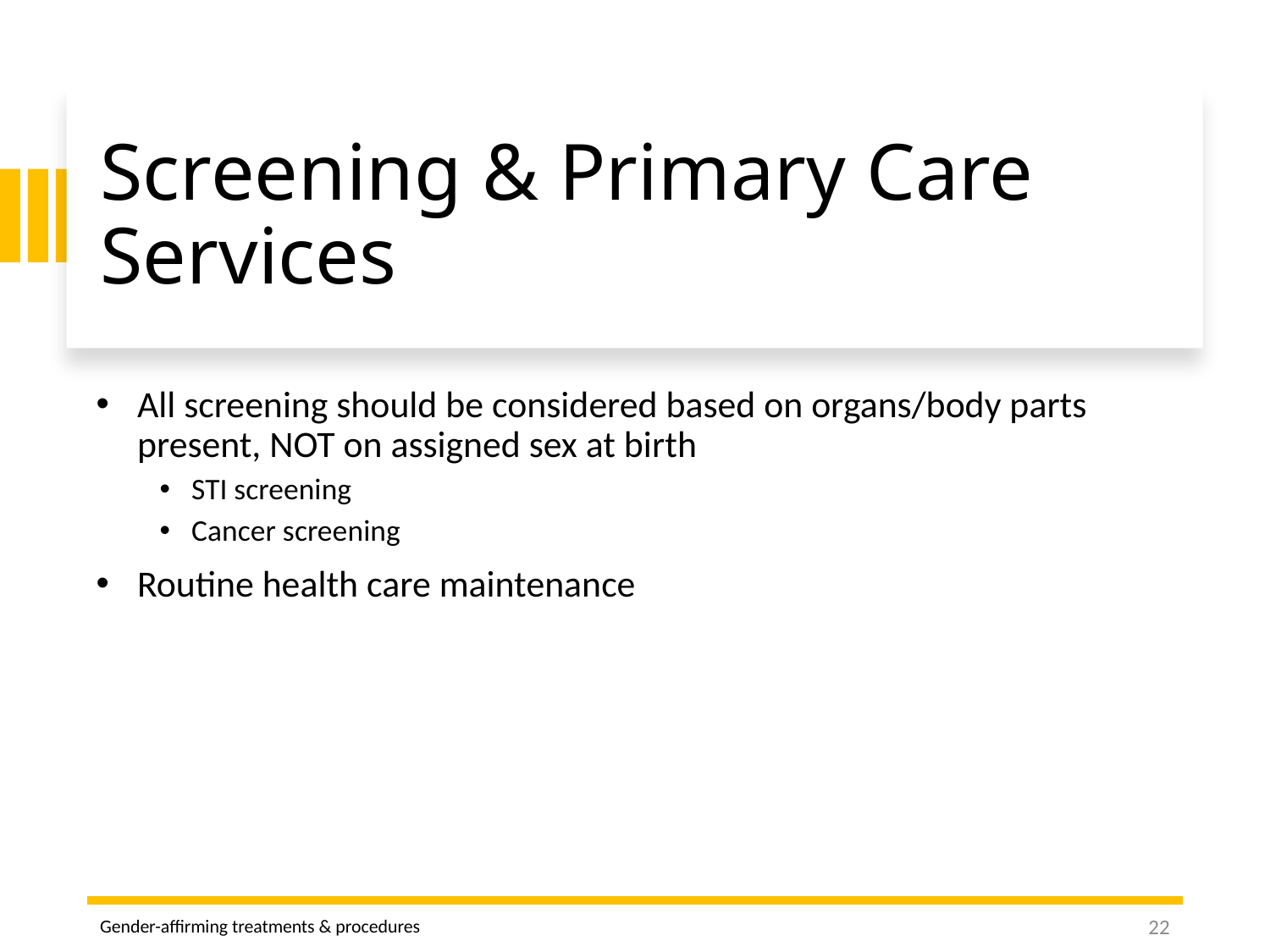

# Screening & Primary Care Services
All screening should be considered based on organs/body parts present, NOT on assigned sex at birth
STI screening
Cancer screening
Routine health care maintenance
22
Gender-affirming treatments & procedures

## Slide 23
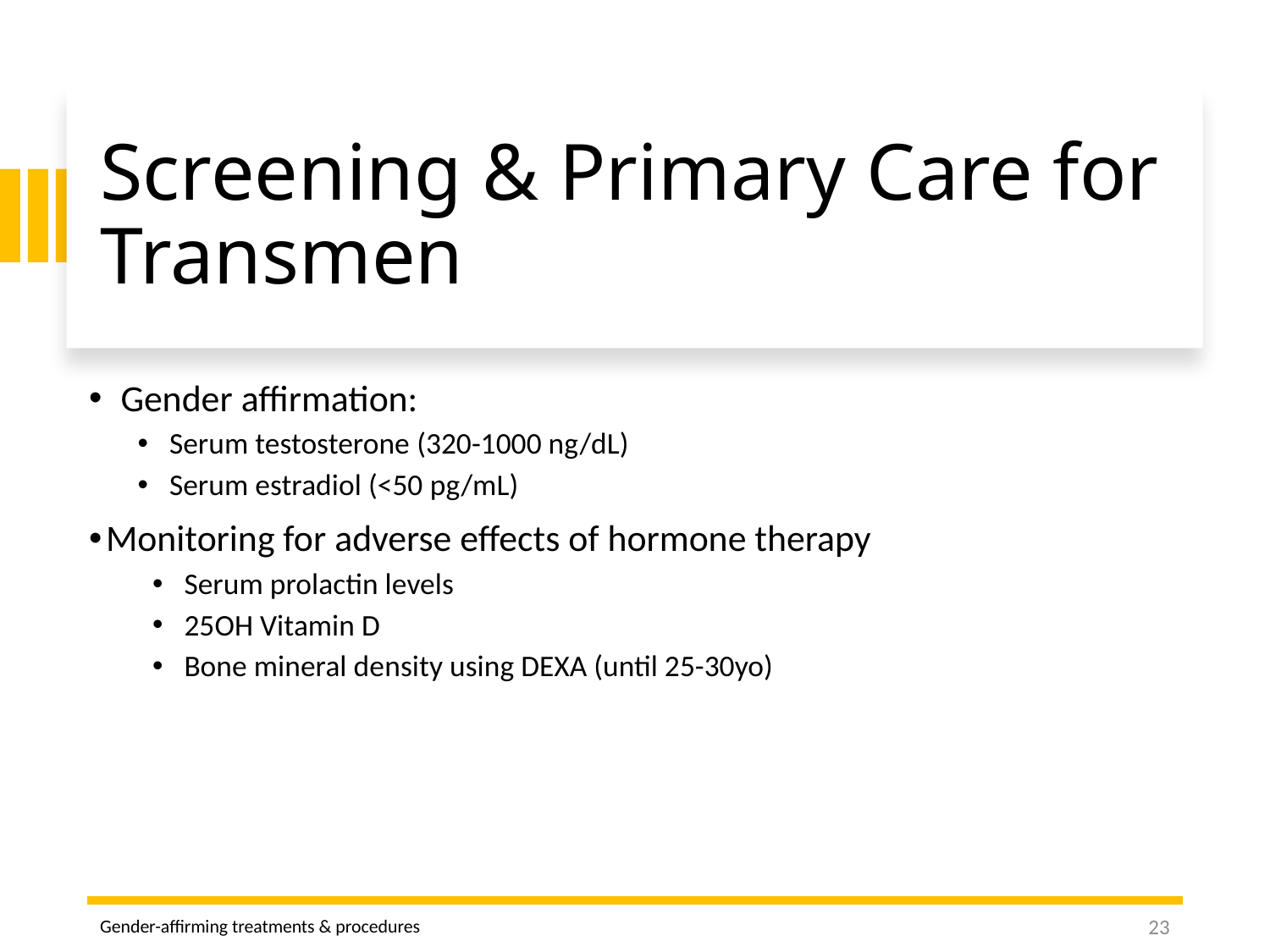

# Screening & Primary Care for Transmen
Gender affirmation:
Serum testosterone (320-1000 ng/dL)
Serum estradiol (<50 pg/mL)
Monitoring for adverse effects of hormone therapy
Serum prolactin levels
25OH Vitamin D
Bone mineral density using DEXA (until 25-30yo)
23
Gender-affirming treatments & procedures

## Slide 24
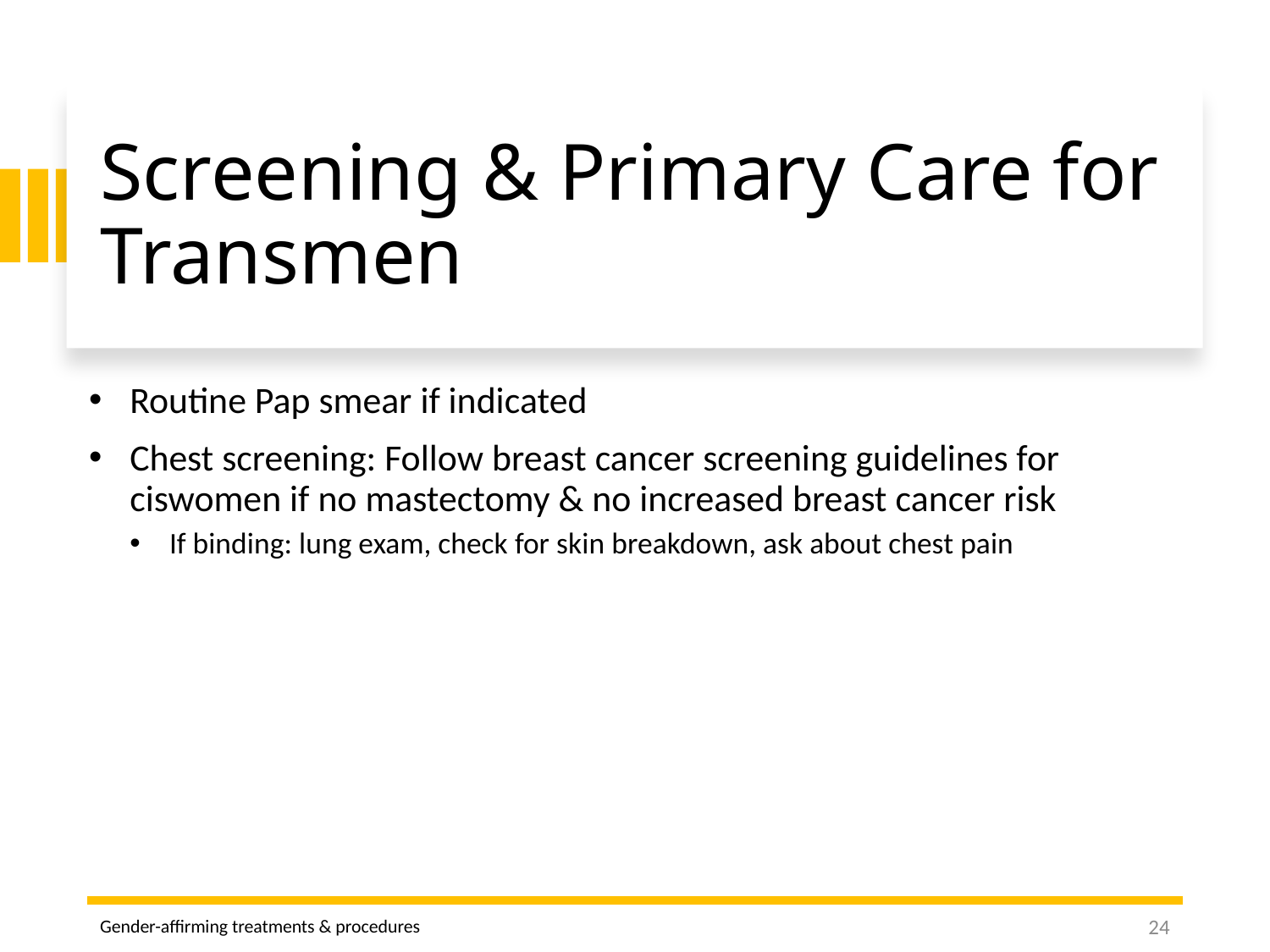

# Screening & Primary Care for Transmen
Routine Pap smear if indicated
Chest screening: Follow breast cancer screening guidelines for ciswomen if no mastectomy & no increased breast cancer risk
If binding: lung exam, check for skin breakdown, ask about chest pain
24
Gender-affirming treatments & procedures

## Slide 25
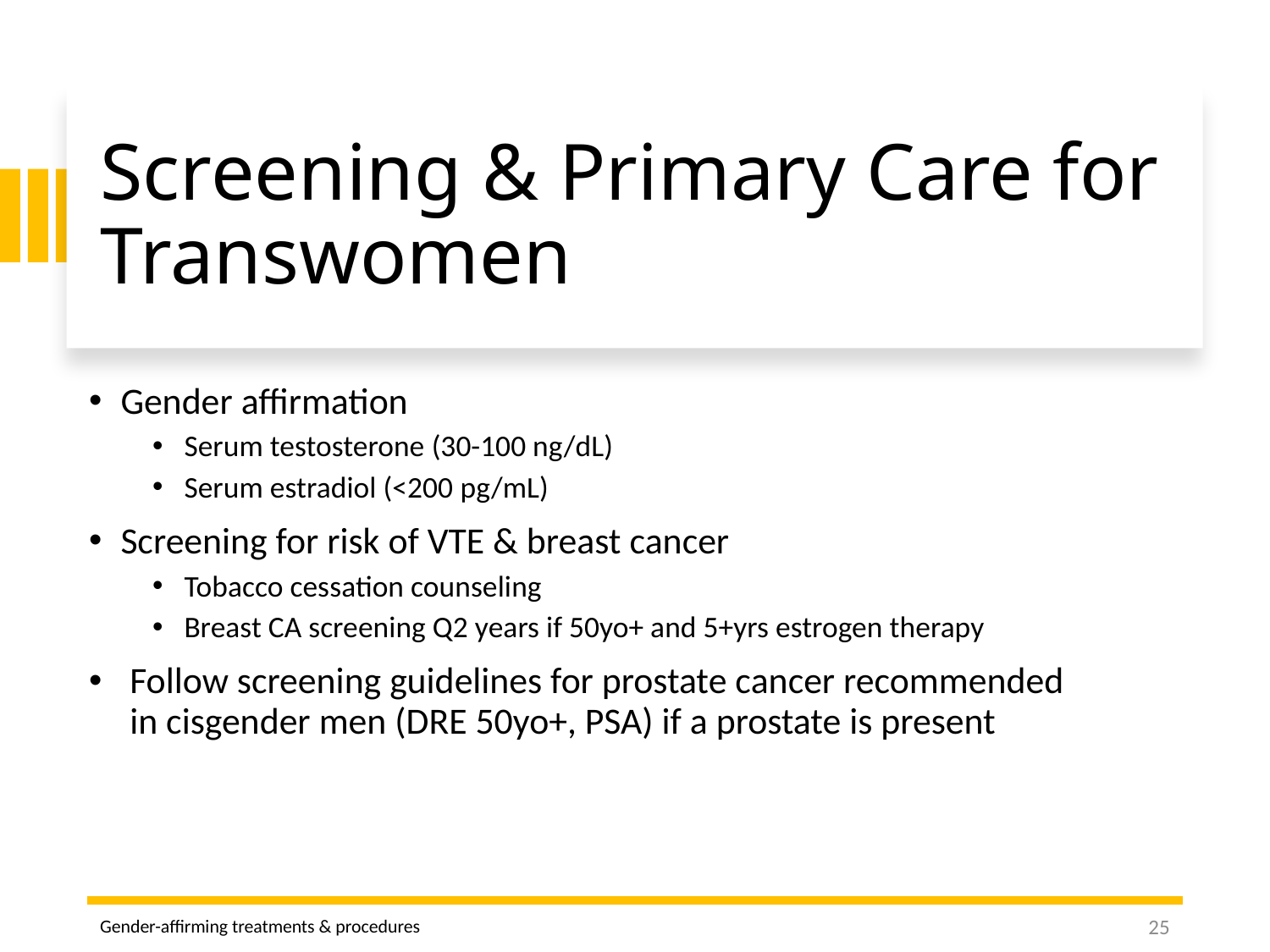

# Screening & Primary Care for Transwomen
Gender affirmation
Serum testosterone (30-100 ng/dL)
Serum estradiol (<200 pg/mL)
Screening for risk of VTE & breast cancer
Tobacco cessation counseling
Breast CA screening Q2 years if 50yo+ and 5+yrs estrogen therapy
Follow screening guidelines for prostate cancer recommended in cisgender men (DRE 50yo+, PSA) if a prostate is present
25
Gender-affirming treatments & procedures

## Slide 26
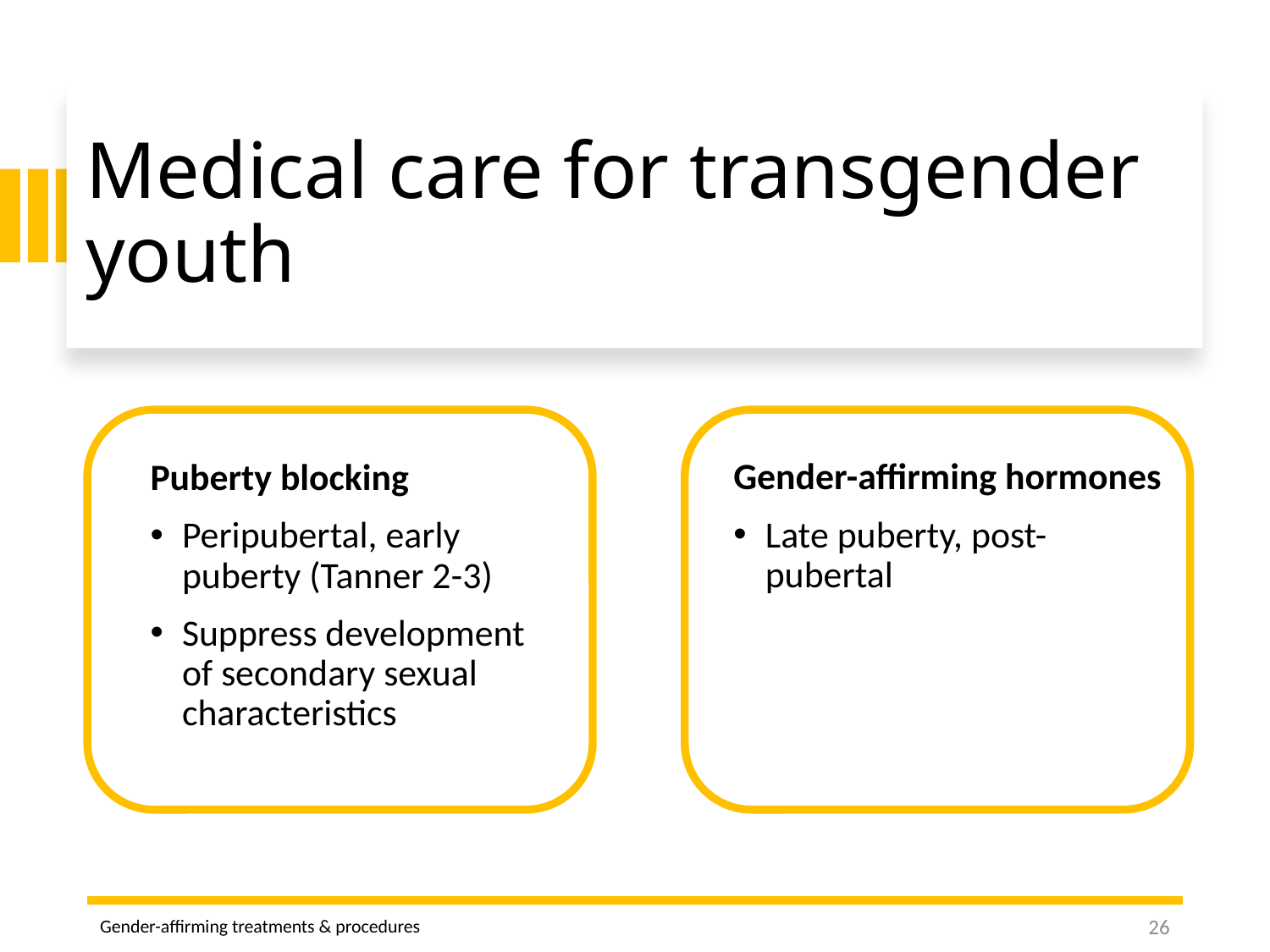

# Medical care for transgender youth
Gender-affirming hormones
Late puberty, post-pubertal
Puberty blocking
Peripubertal, early puberty (Tanner 2-3)
Suppress development of secondary sexual characteristics
26
Gender-affirming treatments & procedures

## Slide 27
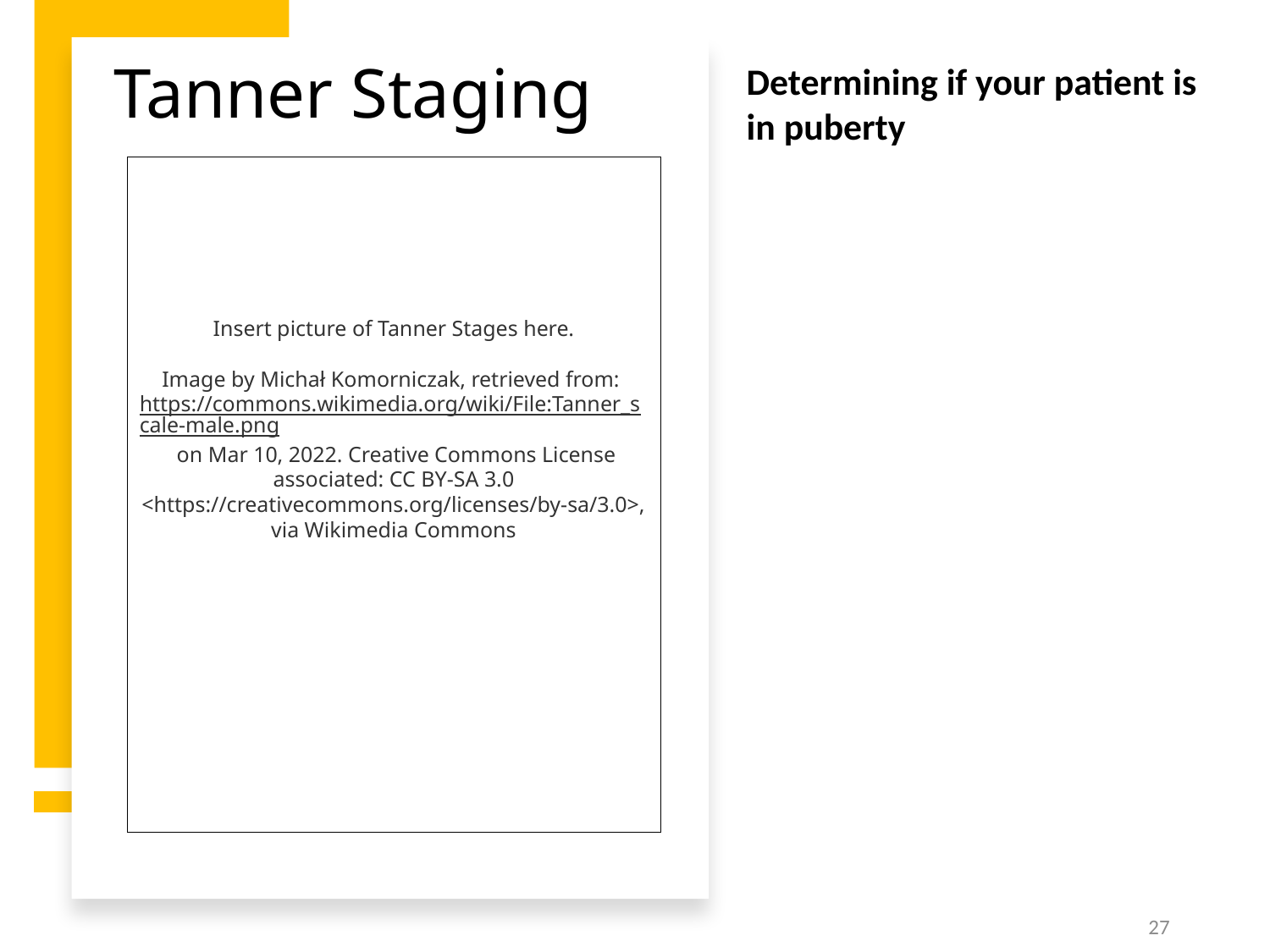

# Tanner Staging
Determining if your patient is in puberty
Insert picture of Tanner Stages here.
Image by Michał Komorniczak, retrieved from: https://commons.wikimedia.org/wiki/File:Tanner_scale-male.png on Mar 10, 2022. Creative Commons License associated: CC BY-SA 3.0 <https://creativecommons.org/licenses/by-sa/3.0>, via Wikimedia Commons
27

## Slide 28
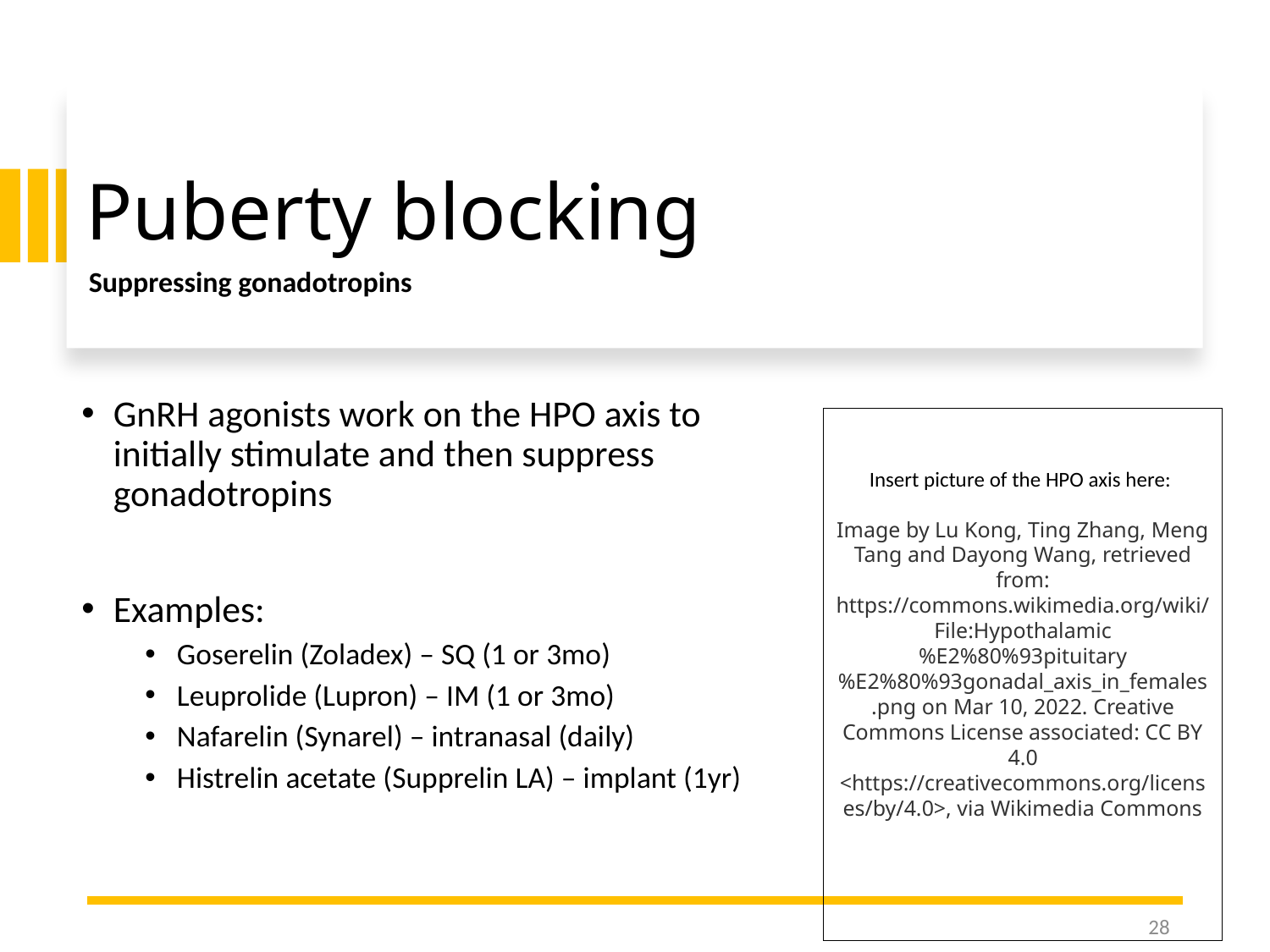

# Puberty blocking
Suppressing gonadotropins
GnRH agonists work on the HPO axis to initially stimulate and then suppress gonadotropins
Examples:
Goserelin (Zoladex) – SQ (1 or 3mo)
Leuprolide (Lupron) – IM (1 or 3mo)
Nafarelin (Synarel) – intranasal (daily)
Histrelin acetate (Supprelin LA) – implant (1yr)
Insert picture of the HPO axis here:
Image by Lu Kong, Ting Zhang, Meng Tang and Dayong Wang, retrieved from: https://commons.wikimedia.org/wiki/File:Hypothalamic%E2%80%93pituitary%E2%80%93gonadal_axis_in_females.png on Mar 10, 2022. Creative Commons License associated: CC BY 4.0 <https://creativecommons.org/licenses/by/4.0>, via Wikimedia Commons
28

## Slide 29
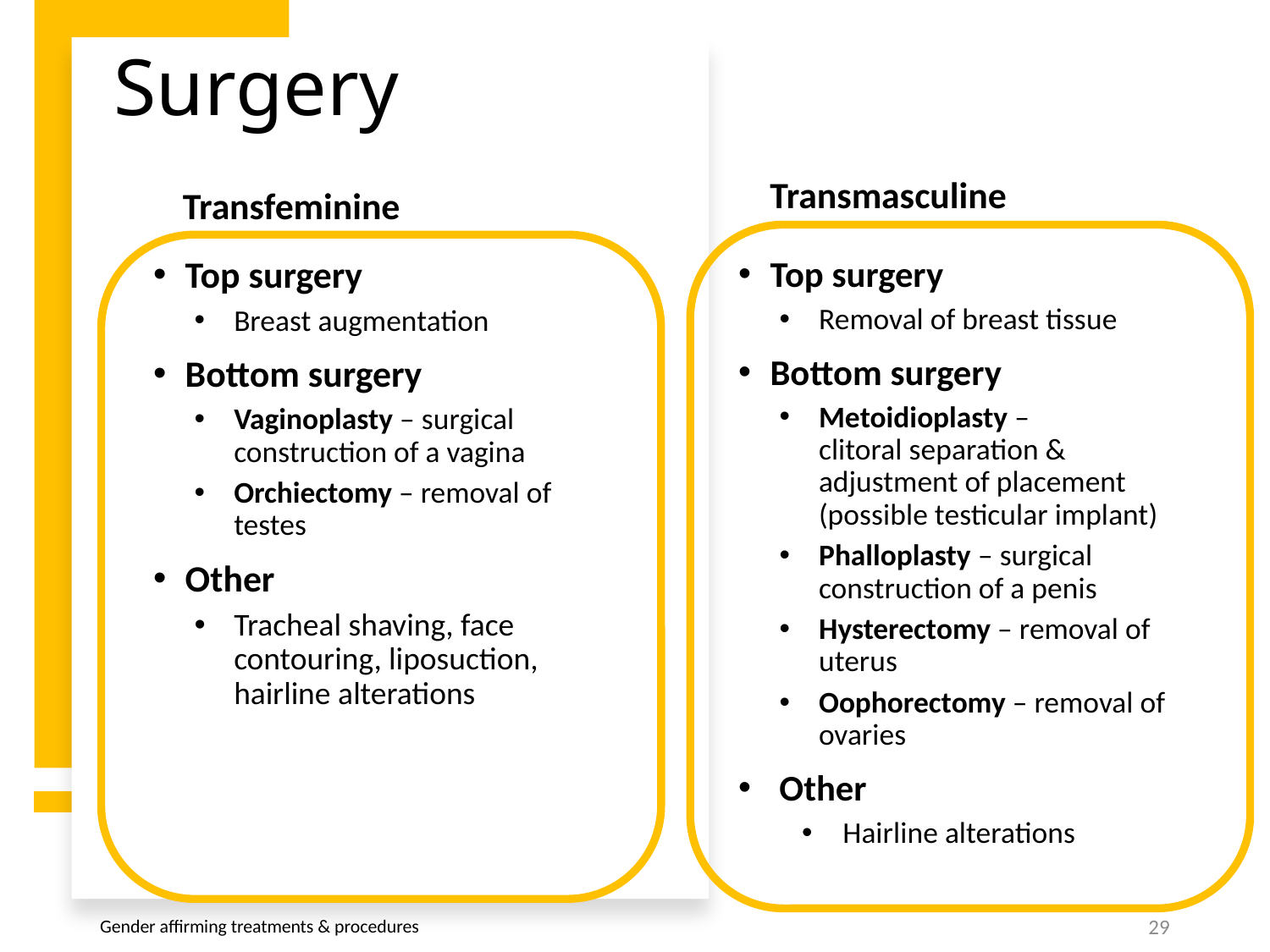

# Surgery
Transmasculine
Transfeminine
Top surgery
Breast augmentation
Bottom surgery
Vaginoplasty – surgical construction of a vagina
Orchiectomy – removal of testes
Other
Tracheal shaving, face contouring, liposuction, hairline alterations
Top surgery
Removal of breast tissue
Bottom surgery
Metoidioplasty –clitoral separation & adjustment of placement (possible testicular implant)
Phalloplasty – surgical construction of a penis
Hysterectomy – removal of uterus
Oophorectomy – removal of ovaries
Other
Hairline alterations
29
Gender affirming treatments & procedures

## Slide 30
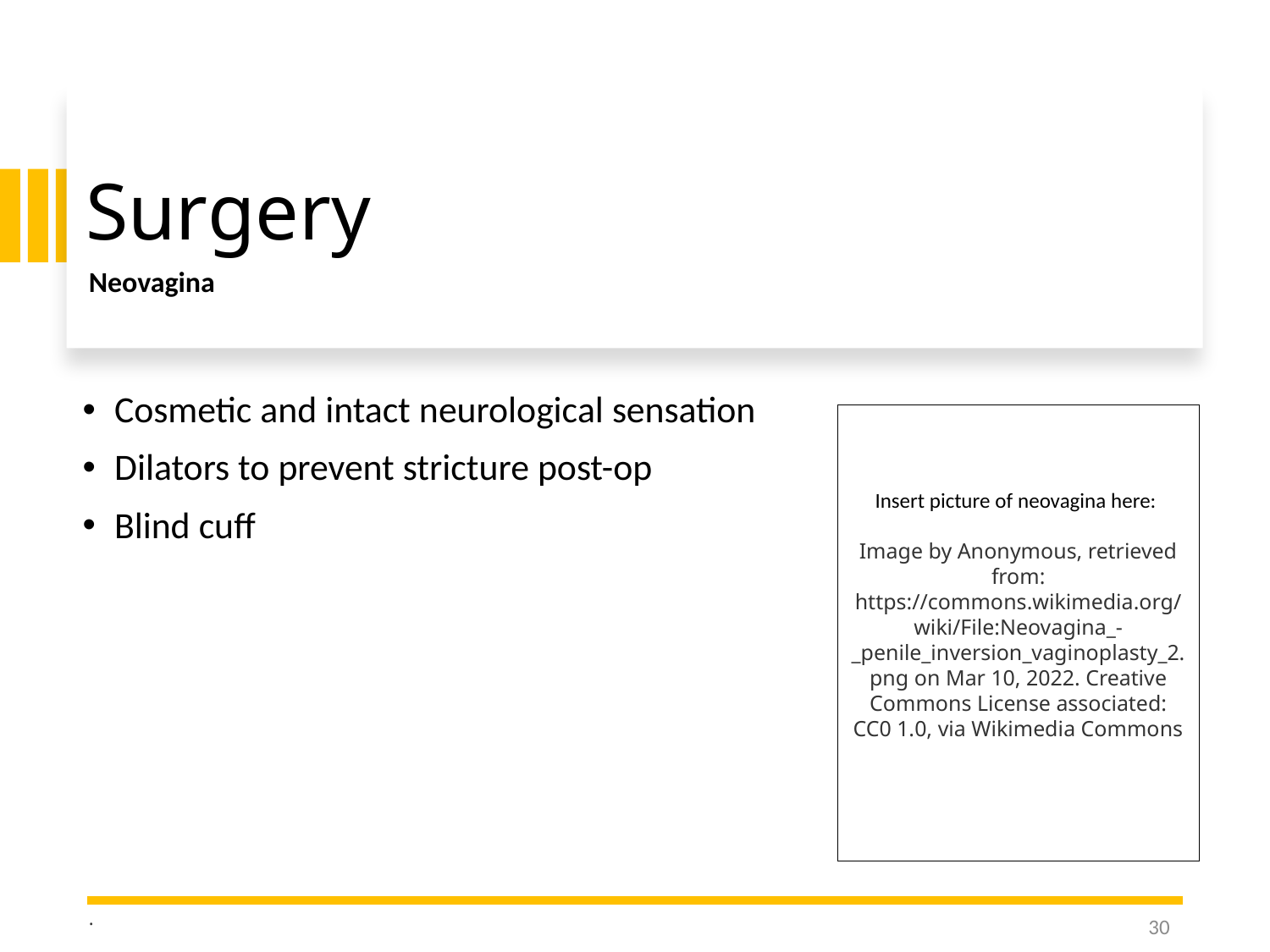

# Surgery
Neovagina
Cosmetic and intact neurological sensation
Dilators to prevent stricture post-op
Blind cuff
Insert picture of neovagina here:
Image by Anonymous, retrieved from: https://commons.wikimedia.org/wiki/File:Neovagina_-_penile_inversion_vaginoplasty_2.png on Mar 10, 2022. Creative Commons License associated: CC0 1.0, via Wikimedia Commons
.
30

## Slide 31
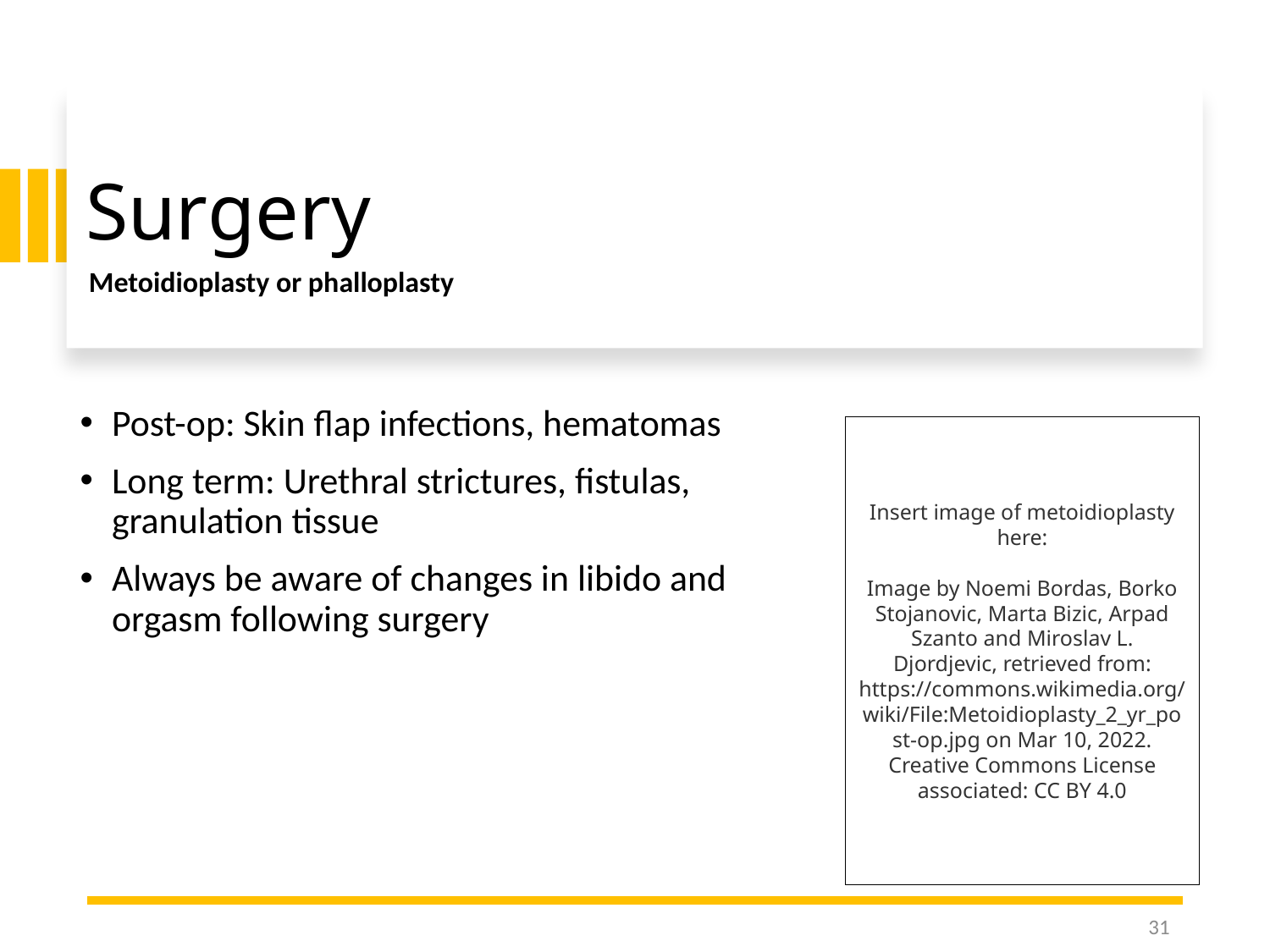

# Surgery
Metoidioplasty or phalloplasty
Post-op: Skin flap infections, hematomas
Long term: Urethral strictures, fistulas, granulation tissue
Always be aware of changes in libido and orgasm following surgery
Insert image of metoidioplasty here:
Image by Noemi Bordas, Borko Stojanovic, Marta Bizic, Arpad Szanto and Miroslav L. Djordjevic, retrieved from: https://commons.wikimedia.org/wiki/File:Metoidioplasty_2_yr_post-op.jpg on Mar 10, 2022. Creative Commons License associated: CC BY 4.0
31

## Slide 32
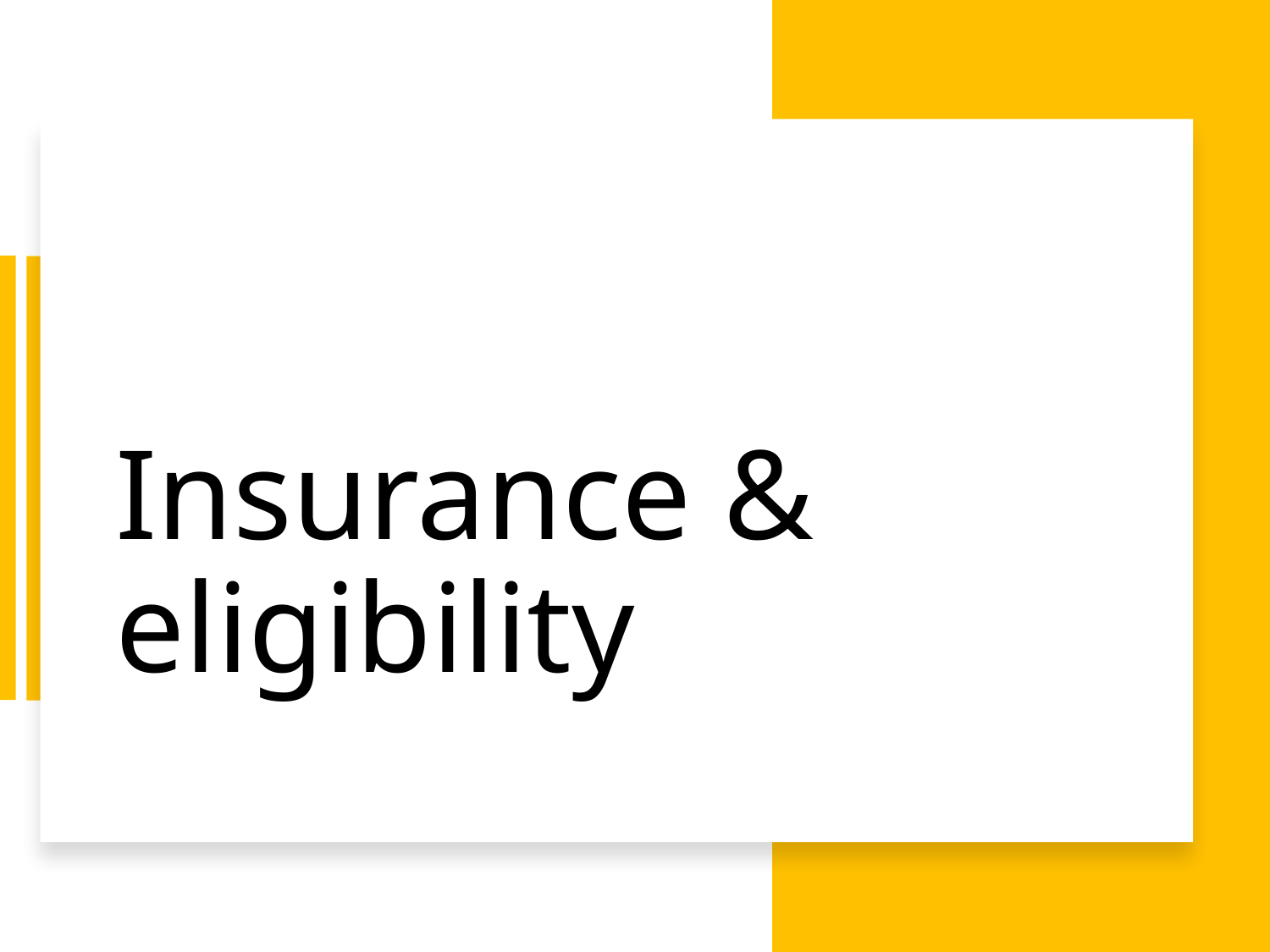

# Insurance & eligibility
32

## Slide 33
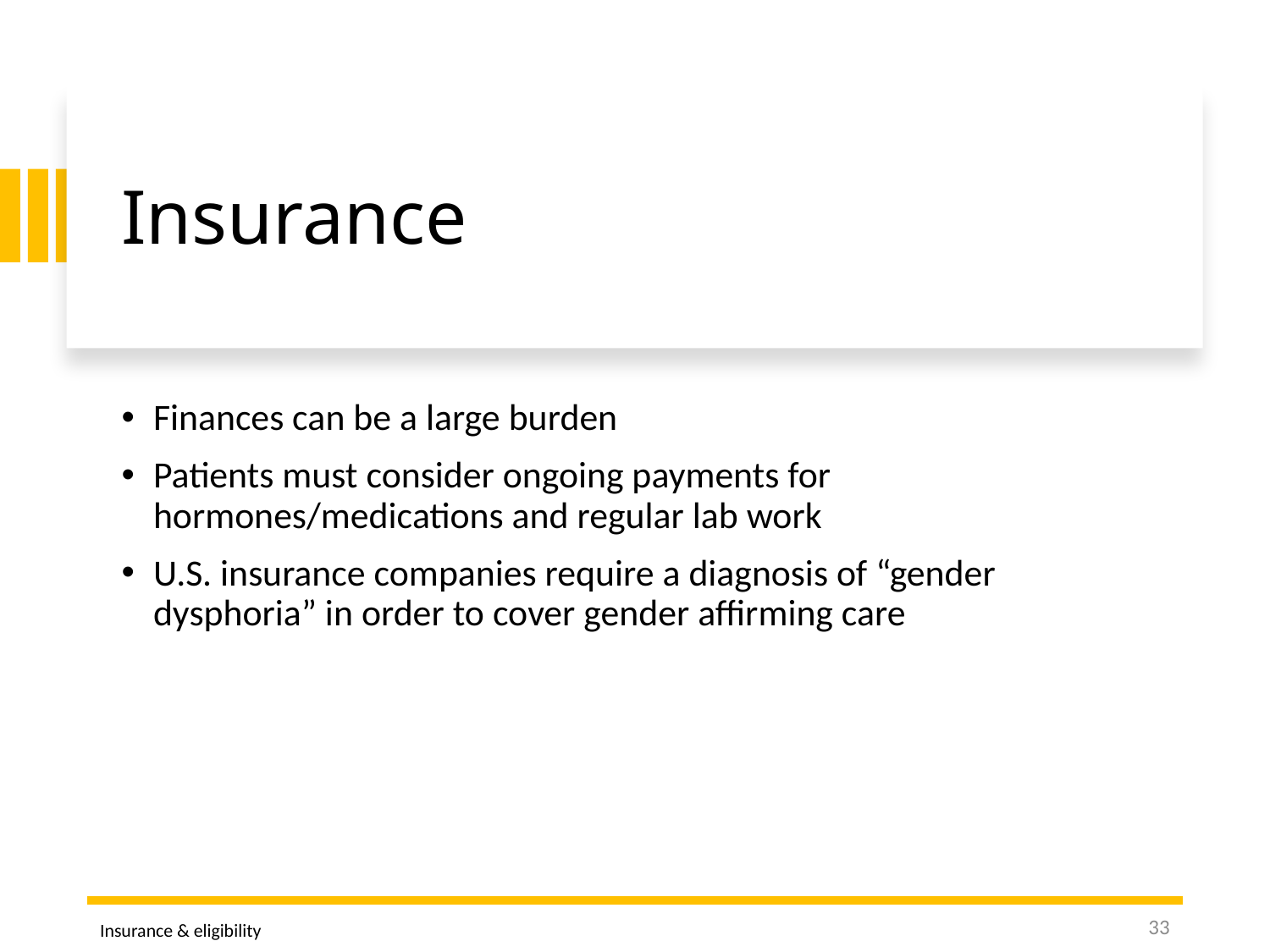

# Insurance
Finances can be a large burden
Patients must consider ongoing payments for hormones/medications and regular lab work
U.S. insurance companies require a diagnosis of “gender dysphoria” in order to cover gender affirming care
33
Insurance & eligibility

## Slide 34
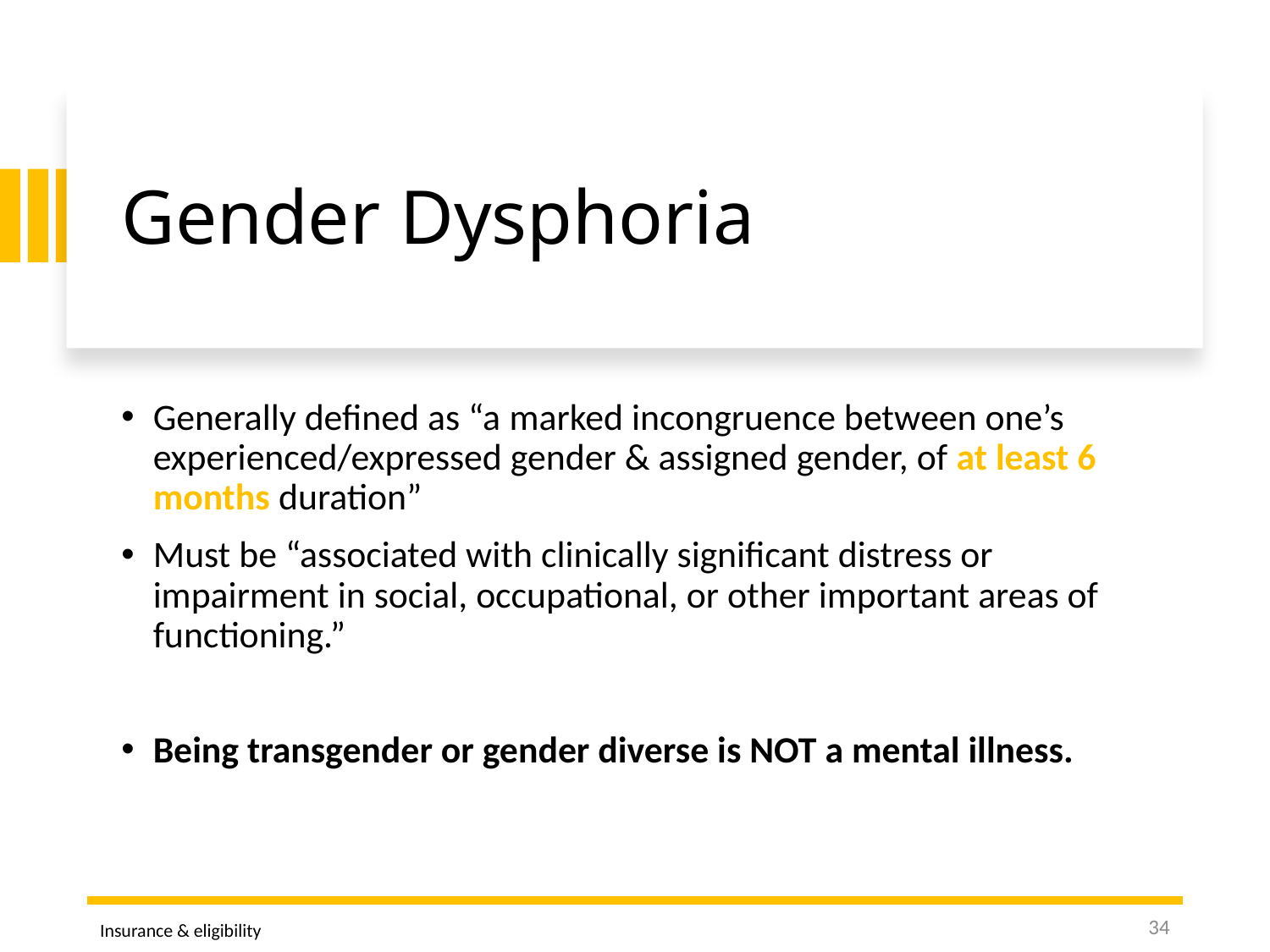

# Gender Dysphoria
Generally defined as “a marked incongruence between one’s experienced/expressed gender & assigned gender, of at least 6 months duration”
Must be “associated with clinically significant distress or impairment in social, occupational, or other important areas of functioning.”
Being transgender or gender diverse is NOT a mental illness.
34
Insurance & eligibility

## Slide 35
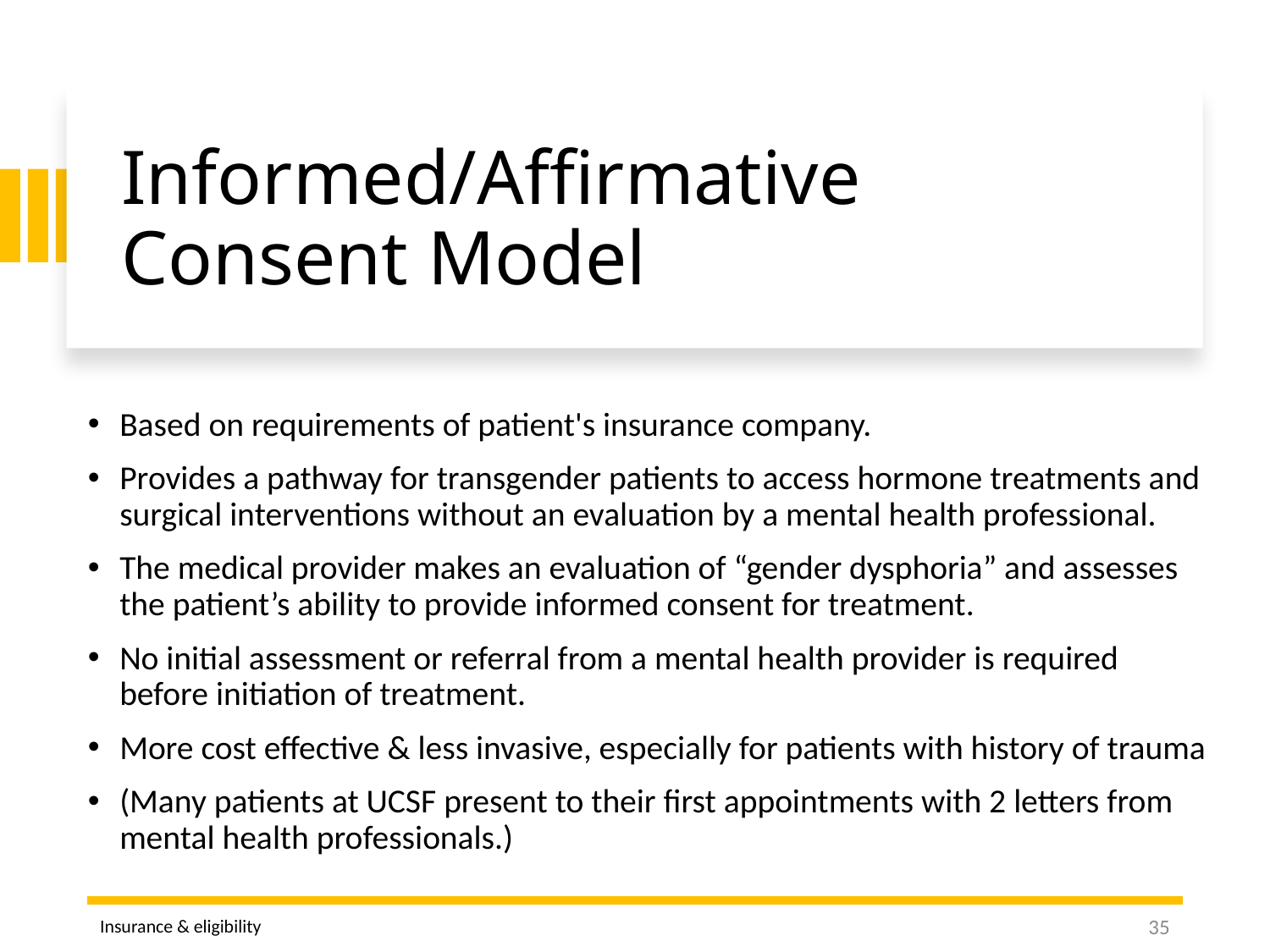

# Informed/Affirmative Consent Model
Based on requirements of patient's insurance company.
Provides a pathway for transgender patients to access hormone treatments and surgical interventions without an evaluation by a mental health professional.
The medical provider makes an evaluation of “gender dysphoria” and assesses the patient’s ability to provide informed consent for treatment.
No initial assessment or referral from a mental health provider is required before initiation of treatment.
More cost effective & less invasive, especially for patients with history of trauma
(Many patients at UCSF present to their first appointments with 2 letters from mental health professionals.)
35
Insurance & eligibility

## Slide 36
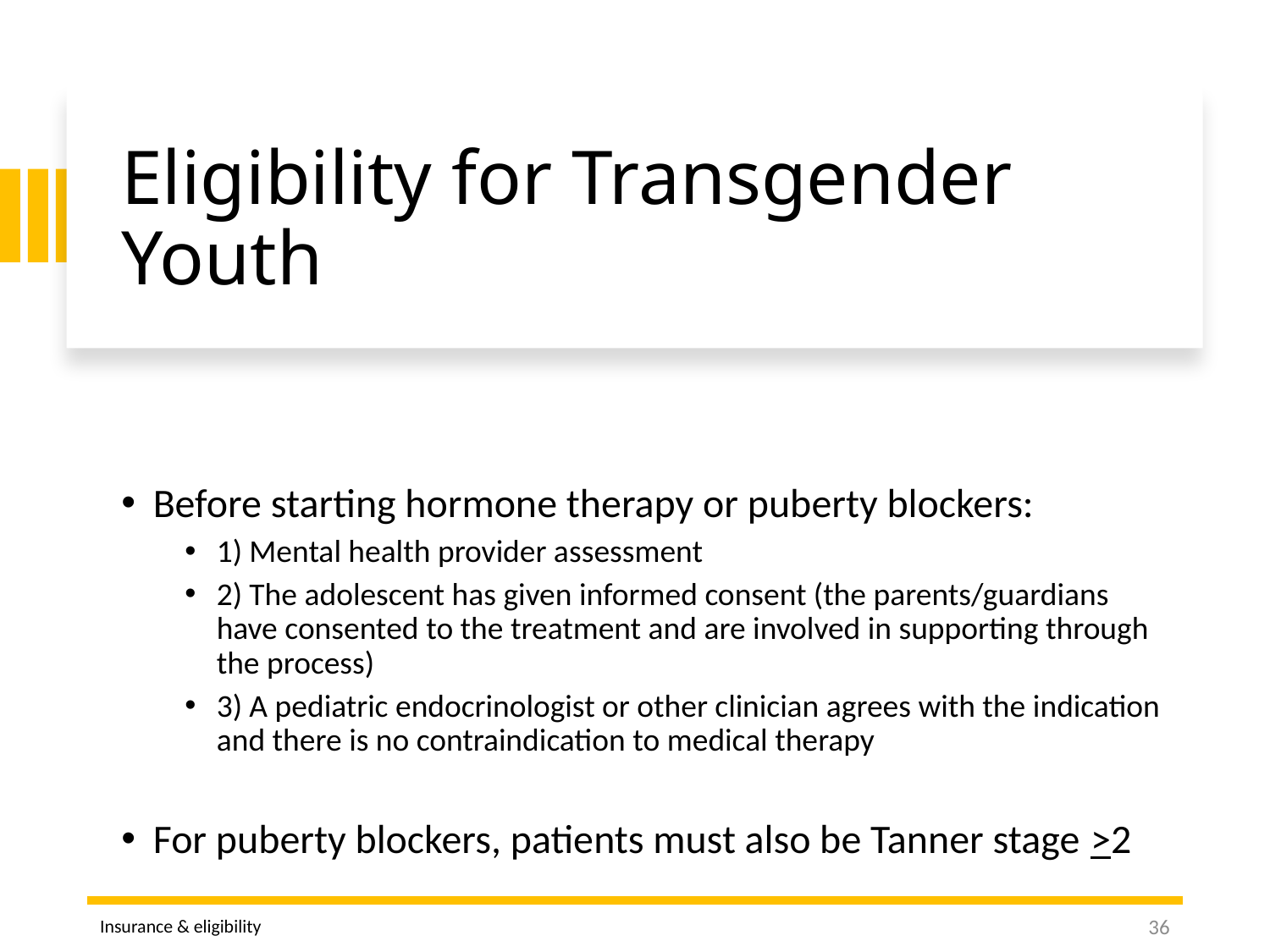

# Eligibility for Transgender Youth
Before starting hormone therapy or puberty blockers:
1) Mental health provider assessment
2) The adolescent has given informed consent (the parents/guardians have consented to the treatment and are involved in supporting through the process)
3) A pediatric endocrinologist or other clinician agrees with the indication and there is no contraindication to medical therapy
For puberty blockers, patients must also be Tanner stage >2
36
Insurance & eligibility

## Slide 37
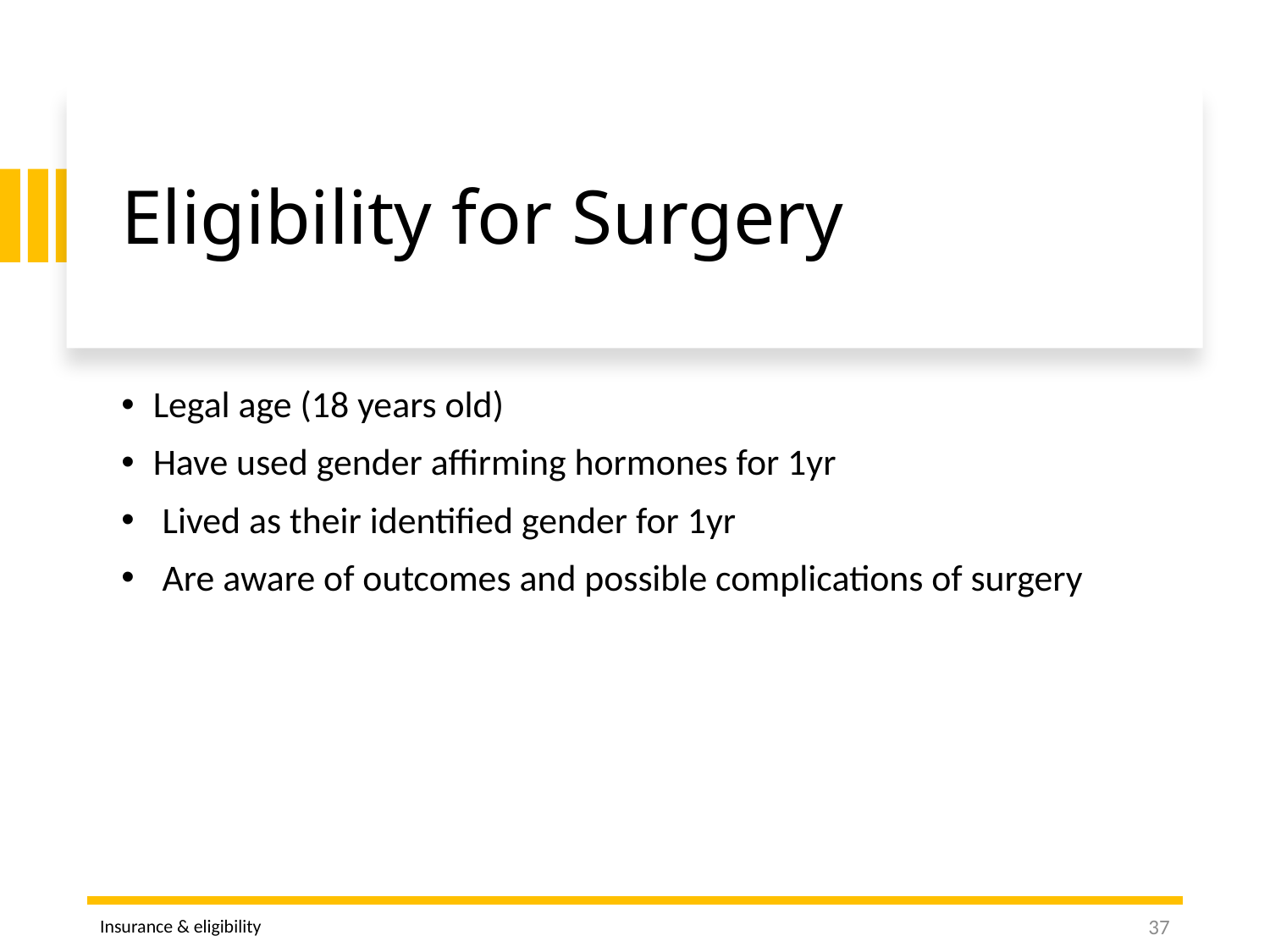

# Eligibility for Surgery
Legal age (18 years old)
Have used gender affirming hormones for 1yr
Lived as their identified gender for 1yr
Are aware of outcomes and possible complications of surgery
37
Insurance & eligibility

## Slide 38
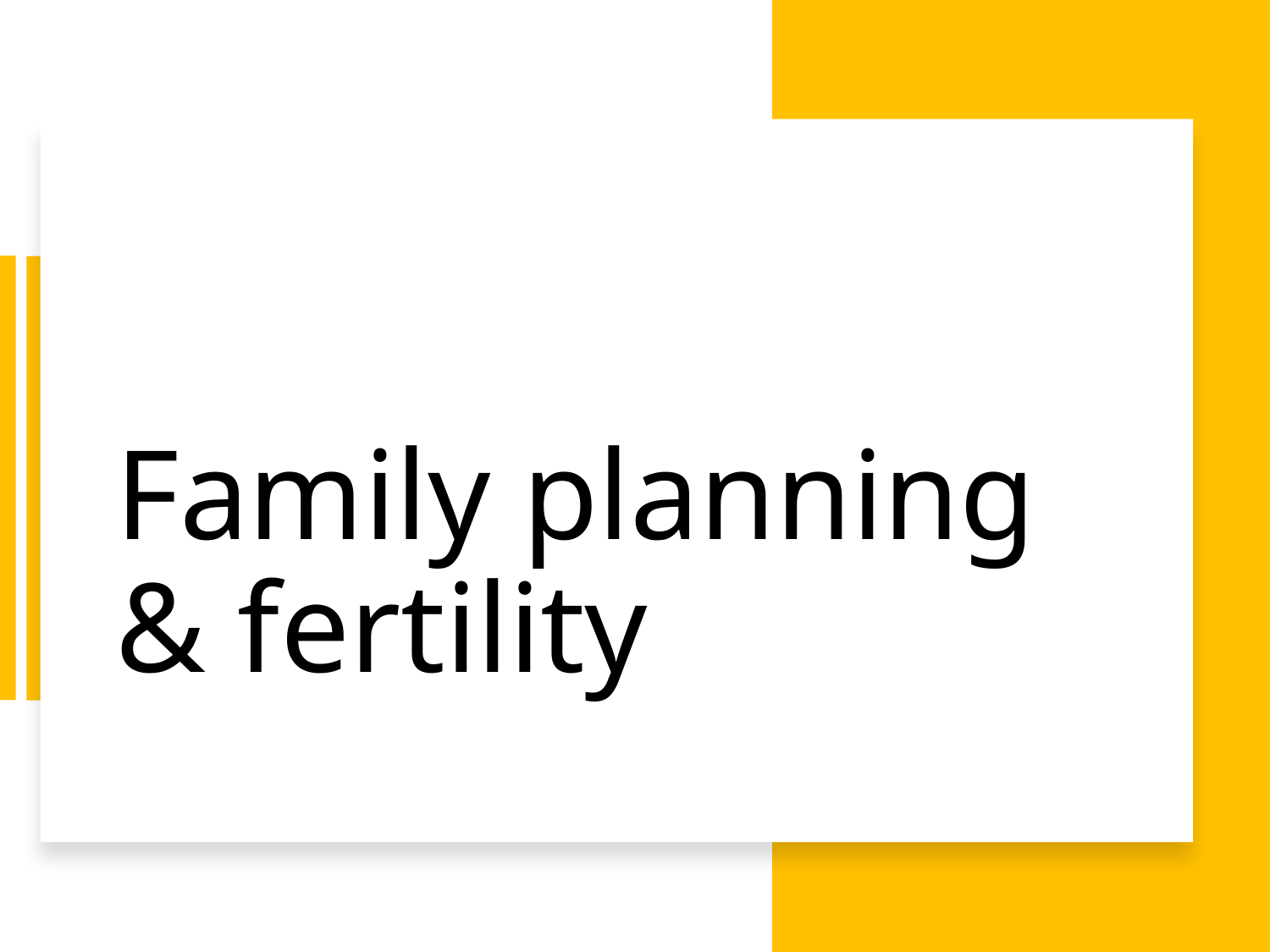

# Family planning & fertility
38

## Slide 39
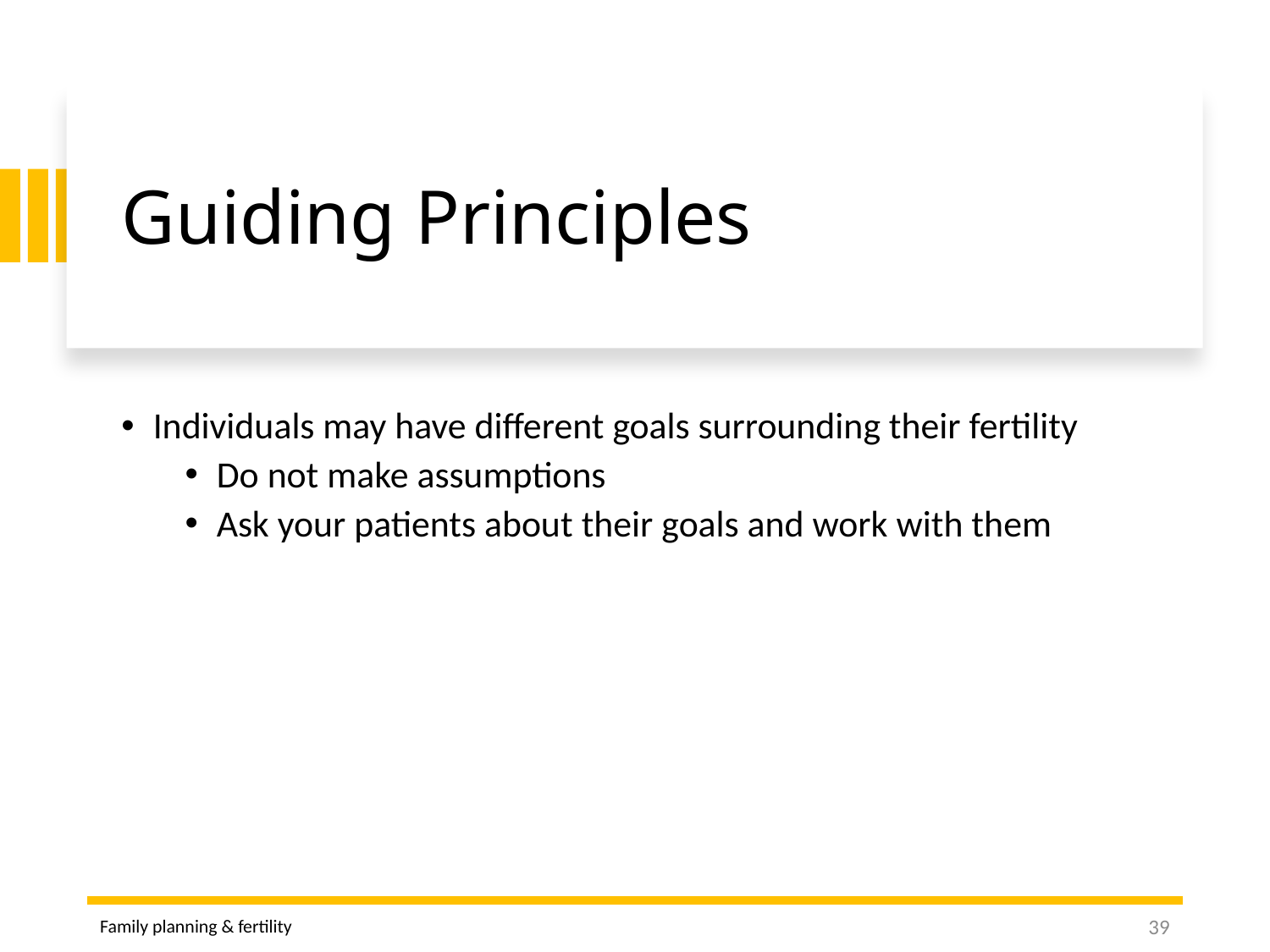

# Guiding Principles
Individuals may have different goals surrounding their fertility
Do not make assumptions
Ask your patients about their goals and work with them
39
Family planning & fertility

## Slide 40
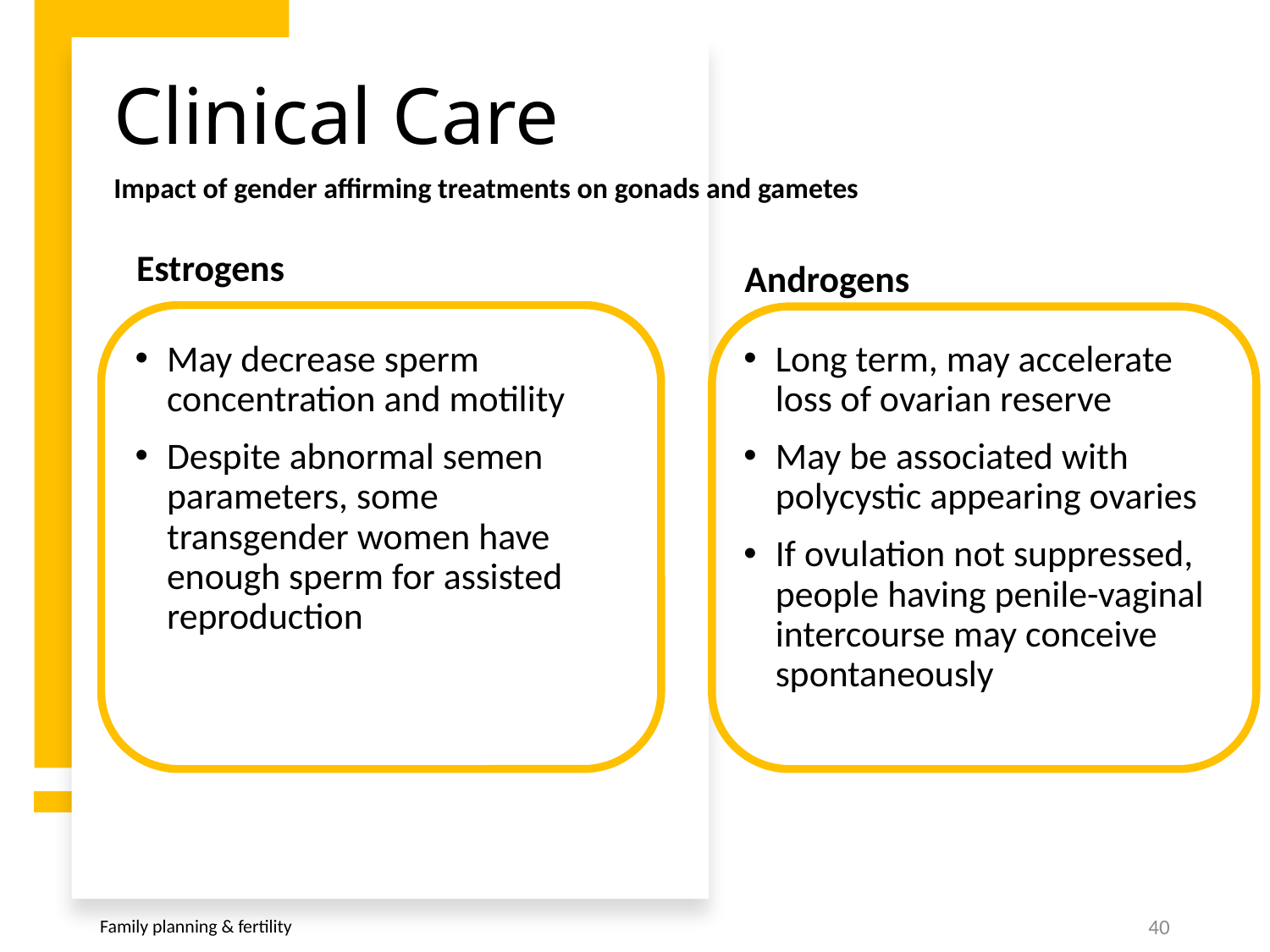

# Clinical Care
Impact of gender affirming treatments on gonads and gametes
Estrogens
Androgens
May decrease sperm concentration and motility
Despite abnormal semen parameters, some transgender women have enough sperm for assisted reproduction
Long term, may accelerate loss of ovarian reserve
May be associated with polycystic appearing ovaries
If ovulation not suppressed, people having penile-vaginal intercourse may conceive spontaneously
40
Family planning & fertility

## Slide 41
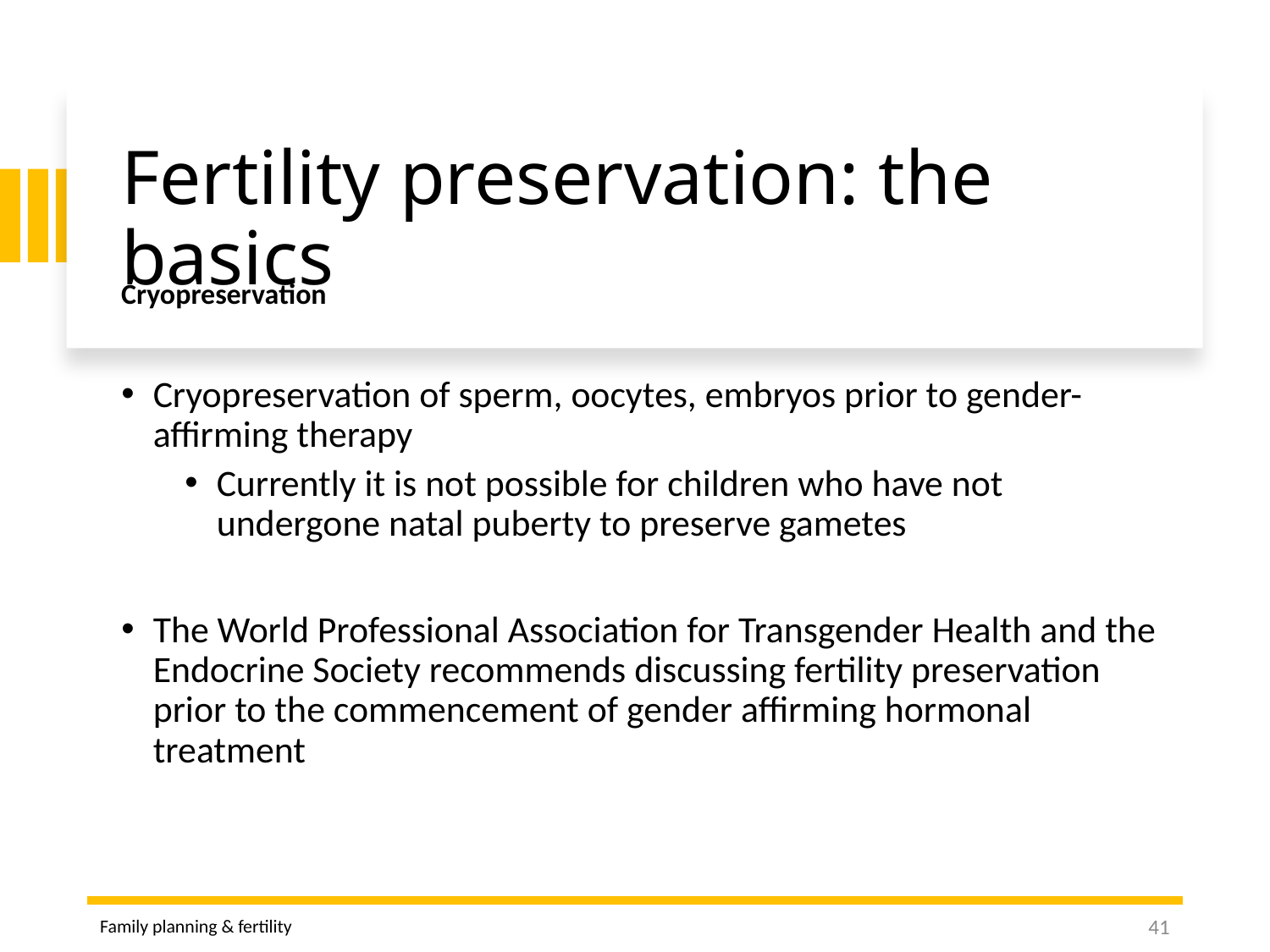

# Fertility preservation: the basics
Cryopreservation
Cryopreservation of sperm, oocytes, embryos prior to gender-affirming therapy
Currently it is not possible for children who have not undergone natal puberty to preserve gametes
The World Professional Association for Transgender Health and the Endocrine Society recommends discussing fertility preservation prior to the commencement of gender affirming hormonal treatment
41
Family planning & fertility

## Slide 42
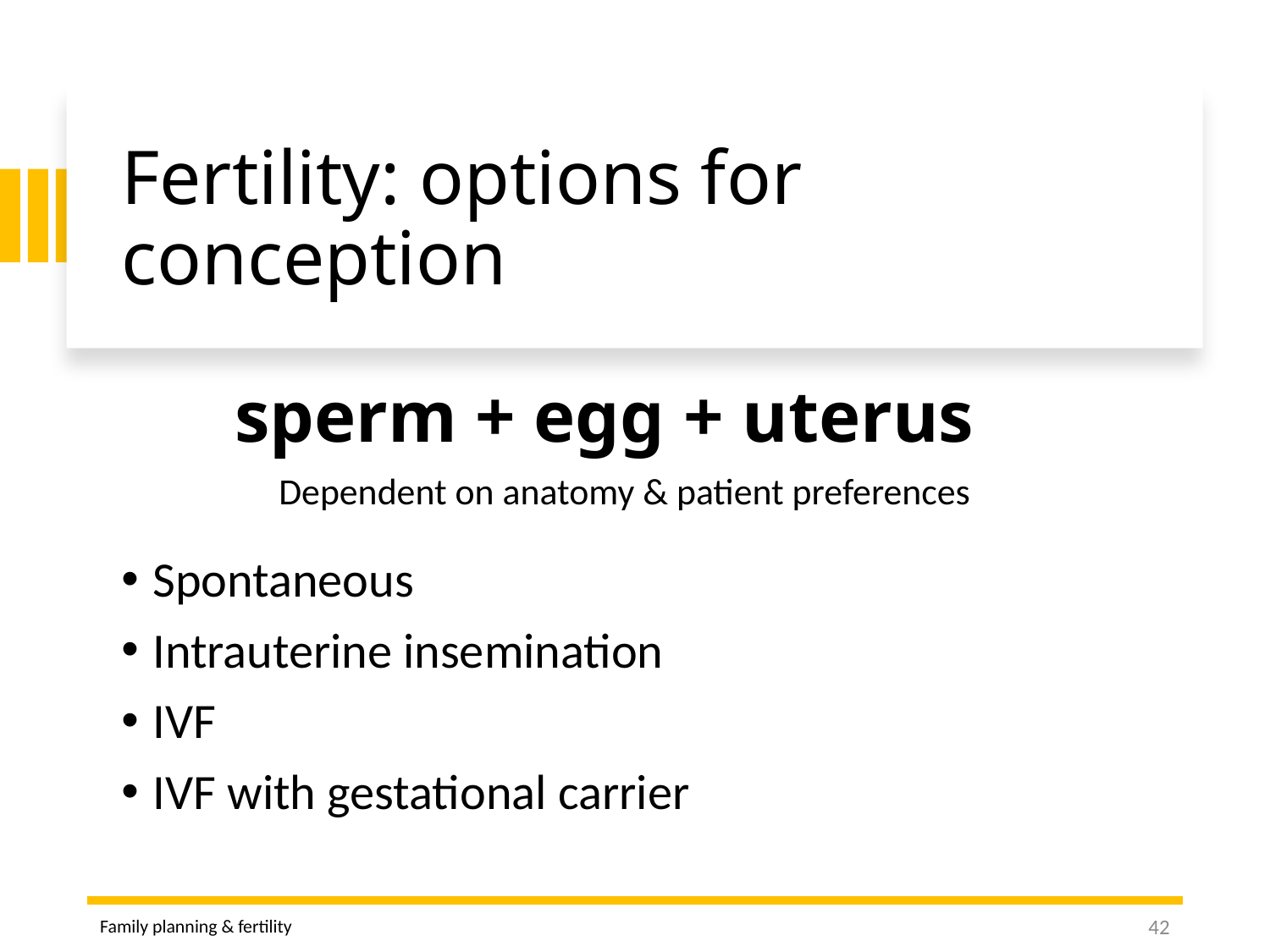

# Fertility: options for conception
sperm + egg + uterus
Dependent on anatomy & patient preferences
Spontaneous
Intrauterine insemination
IVF
IVF with gestational carrier
42
Family planning & fertility

## Slide 43
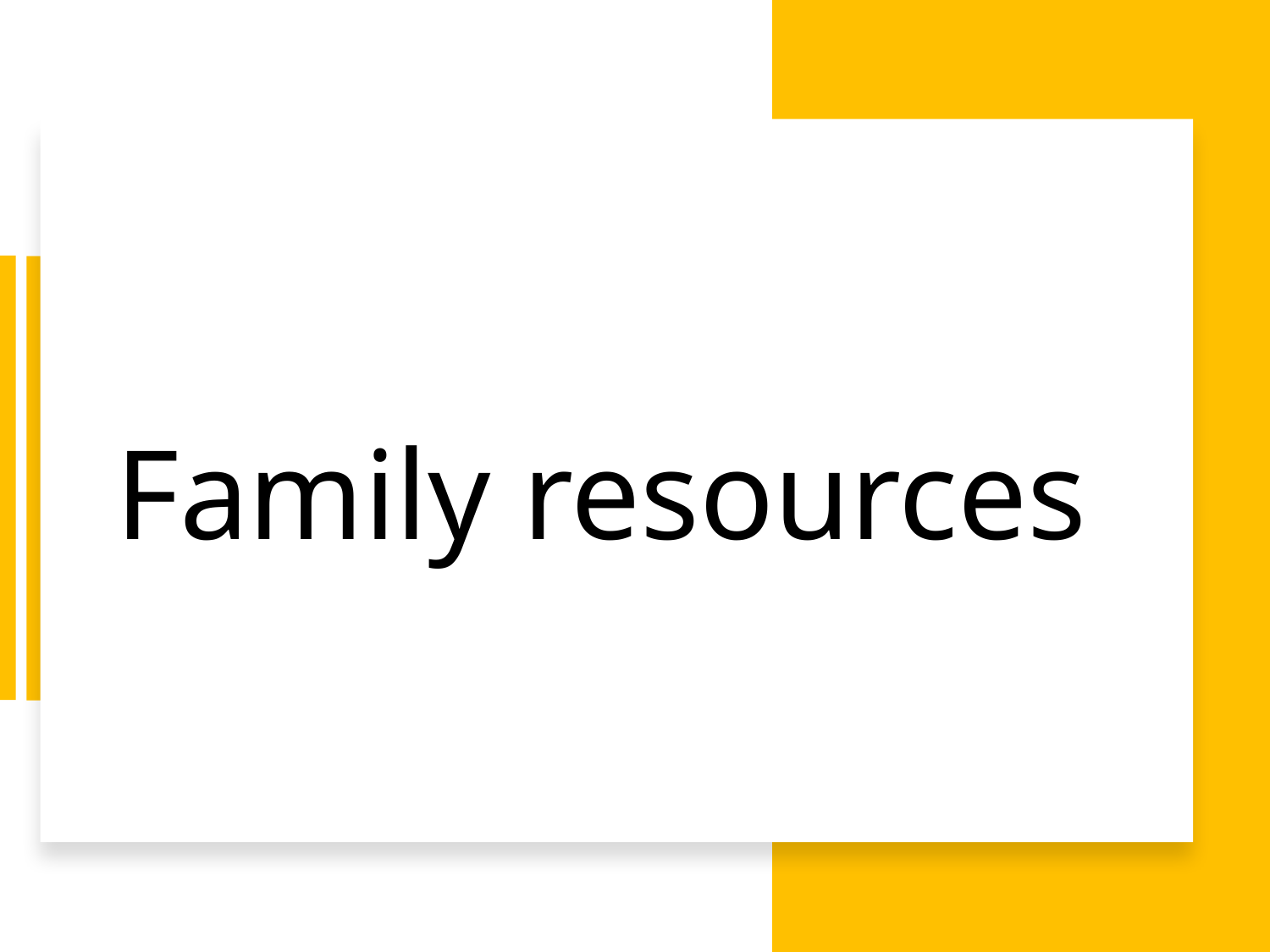

# Family resources
43

## Slide 44
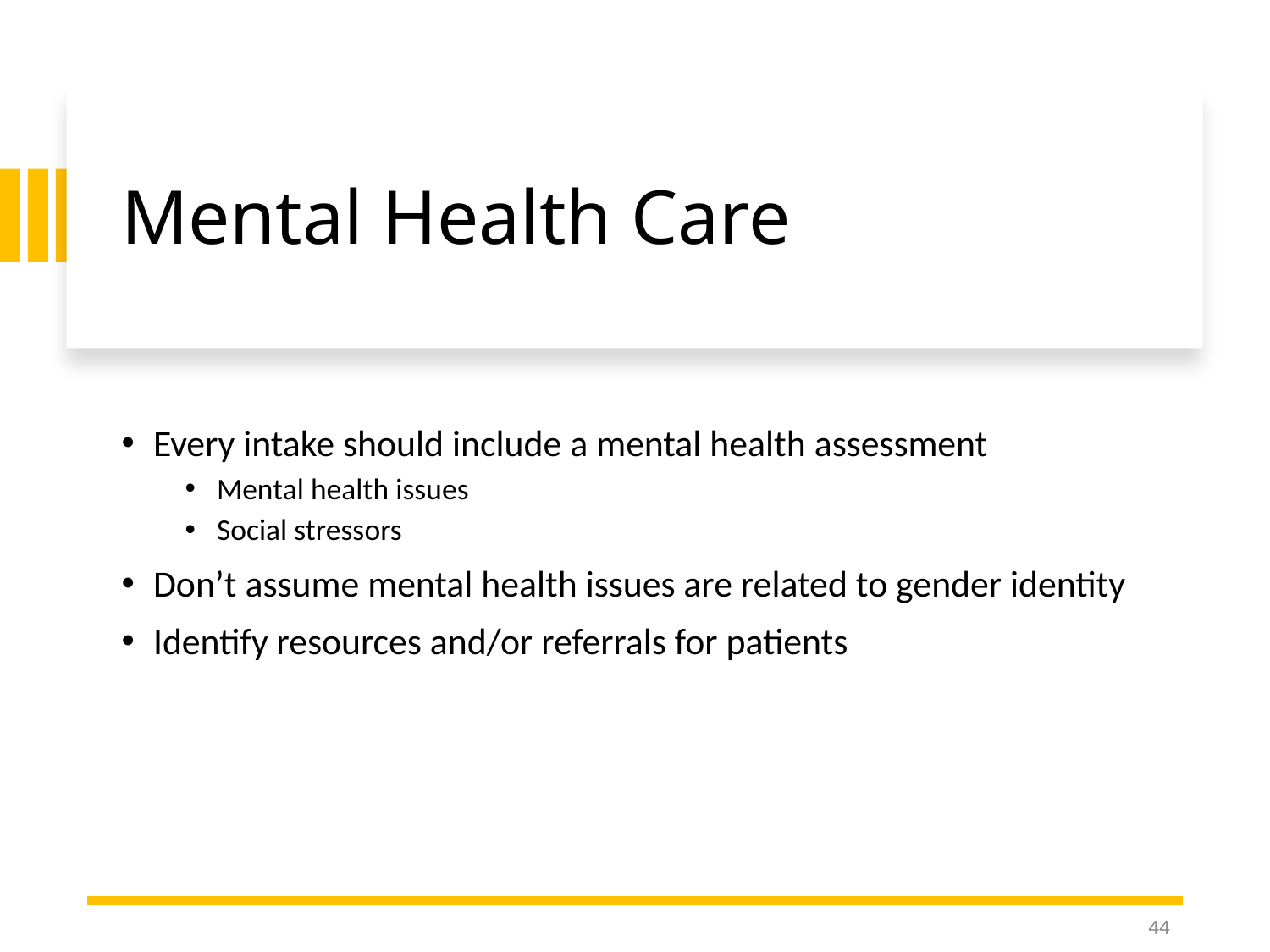

# Mental Health Care
Every intake should include a mental health assessment
Mental health issues
Social stressors
Don’t assume mental health issues are related to gender identity
Identify resources and/or referrals for patients
44

## Slide 45
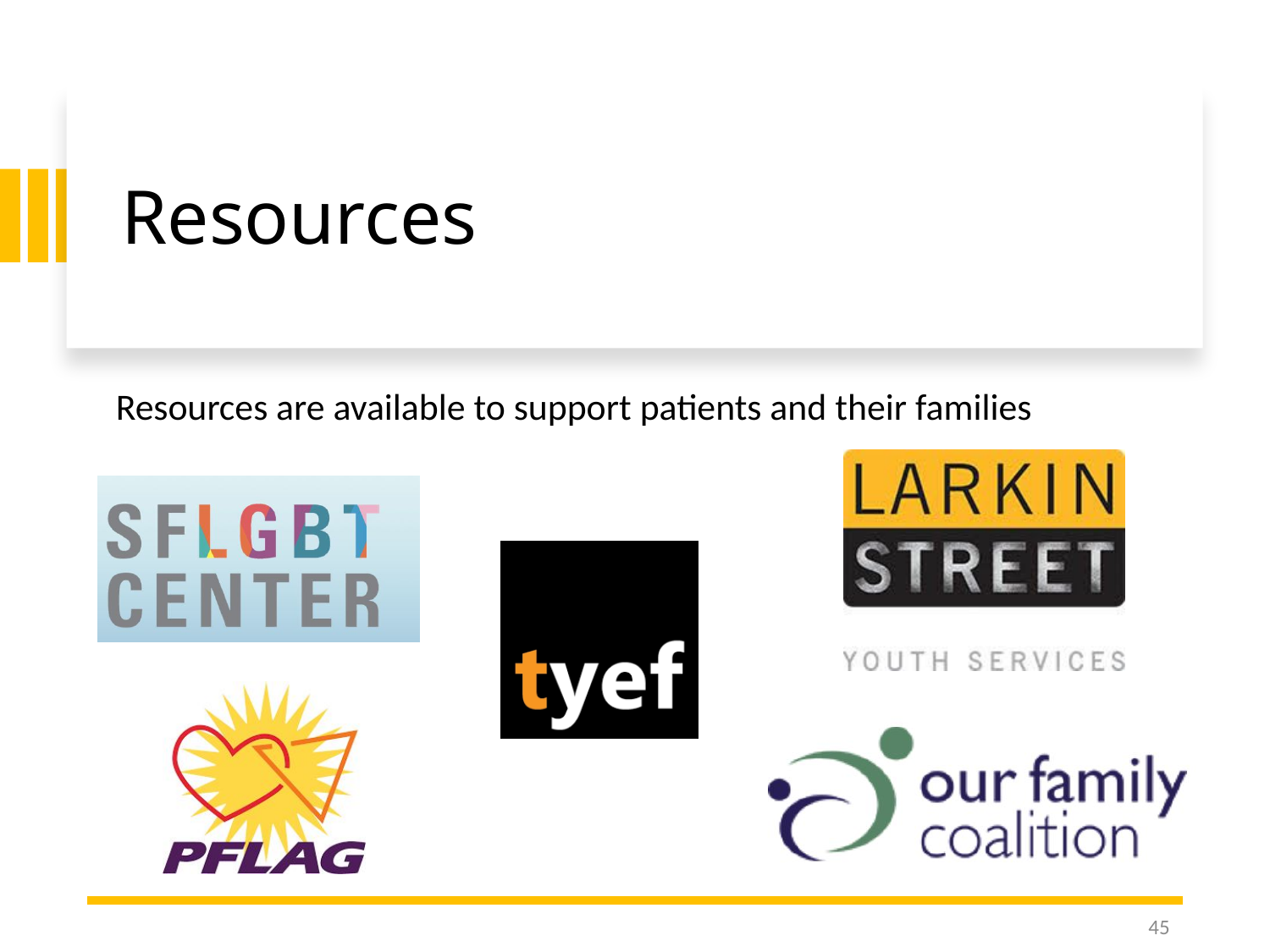

# Resources
Resources are available to support patients and their families
45

## Slide 46
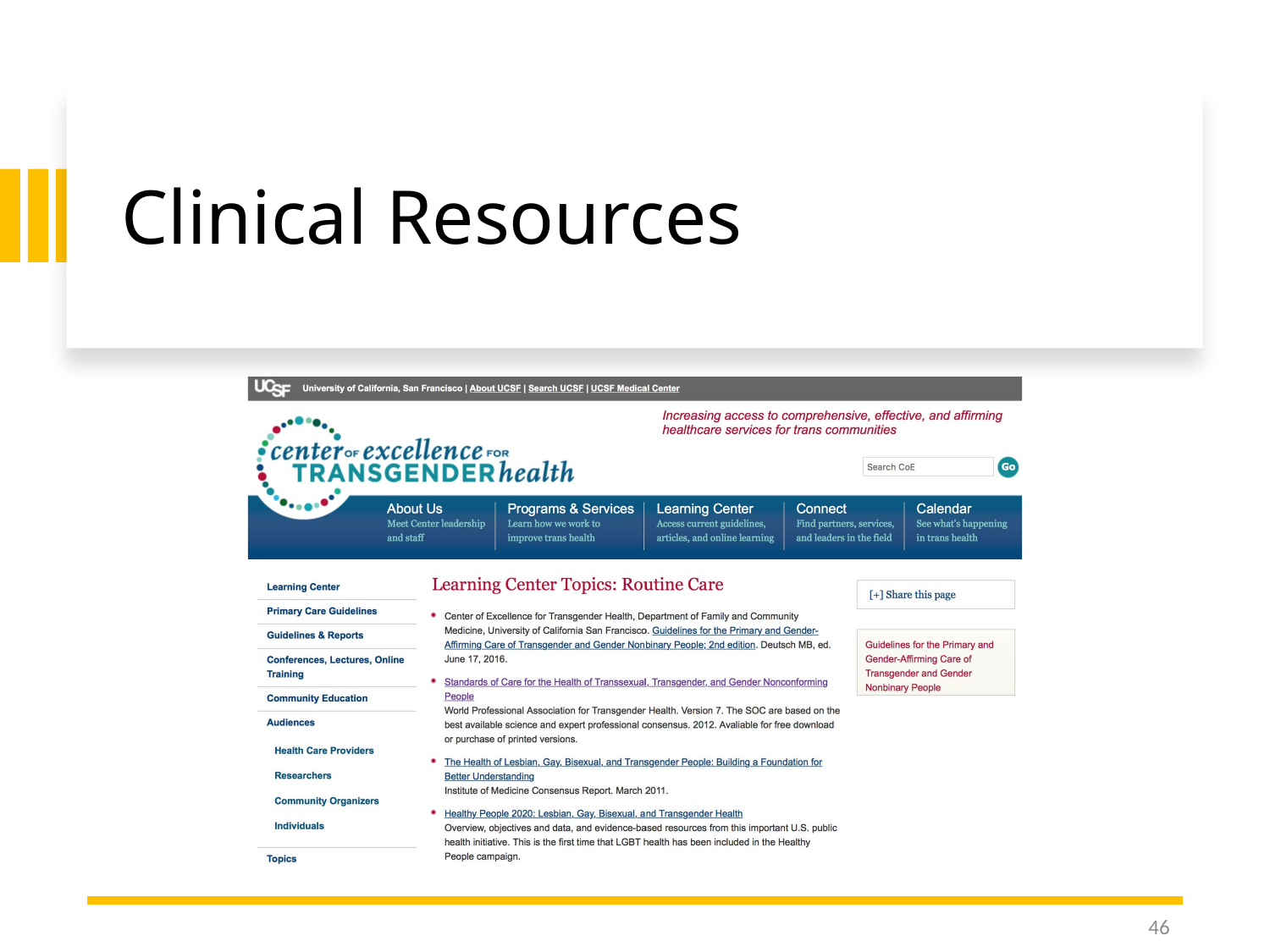

# Clinical Resources
46
